# Photocatalytic Enhancement of Anatase Supported on Mesoporous Modified Silica for the Removal of Carbamazepine

**DOI:** 10.3390/nano15191533

**Published:** 2025-10-08

**Authors:** Guillermo Cruz-Quesada, Beatriz Rosales-Reina, Inmaculada Velo-Gala, María del Pilar Fernández-Poyatos, Miguel A. Álvarez, Cristian García-Ruiz, María Victoria López-Ramón, Julián J. Garrido

**Affiliations:** 1 Department of Inorganic and Organic Chemistry, Faculty of Experimental Sciences, University of Jaén (UJA), Campus Las Lagunillas, 23071 Jaén, Spain; gcruz@ujaen.es (G.C.-Q.); mpoyatos@ujaen.es (M.d.P.F.-P.); malvarez@ujaen.es (M.A.Á.); cgruiz@ujaen.es (C.G.-R.); 2 Department of Science, Institute for Advanced Materials and Mathematics (INAMAT^2^), Public University of Navarre (UPNA), Campus Arrosadía, 31006 Pamplona, Spain; beatriz.rosales@unavarra.es; 3 Department of Inorganic Chemistry, Faculty of Farmacy, University of Granada, 18011 Granada, Spain; invega@ugr.es

**Keywords:** TiO_2_/SiO_2_ materials, hydrothermal synthesis, triethoxysilanes, photocatalysis, UV irradiation, carbamazepine removal, by-products determination

## Abstract

TiO_2_ is the most used material for the photocatalytic removal of organic pollutants in aqueous media. TiO_2_, specifically its anatase phase, is well-known for its great performance under UV irradiation, high chemical stability, low cost and non-toxicity. Nevertheless, TiO_2_ presents two main drawbacks: its limited absorption of the visible spectrum; and its relatively low specific surface area and pore volume. Regarding the latter, several works in the literature have addressed the issue by developing new synthesis approaches in which anatase is dispersed and supported on the surface of porous materials. In the present work, two series of materials have been prepared where anatase has been supported on mesoporous silica (MSTiR%) in situ through a hydrothermal synthesis approach, where, in addition to using tetraethoxysilane (TEOS) as a silicon precursor, three organotriethoxysilanes [RTEOS, where R = methyl (M), propyl (P) or phenyl (Ph)] were used at a RTEOS:TEOS molar percentage of 10 and 30%. The materials were thoroughly characterized by several techniques to determine their morphological, textural, chemical, and UV-vis light absorption properties and then the most promising materials were used as photocatalysts in the photodegradation of the emerging contaminant and antiepileptic carbamazepine (CBZ) under UV irradiation. The materials synthesized using 10% molar percentage of RTEOS (MSTiR10) were able to almost completely degrade (~95%), 1 mg L^−1^ of CBZ after 1 h of irradiation using a 275 nm LED and 0.5 g L^−1^ of catalyst dose. Therefore, this new synthesis approach has proven useful to develop photoactive TiO_2_ composites with enhanced textural properties.

## 1. Introduction

Nowadays, the overconsumption of pharmaceutical drugs, personal care products, herbicides, and other compounds in modern society has led to the presence of persistent toxic emerging contaminants (ECs) in residual wastewaters worldwide, representing an environmental issue and a matter of public health. Due to the high chemical diversity of these water pollutants and their low concentrations, conventional procedures in wastewater treatment plants (WWTPs) have been proven inefficient, with a trace-level concentration of more than fifty ECs detected in WWTPs effluents worldwide over the past few decades [1]. For instance, carbamazepine (CBZ) is a psychiatric drug widely used for the treatment of epilepsy and bipolar disorder, being the second most frequently detected pharmaceutical compound in European and African wastewater, with discrete concentrations detected as high as 559 ng L^−1^ [2]. In addition to its high consumption, once ingested, only 72% of the drug is metabolized, while the rest is excreted, ending up in wastewater [3]. Hence, CBZ has been added to the European’s Commission “Proposal for a Directive amending the Water Framework Directive, the Groundwater Directive and the Environmental Quality Standards Directive” [4].

Advanced oxidation processes (AOPs) encompass a wide range of technologies, among which photocatalysis has arisen a growing interest in recent years due to the possibility of using green and sustainable irradiation sources, such as LEDs or solar light, to induce the formation of electron (*e^−^*) and hole (*h^+^*) pairs in the conduction and valence bands of metal oxide semiconductors, respectively. The photoinduced pairs can then react directly with the ECs or promote the generation of hydroxyl (HO^•^) and superoxide (O_2_^•−^) radicals from the oxidation and reduction in water and dissolved O_2_, respectively, avoiding the need to use O_2_, H_2_O_2_ or other oxidants to generate reactive oxygen species (ROS) that degrade the ECs to smaller and harmless molecules [5]. Nevertheless, some photocatalysts present a series of inconveniences that severely affect their photodegradation performance: lack of absorption of visible light; fast *e^−^/h^+^* recombination rates; small specific surface areas; and particle aggregation [6].

Titanium dioxide (TiO_2_) is arguably the most widely used optical semiconductor as a photocatalyst for water remediation applications, due to its low cost and easy preparation, non-toxicity, chemical stability and high oxidation power [7]. However, TiO_2_ suffers from a series of aforementioned drawbacks; thus, major efforts have been made to develop modified TiO_2_ or prepare composites that can overcome such problems to obtain high photonic efficiencies [8]. Contrary to what was previously believed, insulator materials can act as robust supports by dispersing semiconductors on their surface and enhancing their photoactivity due to their chemical durability and adsorption sizes linked to their larger surface area. Moreover, insulators can act as a barrier by preventing the recombination of charge carriers (*e^−^* and *h^+^*) and photocorrosion [9]. Among the usual insulators employed for the preparation of semiconductor–insulator heterojunctions, amorphous siliceous materials (SiO_2_) stand out as a support for the dispersion of TiO_2_ nanoparticles. In addition to their high porosity which favors the interaction between the subtracts and the active centers and preventing particle aggregation, their surface silanol groups can condense with titanium precursors, anchoring TiO_2_ particles on the surface through Si–O–Ti bonds, which confers high stability to the TiO_2_/SiO_2_ hybrids [10]. Furthermore, it has been reported that the light-scattering properties of SiO_2_ can enhance the exposure of the supported TiO_2_ particles to UV radiation, improving the performance of TiO_2_/SiO_2_ hybrids compared to bare TiO_2_ [11].

The main objective of this work is to develop new TiO_2_/SiO_2_ hybrid materials with improved textural properties and photocatalytic activity. Hence, in the present work, three series of new anatase materials supported on amorphous mesoporous SiO_2_ (MSTiR%) were prepared through a hydrothermal synthesis approach using tetraethoxysilane (TEOS) and organotriethoxysilanes (RTEOS; R = methyl, M; propyl, P; and Phenyl, Ph) as silicon precursors at a molar percentage of 10% or 30% RTEOS/(TEOS + RTEOS), while titanium butoxide (TBOT) was used as the titanium precursor, keeping the Si/Ti precursors ratio of 96.86. This procedure also makes use of structure-directing agents (SDA) such as polyvinylpyrrolidone (PVP), which interact with the hydrolyzed TBOT precursors decreasing their condensation rate and favoring the co-polymerization with the hydrolyzed silicon precursors, and tetrapropyl ammonium bromide (TPABr). Unlike a previous work in which crystalline titanosilicalites were obtained using the same synthesis conditions and tetrapropyl ammonium hydroxide (TPAOH) as the SDA [12], TPABr induced the formation of TiO_2_ anatase particles of 8–10 nm anchored onto the surface of SiO_2_. RTEOS precursors also act as a tertiary SDA because, compared to TEOS, their non-hydrolysable organic moieties block a site susceptible to hydrolyze and exhort a steric effect that plays a key role in the anatase particle formation and the porosity of the resulting materials. The materials were fully characterized using a plethora of techniques such as x-ray diffraction (XRD); N_2_ adsorption; EDX-SEM (Energy-Dispersive X-ray) Spectroscopy and XPS (X-ray photoelectron spectroscopy) and UV-vis diffuse reflectance spectroscopy. The materials’ photoactivity was tested in the photodegradation of 1 mg L^−1^ of the anticonvulsant carbamazepine (CBZ) under UVC irradiation (LED system of λ_max_ = 275 nm). The influence of pH and catalyst dose was studied obtaining the best performance (98.58% degradation) when 1 g L^−1^ of MSTiP10 was used at non-controlled pH (pH = 6.0). The transformation products of CBZ were analyzed using LC-QTOF and a photodegradation pathway was proposed based on the results and the reviewed literature, and phytotoxicity tests were performed to assess the lower toxicity of the treated water. Thus, the most novel and innovative aspect of this work is the development of new TiO_2_/SiO_2_ hybrid materials with improved textural properties and good photocatalytic activity despite containing a low amount of Ti (0.52–1.36 wt% determined by EDX), by using triethoxysilanes in an innovative synthesis approach that favors the formation of superficial anatase.

## 2. Materials and Methods

### 2.1. Reactives

TEOS (Tetraethoxysilane, assay ≥ 99%) and TBOT (Titanium butoxide, assay 97%) were the respective silicon and titanium precursors used in this study. The used hybrid silicon precursors (RTEOS) were MTEOS (triethoxymethylsilane, assay 99%), PTEOS ((n-propyl)triethoxysilane, assay 97%) and PhTEOS (phenyltriethoxysilane, assay 98%). The organic substances used as surfactants in the synthesis were TPABr (tetrapropylammonium bromide, assay 98%) and PVP (poly(vinylpolipirrolidone) ~110 µm particle size). Carbamazepine (5H-Dibenz[b,f]azepine-5-carboxamide, CBZ, assay 98%) was selected to assess the photocatalytic activity of the materials. All reactants were purchased from Sigma-Aldrich (St. Louis, MO, USA). Titanium(IV) oxide (Degussa Aeroxide P25) was purchased from Acros Organics, and used to acquire a diffraction pattern serving as a reference for the detection of TiO_2_ (anatase and rutile) diffraction maxima. CBZ is a polar molecule with a high electrophilicity value. The molecule remains neutral at the 3 ≤ pH ≤ 11 range [13], although it can form molecular aggregates, mainly dimers, through hydrogen-bonding or aromatic interactions [14,15]. Table S1 contains the more relevant chemical and physical properties of carbamazepine.

### 2.2. Synthesis of the Anatase Supported on Mesoporous Silica (MSTiR%)

**Figure 1:**
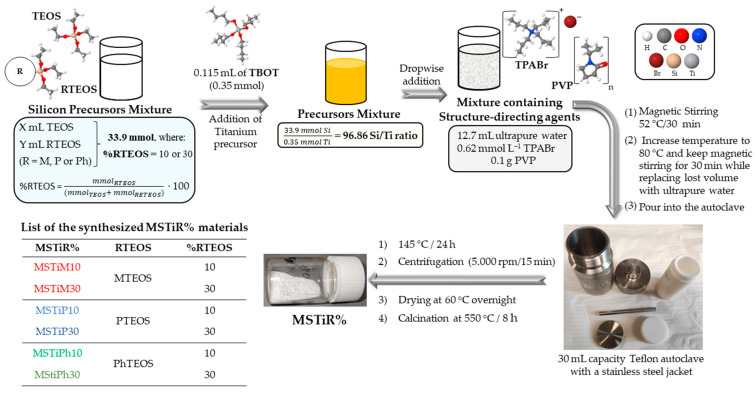
Synthesis procedure of the anatase supported on mesoporous silica (MSTiR%).

This synthesis method was employed in a previous work to prepare modified titanosilicalites [12]. Nevertheless, for this work TPABr was used instead of TPAOH (tetrapropylammonium hydroxide) to obtain different materials. Figure 1 shows a diagram of the synthesis of the materials.

Briefly, six mixtures of 33.9 mmol of silicon precursors were prepared with TEOS and one of the three triethoxysilane (RTEOS, R = M, P, or Ph) in a RTEOS/(TEOS+RTEOS) molar percentage equal to 10% or 30%. In all cases, 0.115 mL of TBOT (0.35 mmol) was added to the silicon precursor mixture, which was successively added dropwise to a 12.7 mL aqueous solution containing TPABr (0.62 mol L^−1^) and PVP (0.1 g). The resulting mixture was stirred at 52 °C for 30 min on a thermostatic magnetic plate. When the solution was homogenized, the temperature was increased to 80 °C to remove the alcohols generated during the hydrolysis of the precursors and water was continuously added over 30 min to keep the volume constant. Afterwards, the mixture was poured into a 30 mL Teflon autoclave with a stainless steel jacket (Huanyu high-tech Co., Ltd., Wenzhou, China) which was maintained at 145 °C for 24 h. After the hydrothermal process was completed, the white gelatinous solid was dispersed in water and centrifuged (using a Sorvall ST 8 centrifuge, Thermo Fisher Scientific, Waltham, MA, USA) at 5000 rpm for 15 min. Finally, the samples were collected, dried at 60 °C overnight, and calcinated at 550 °C for 8 h in a muffle furnace (12PR/300 Hobersal Furnace, Barcelona, Spain), obtaining white solids. The Si/Ti precursor ratio for all syntheses was 96.86, which means that titanium precursors represent only ~1% of the molar percentage of metal oxide used as a precursor.

### 2.3. Characterization Techniques

This section gathers a brief description of the techniques and equipment employed for the characterization of the materials. For detailed measurements and calculation methods, see Section S1 of the Supplementary Material.

X-ray diffraction patterns were obtained at room temperature, using a PANalyticalEmpyrean XRD instrument (Empyrean, Almelo, The Netherlands) with copper rotating anode and graphite monochromator (at 45 kV and 40 mA) to select the CuK_α1/2_ wavelength of the incident beam at 0.154 nm. Infrared spectra were obtained using a FTIR spectrometer (Jasco mod. 4700, Tokyo, Japan). Ultraviolet-Visible diffuse reflectance spectra (UV-vis DRS) were acquired in the measurement range of 200–400 nm at 25 °C. The samples reflectance (R) and absorbance were recorded in a UV-visible-NIR Varian spectrometer (model CARY-5E, Agilent technologies Spain S.L., Barcelona, Spain) equipped with a spherical diffuse reflectance accessory, where BaSO_4_ was used as the reflectance standard due to its 100% reflectance in the visible region. The surface atomic composition and the valence band maximum edge potential (*E*_VBM_) of the materials were determined by X-ray photoelectron spectroscopy (XPS). The spectra were recorded with a constant pass energy value of 29.35 eV, 0.125 eV step^−1^ and a beam diameter of 200 µm using a physical electronic spectrometer (PHI Versa Probe II, Physical Electronics, Minneapolis, MN, USA) with monochromatic X-ray Al K_α_ radiation (15 kV, 1486.6 eV), a hemispherical multichannel detector, and a dual beam charge neutralizer for analyzing the core-level signals of the elements of interest which were C1s, O1s, O2s, Si2s, Si2p and Ti2p. The position and areas of the peaks were determined using the C1s adventitious carbon peak at 284.6 eV as an internal standard. N_2_ adsorption isotherms (−196 °C) were determined with a volumetric adsorption system (ASAP2020, Micromeritics, Norcross, GA, USA), weighing approximately 150 mg of sample into a straight-walled Pyrex glass tube followed by degassing at 200 °C for ≤2 h with a residual vacuum of <0.66 Pa. Analysis time ranged from 14 to 55 h. The sample tube was covered with an isothermal jacket and immersed in a Dewar with liquid nitrogen (−196 °C). Field-Emission Scanning electron microscopy (FE-SEM) micrographs with a magnification of 1.00, 6.00 and 48.00 KX were obtained at 15.00 kV using a Carl Zeiss microscope (model Merlin, Carl Zeiss Iberia, S.L., Tres Cantos, Spain). The system was equipped with an energy-dispersive X-ray (EDX) detector (model 350X-MAX 50, Oxford Inca, High Wycombe, **UK**). The samples were placed on carbon conductive tapes and coated with gold to reduce any charge effect, as SiO_2_ matrices are non-conducting.

### 2.4. Photodegradation Experiments

The photodegradation experiments of CBZ were conducted in ultrapure water (18.2 MΩ cm), with 100 mL of contaminant solution (1 mg L^−1^) in a 120 mL cylindrical glass reactor (2.6 cm diameter) under magnetic stirring (2500 rpm). The experiments were performed using catalyst doses of 0.25, 0.5, 0.75 and 1 g L^−1^, and under 2 h of irradiation. The irradiation system was composed of 8 LEDs (Luminus, mod XFM-5050-UV-D130-FE270-00, Sunnyvale, CA, USA) emitting in the 270–280 nm wavelength range at the minimum lighting power of the flux (200 mW) each. The total irradiation was ~104 W/m^2^ and cooling fans were used during the entire reaction time to keep the temperature constant. The LED system and the emission spectra of the LEDs provided by the manufacturer are shown in Figure S1 of the Supplementary Material. During the experiments, aliquots of 1 mL were collected with a 5 mL syringe using a 0.45 µm Nylon filter and poured into HPLC inserts. For the reuse experiments, the catalysts were recovered by vacuum filtration using a Dinko (Mod D-95) pump. The CBZ concentration of the aliquots was determined by Ultra-High-Pressure Liquid Chromatography (HPLC) using a Shimadzu series 20 apparatus with an SPD-M20A diode array detector, equipped with a Hypersil GOLD C18 (250 mm × 4.6 mm) column. An isocratic method with a mobile phase of 40/60 *v*/*v* acetonitrile/ultrapure water (with 1% formic acid) at a flow rate of 1 mL min^−1^ was used. The excitation wavelength for CBZ was 286 nm.

The CBZ degradation by-product analysis was performed using an ultra-pressure liquid chromatograph UPLC (Waters, Acquity H-Class model, Milford, MA, USA) with a C18 column (2.1 × 100 nm, 1.7 μm) coupled with a quadrupole time-of-flight mass spectrometer (Waters, Xevo TQ-S model, USA) equipped with a positive electrospray ionization system (UPLC/Q-TOF-MS-ES+). To study the toxicity of the treated CBZ solution, phytotoxicity tests were performed using the Phytotoxkit provided by Microbiotests (Gent, Belgium). A batch of 10 seeds for each of the 3 different fast-growing plant species, *Sinapis alba* (S.A.), *Sorghum saccharate* (S.S.), and *Lepidium sativum* (L.S.), were watered with treated solutions. The length of the roots and stems of the germinated seeds were measured after 3 days growing in the dark and 25 °C, and compared to those of the reference batches watered with miliQ water and the solution of 1 mg L^−1^ of CBZ.

## 3. Results and Discussion

### 3.1. Characterization Analysis of the MSTiR% Materials

#### 3.1.1. X-Ray Diffraction (XRD) and Fourier-Transformed Infrared Spectroscopy (FT-IR)

**Figure 2:**
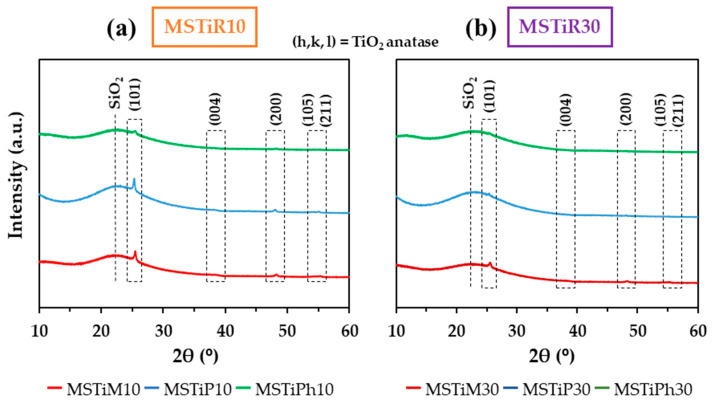
Diffraction patterns of the (**a**) MSTiR10 and (**b**) MSTiR30 materials.

Figure 2 depicts the diffraction patterns of the materials recorded in the 10° < 2θ < 60° range. Figure S2 shows the XRD pattern zoomed in 35–40°, 45–50°, and 50–60°; and Table S2 contains the identified diffraction maxima with their corresponding intensity, Full Width at Half Maximum (FWHM) and integrated areas.

No diffraction maxima belonging to the orthorhombic MFI lattice planes of the titanosilicalites (7.8°, 8.9°, 23.1°, 24°, and 24.5°) were detected [16,17]. However, a broad band (maximum intensity at 2θ ~22°) belonging to amorphous silica is recorded in all materials’ diffraction patterns. This confirms that TPABr could not replace the role of TPAOH in directing the formation of the MFI structure during the hydrothermal synthesis. Hence, in the absence of an MFI lattice, Ti^+4^ is not forced to occupy tetrahedral positions, resulting in the formation of TiO_2_ particles. This is confirmed by the diffraction patterns of MSTiM10, MSTiP10 and MSTiM30, whose four maxima correspond to the (101), (200), (105) and (211) lattice planes of anatase, according to The International Centre for Diffraction Data (ICDD, JCPDS, file code 21-1272) and by the XRD pattern of commercial TiO_2_ Degussa P25 Aeroxide (Figure S3), where the lattice plains of anatase and rutile have been indicated to ease comparison with MSTiR% samples patterns. Nevertheless, as is shown in Figure S2, the maximum associated with the (004) lattice plain was not observed in any sample, and (105) and (211) lattice plains were only observed in the MSTiM10, MSTiP10 and MSTiM30 materials. This denotes that only a small proportion of TiO_2_ is present in the samples, because the organic groups of the RTEOS precursors prevent the anchoring of TiO_2_ on the silica surface due to the steric effect exerted by these groups, hindering the co-condensation between the silicon precursor and titanium. In fact, the XRD patterns of MSTiPh10 and MSTiPh30, the materials synthesized with the bulkier organic group, only showed the (101) and (200) lattice plains due to the low amount and crystallinity of their TiO_2_ nanoparticles. The presence of rutile is discarded since no maxima at 2θ ~ 27.65 and 36.02° (corresponding to its (110) and (101) lattice plains, respectively) were identified in any pattern of the materials [18]. Table 1 depicts the 2θ of each detected diffraction maximum of anatase and the determined structural parameters of each MSTiR% material: the inter-planar distances (*d*_(h, k, l)_) calculated from the Bragg equation (Equation (S1)); the crystallite size (*D*_(h, k, l)_) calculated from the Scherrer equation (Equation (S2)); and the degree of crystallinity (calculated from Equation (S3)).

Table 1 shows that the materials present crystallite sizes around ~9 nm. The degree of crystallinity decreases when the percentage of RTEOS increases from 10 to 30%, indicating that the organic precursors hinder the formation of the crystalline anatase particles. Additionally, it is also noteworthy that the bulkier their organic moieties are (propyl and phenyl in PTEOS and PhTEOS, respectively), the lower the degree of crystallinity.

Figure S4 shows the infrared spectra of the MSTiR% samples in the 4000–2750 and 1600–400 cm^−1^ spectral range. The identification of the characteristic bands can be found in Section S2 of the Supplementary Material. It is worth mentioning that the low intensity recorded for their Si–O (955 cm^−1^) and ν Si–OH–H (3660 cm^−1^) bands indicates that the MSTiR% materials are less hydrophile than expected, considering the that the materials have been synthesized in basic medium (due to the presence of TPABr); however, this is a consequence of the high calcination temperature that induces the condensation of a considerable portion of the surface Si–OH groups [19].

#### 3.1.2. UV-Vis Diffuse Reflectance (DR) and X-Ray Photoelectron Spectroscopy (XPS)

**Figure 3:**
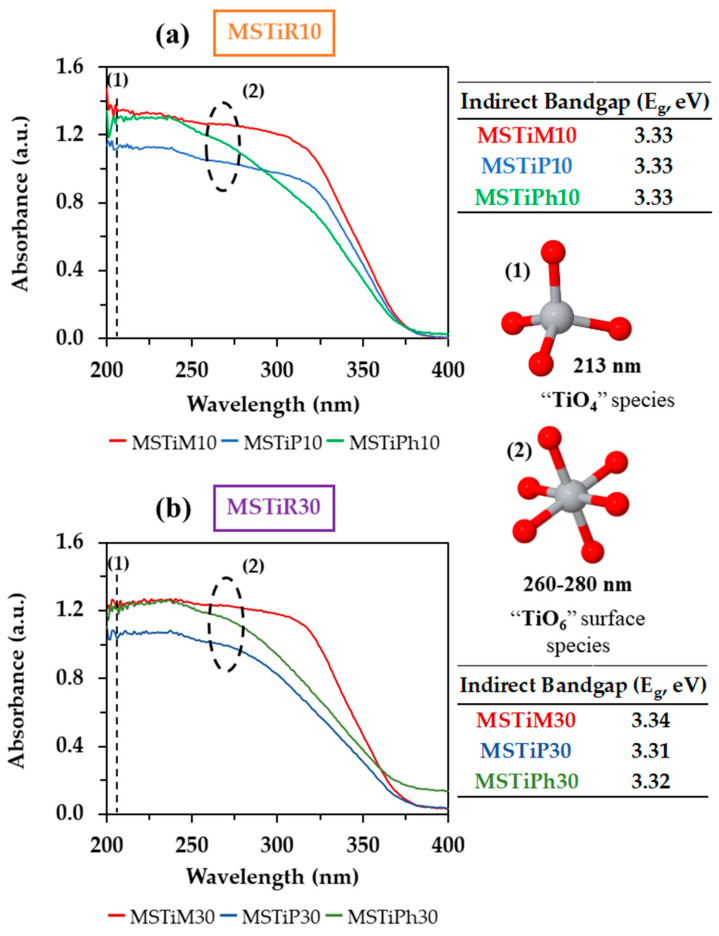
UV-vis DR spectra of (**a**) MSTiR10 and (**b**) MSTiR30 materials in the 200–400 nm spectral range with their calculated indirect bandgap energy (*E*_g_).

Figure 3 shows the UV–Vis DR spectra of the MSTiR% materials in the 200–400 nm spectral range.

The UV-vis DR spectra of the samples indicate that the only photoactive species in the materials are the hexa-coordinated TiO_2_ species (TiO_6_), since no absorption peak of the tetra-coordinated species (TiO_4_, typical of titanosilicalites) was observed in any spectra at ~210 nm (O^−2^–Ti^+4^, charge–transfer). The absorption of the TiO_6_ species starts at 260 nm and decreases until reaching 380 nm, anatase particles absorb in the range of 260 to 280 nm while surface TiO_6_ absorbs mainly at 330 nm [20]. Therefore, since the absorbance of MSTiPh% materials decays at a lower wavelength and they have the lowest degree of crystallinity, it can be stated that materials synthesized using the PhTEOS precursor contain fewer photoactive centers than those synthesized using MTEOS or PTEOS.

The spectra in Figure 3 also provide useful information about the electronic structure of the materials. For instance, the optical bandgap of the materials (*E*_g_) was obtained applying the Tauc-plot method (Equation (S4)–(S6)), considering that the materials are indirect semiconductors (Figure S5). The calculated *E*_g_ values are all below 4 eV, indicating that these materials can act as photocatalysis since they are semiconductors capable of generating charge carriers when irradiated [21]. However, no appreciable difference was detected between the MSTiR% materials since all have Eg ~ 3.30 eV, a value close to that reported for 5–10 nm anatase particles (*E*_g_ = 3.20 eV) [22].

XPS spectra of the MSTiR% materials were acquired, and peaks corresponding to adventitious carbon (C1s), oxygen (O2s and O1s), and silicon (Si2p and Si2s) were identified, although the peak corresponding to Ti2p was only identified in the MSTiPh10 spectrum (Figure S6). Nevertheless, Table 2 shows the surface atomic composition of the materials estimated using this technique.

The relative abundances of silicon and oxygen in Table 2 are consistent with silicon dioxide materials. Titanium was identified on the surface of the materials in low quantities, with MSTiPh10 presenting the highest amount (0.77 wt%). The lower amount of Ti in MSTiR30 materials (0.25–0.45 wt%) compared to their MSTiR10 analogs (0.45–0.77 wt%) is also notable and indicates that a higher proportion of RTEOS precursor inhibits titanium incorporation, thereby decreasing the number of reactive sites and the subsequent photocatalytic activity of the materials. The amount of carbon represented in Table 2 for each sample corresponds to adventitious carbon.

**Figure 4:**
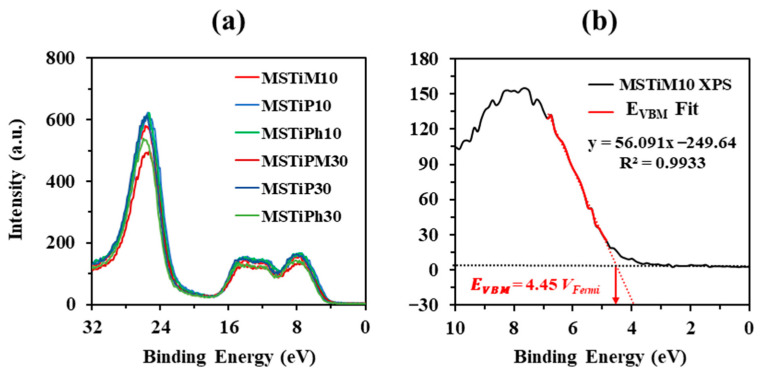
(**a**) XPS low binding energy spectra of the MSTiR% materials; and (**b**) linear fit of the MSTiM10 spectrum to determine its valence band maximum edge potential (*E*_VBM_).

The valence band maximum edge potential (*E*_VBM_) of the MSTiR% materials was determined form the low binding energy region of the XPS spectra (Figure 4a) by applying a linear fit. For instance, Figure 4b depicts the fit to determine the *E*_VBM_ of MSTiM10 from its XPS spectrum (the linear fit of the other five MSTiR% materials can be found in Figure S7).

The determined *E_VBM_* values are relative to the Fermi level (*V*_Fermi_), thus Equation (S7)–(S9) were used to obtain the potentials vs. the vacuum level (*V*_Vacuum_), and then Equation (S10) was applied to determine the conduction band minimum edge potential (*E*_CBM_) considering the *E_g_* estimated from the Tauc-plot method applied to the UV-Vis DR spectra. The values of *E*_VBM_ and *E*_CBM_ relative to the Normal Hydrogen Electrode (NHE, at 25 °C and pH = 0) were calculated using Equation (S11). Table 3 shows the *E*_VBM_ and *E*_CBM_ potentials of the MSTiR% materials determined in *V*_Vacuum_ and *V*_NHE_ values.

The *E*_VBM_ and *E*_CBM_ values of the MSTiR% materials differ on average by 1.86 and 1.73 eV, respectively, from those of pure anatase (−7.25 and −4.05 *V*_Vacuum_ or 2.81 and −0.39 *V*_NHE_, respectively) [23]. This is a consequence of the shift in band positions, since, as observed in their XRD patterns (Figure 2), these materials contain a small proportion of crystalline anatase supported on amorphous SiO_2_, which is an insulator with a wide bandgap of ~9 eV. In fact, it has been reported that the dipole interface can cause a relative alignment of 0.5, 1.1 or 1.6 eV of the band shift in Si/SiO_2_, SiC/SiO_2_ and GaN/SiO_2_ materials, respectively [24]. MSTiM10 and MSTiM30 presented similar *E*_VBM_ and *E*_CBM_ values, consistently with MTEOS being the organosilane that least reduced the anatase crystallinity when the RTEOS/TEOS molar percentage was increased to 30% (Table 1). Conversely, the lower crystallinity of MSTiP30 and MSTiPh30 results in a broader shift in the value of the conduction bands compared to their MSTiR10 analogs. To ease the visual comparison, Figure S8 depicts a scheme of the band positions of MSTiM10 (as an example of the MSTiR10 materials), pure anatase (−0.39 and +2.81 V vs. NHE for its CB and VB, respectively [23]), and the required redox potentials to generate HO^•^ (E° (H_2_O/HO^•^) = 2.72 and E° (HO^−^/HO^•^) = 1.99 V_NHE_) and O_2_^•−^ (E° (O_2_/O_2_^•−^) = −0.33 V_NHE_) radicals [25], which are the main species degrading organic pollutants during photocatalytic processes. The diagram of Figure S8 indicates that since the *E*_VBM_ values of MSTiM10 and pure anatase are more positive than those of *E*° (H_2_O/HO^•^) and *E*° (HO^−^/HO^•^), both materials can generate HO^•^ radicals when irradiated. Nevertheless, unlike pure anatase, the generation of O_2_^•−^ radicals by MSTiR% materials is thermodynamically unfavorable, since their *E*_CBM_ is more negative than that of E° (O_2_/O_2_^•−^). Therefore, compared to pure anatase, the photoactivity of MSTiR% materials is more limited, and the main photogenerated reactive species are the OH^•^ radicals.

#### 3.1.3. N_2_ Adsorption Isotherms (−196 °C)

**Figure 5:**
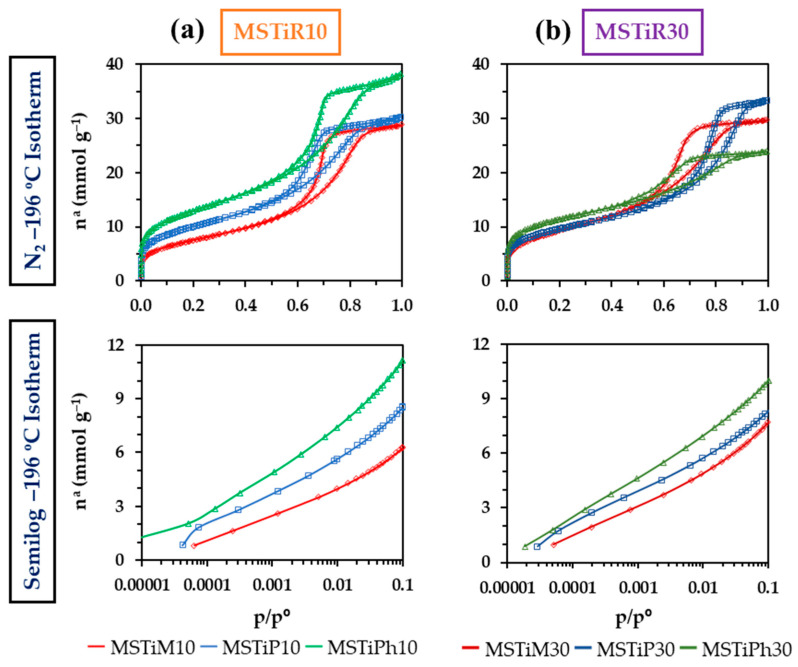
N_2_ isotherms at −196 °C of the (**a**) MSTiR10 and (**b**) MSTiR30 materials in normal (up) and semilogarithmic scales (down).

Figure 5 depicts the N_2_ isotherms at −196 °C of the MSTiR% materials, all of which are type IV(a) isotherms characteristic of mesoporous materials, as indicated by their fully open knee at low partial pressures (*p*/*p*°), the hysteresis loop, and a plateau at high *p*/*p*°. The hysteresis loop, located in the range of 0.6–0.85 *p*/*p*° can be classified as type H2(b), associated with pore blocking occurring in pores with a large distribution of pore neck sizes, as is the case of mesoporous ordered silicas synthesized through hydrothermal methods [26]. This contrasts with the isotherms of Figure S9, which belong to materials prepared in a previous work where the procedure described in Figure 1 was used employing TPAOH instead of TPABr [12]. For these materials, when 10% RTEOS was used, Type VI isotherms typical of micro-mesoporous zeolites were obtained, while when 30% RTEOS was used, Type II isotherms corresponding to amorphous materials with no significative micro or narrow mesoporosity were obtained.

Figure 5 shows that the N_2_ adsorption capacity of the MSTiR10 materials can be ranked as MSTiPh10 > MSTiP10 and MSTiM10; however, this trend is not followed in the MSTiR30 materials where MSTiP30 adsorbed more N_2_ than MSTiPh30. Nevertheless, the isotherms in the semilogarithmic scale of Figure 5 allow a clearer observation of the low partial pressure range (1 × 10^−5^ to 0.1), where it can be noted that for both MSTiR10 and MSTiR30, the MSTiPh% > MSTiP% > MSTiM% trend is followed. This indicates that PhTEOS precursors induce the formation of a greater number of micropores in the materials, while PTEOS promotes the formation of wider pores. The textural parameters obtained from the isotherm data and applying the BET, Dubinin–Radushkevich, and BJH models are shown in Table 4.

**Figure 6:**
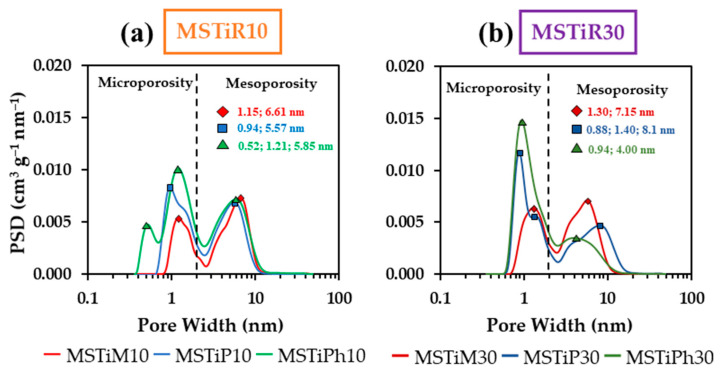
DFT-calculated pore size distribution (PSD) of the (**a**) MSTiR10 and (**b**) MSTiR30 materials in N_2_ adsorption isotherms at −196 °C.

As indicated in Table 4, in MSTiR10 materials, the precursors with bulkier organic moieties have induced the formation of a higher specific surface area (*a*_BET_), volume of micropores (*V*_micro_) and total volume of pores (*V*_Total_). The same trend is observed in MSTiR30 materials, but when compared to the MSTiR10 materials, (a) MSTiM10 and MSTiM30 have similar textural parameters; this can be explained by the low steric hindrance exerted by the methyl groups, which apparently had a lower impact on the polymerization process and the consequent formation of the porous polymeric network; (b) MSTiP30 presents a lower *a*_BET_ than MSTiP10 due to its lower volume of mesopores (*V*_meso_), but its *V*_Total_ is higher due to a higher proportion of macropores (*V*_macro_), which explains its larger average pore size calculated by BJH method (BJH APS); and (c) all textural parameters of MSTiPh30 are lower than those of MSTiPh10. Figure 6 depicts the DFT-calculated pore size distribution (PSD) of the MSTiR% materials.

The PSDs in Figure 6 show the accumulated pore volume with respect to the pore width, where all samples present at least two maxima, one belonging to micropores at ~1 nm and the others belonging to mesopores at 5–8 nm (MSTiPh10 and MSTiP30 present and additional maxima below 1 nm). As the molar percentage of RTEOS increased, the accumulated pore volume at ~1 nm increased while it decreased at 5–8 nm in the MSTiP% and MSTiPh% materials, confirming that increasing the molar percentage of RTEOS results in more microporous materials. However, it is worth mentioning that unlike MSTiP% and MSTiPh% materials, those synthesized with MTEOS present a higher value accumulated pore volume value at 6–7 nm than at ~1 nm, remarking that these materials are the more mesoporous among the synthesized materials. Therefore, comparing the effect of RTEOS precursors, it can be concluded that MTEOS favors mesopore formation, PTEOS induces the macropore formation at higher molar percentages, and PhTEOS induces the formation of a higher proportion of micropores. Hence, contrary to the methyl groups of MTEOS, the steric effect of the propyl and phenyl groups of PTEOS and PhTEOS, respectively, hinders the formation of the porous polymeric network, resulting in less mesoporous materials. As observed in Table 1, the TiO_2_ ~9 nm crystallite size (*D*_(101)_) is larger than the BJH’s average pore size (APS in Table 4) and mesopore sizes observed in the PSD of the materials, demonstrating that the anatase particles could not have been formed inside the pores of the materials and are instead supported on their surface. In fact, analysis of the UV-vis spectra of the materials revealed that PTEOS is the precursor that most favors the formation of photoactive TiO_2_ nanoparticles, which may be related to the fact that PTEOS is the precursor that induces the formation of a higher proportion of macropores. Regarding the CBZ adsorption capacity of the materials, their pore size distributions and BJH APS confirmed that CBZ with its 0.37 Stokes radius (Table S1) would not present any steric restriction to access and diffuse through their wide micropores and mesopores, thus the chemical affinity between their surface groups and CBZ would be the key factor.

#### 3.1.4. Field-Emission Scanning Electron Microscopy (FE-SEM) and X-Ray Energy Dispersion (EDX) Analyses

**Figure 7:**
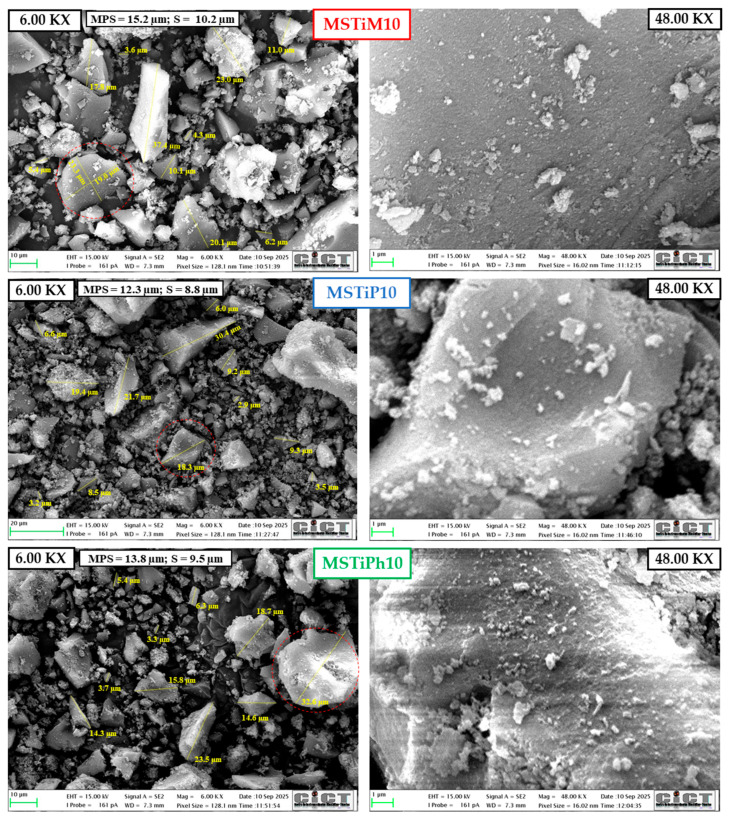
FE-SEM micrographs of the MSTiR10 materials obtained at magnifications of 6.00 KX and 48.00 KX (particles in red circles in 6.00 KX micrographs). MPS = mean particle size; S = standard deviation.

Figure 7 and Figure S10 show FE-SEM micrographs obtained from the MSTi10% and MSTi30% materials, respectively, at magnifications of 6.00 and 48.00 KX. Micrographs of the MSTiR% materials acquired at magnification of 1.00 KX are shown in Figure S11.

**Figure 8:**
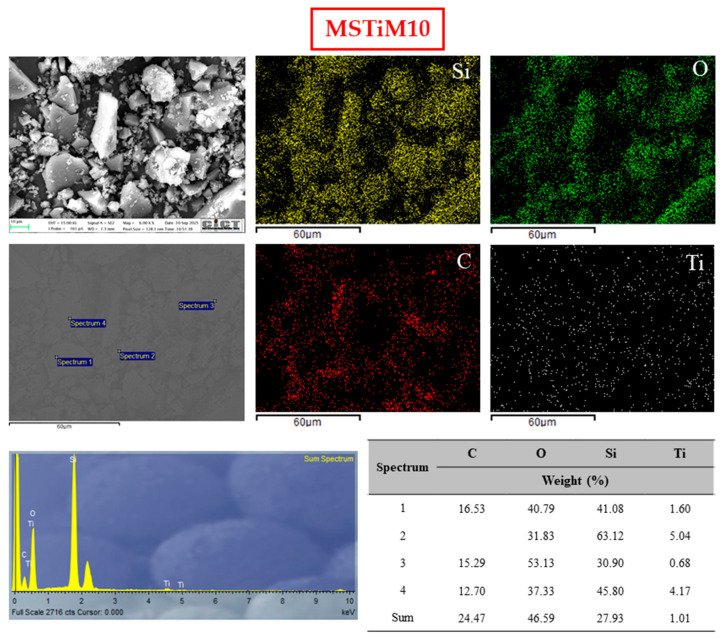
EDX mapping analysis of the MSTiM10 6.0 KX micrograph and relative abundances of C, O, Si and Ti calculated from the acquired spectra of different particles and from their sum spectra.

The micrographs in Figure 7, Figures S10 and S11 confirmed that the MSTiR% materials are amorphous micromaterials whose particles do not present any predominant shape or characteristic and have a wide range of particle sizes (~3–150 µm). Multiple particles between 3 and 30 µm were measured in the micrographs obtained at a magnification of 6.00 KX (Figure 7 and Figure S10), while particle sizes ranged from 25 to 150 µm were measured in micrographs acquired using a magnification of 1.00 KX (Figure S11). The morphology of selected particles (circled in red in the 6.00 KX micrographs) was studied at 48.00 KX magnification, where the particles displayed rough surfaces that are consistent with the microporosity and narrow mesoporosity observed in their isotherms. Figure 8 shows the EDX mapping of the MSTiM10 6.00 KX micrograph, and the relative abundances (wt%) of carbon, oxygen, silicon and titanium estimated from the EDX spectra acquired at different spots of the micrograph. Figures S12–S16 shows the mapping of the other MSTiR% materials.

EDX mapping of the of all materials shows that they contain a uniform distribution of silicon and oxygen atoms, and carbon from the calcination of the organic precursor and structure-directing agents. Titanium is present in all samples in low quantities, homogeneously distributed in the micrograph according to the mappings. However, spot EDX analysis of several particles (displayed in the 6.00 KX micrographs) revealed high variability in their Ti concentration, with particles containing up to 5 wt% and others in which no Ti was detected. Furthermore, titanium was not preferentially found in particles of any specific size. Table 5 shows the relative abundances (C, O, Si and Ti) obtained from the EDX sum spectra of the 6.00 KX micrographs, and the mean particle size of each material considering the particle length measured in the 1.00 and 6.00 KX micrographs.

According to Table 5, the bulk MSTiR% materials contain a relative abundance of titanium of 0.52–1.36 wt%, which is a low amount as expected from the silicon/titanium precursor molar ratio of 96.86 used in the synthesis. While materials synthesized with PhTEOS contain a similar amount of titanium, materials prepared with 30% of MTEOS and PTEOS have a significantly lower amount, as was the case with the abundance determined on the surface by XPS (Table 2). The last column of Table 5 shows the ratio of Ti on the surface and in the bulk of MSTiR% materials. This ratio indicates that, in all materials, more than one-third of the Ti species are located at the surface, with MSTiPh10 having the highest proportion, consistent with its larger specific surface area. However, MSTiP10 has the highest Ti mass content (1.36 wt%) and the smallest mean particle size (29.6 µm), making it the best candidate for photocatalysis among the MSTiR materials.

### 3.2. Photocatalytic Degradation of CBZ in the Presence of MSTiR%

#### 3.2.1. Evaluation of the Photocatalytic Activity of the MSTiR% Materials

**Figure 9:**
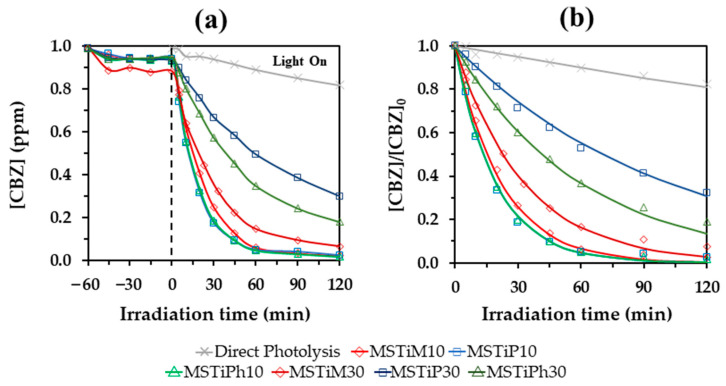
(**a**) Degradation curves of 1 ppm of CBZ using a dose of 0.5 g L^−1^ of MSTiR% materials and irradiating 2 h with 275 nm LEDs, and (**b**) First-order kinetic fittings of the degradation curves.

The photoactivity of the MSRTiR% materials was evaluated by photodegrading a 1 mg L^−1^ aqueous CBZ solution under irradiation with a 275 nm LED system for 2 h (Figure 9). Prior to irradiation, the materials were placed in the CBZ solutions and magnetically stirred during 1 h in the dark to determine if a significant amount of CBZ was adsorbed. No difference in pH was observed before and after the irradiation, remaining equal to that of the ultrapure water (pH = 6).

The degradation curves in Figure 9 show that although MSTiM30 adsorbed 11% of CBZ, the rest of the materials only adsorbed ~5%, thus it can be stated that adsorption is not significative in the removal of CBZ. According to Hunter et al. 2012, in a polar solvent such as water, CBZ forms weak dimers through aromatic interactions, exposing the amide groups to the solvent or to interaction with polar functional groups on the surface of the materials [15]. Therefore, the low CBZ adsorption rate of MSTiR% materials may be due to the low affinity between the molecule and the surface chemistry of the materials, since due to the calcination, they lack Si–OH groups that can interact or form hydrogen bonds with the amide group of CBZ.

Regarding photodegradation, direct photolysis achieved a 13% removal of CBZ after 2 h of irradiation, indicating that this compound is quite persistent. MSTiR10 materials achieved almost identical removal rates (~92.5%), while MSTiR30 materials produced lower rates: 81.41%, 76.51%, and 61.78% for MSTiM30, MSTiP30, and MSTiPh30, respectively. This was expected, since the characterization results indicated that MSTiR10 materials possess a higher amount of crystalline TiO_2_ nanoparticles and a higher volume of mesopores than their MSTiR30 analogs.

#### 3.2.2. Influence of the Catalyst Dose and Initial pH, and Reusability of MSTiR% Materials

**Figure 10:**
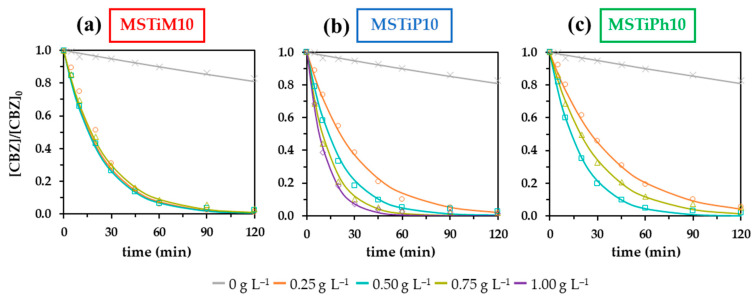
First-order kinetic fittings of the degradation curves of (**a**) MSTiM10; (**b**) MSTiP10; and (**c**) MSTiPh10.

To determine which of the MSTiR10 materials achieves the best photocatalytic performance, the effect of the catalysis dose was explored using 0.25; 0.5 and 0.75 g L^−1^. Figure 10 shows the First-order kinetic adjustment of the degradation curves obtained using different MSTiR10 catalyst doses, and Table S3 indicates the amount of CBZ removed, the calculated apparent First-order kinetic constant (*K*_app_) and the half-life (*t*_1/2_) for each MSTiR% material.

Figure 10 and Table S3 illustrate that the performance of the MSTiM10 material is practically independent of the catalyst dose, while when MSTiP10 was used, the *K*_app_ increased steadily with the dose even with 1 g L^−1^. On the other hand, the performance of the MSTiPh10 catalyst was more inconsistent, as the degradation rate increased with catalyst dose from 0.25 to 0.5g L^−1^, but then decreased when a dose of 0.75 g L^−1^ was used, probably due to the scattering of a significant percentage of the incident irradiation by the catalyst particles (screening effect). Therefore, MSTiP10 yielded the best performance in the photodegradation of CBZ (*K*_app_ = 0.0877 min^−1^ in Table S3), apparently due to its higher amount of titanium (1.36 wt% by EDX), higher crystallinity, and smaller mean particle size (29.6 µm).

**Figure 11:**
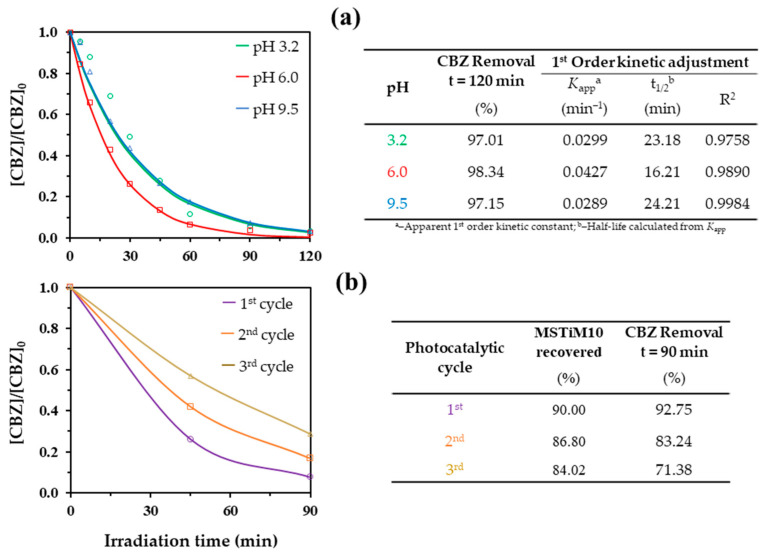
(**a**) First-order kinetic fittings of the degradation curves obtained using MSTiM10 at pH 3.2, 6.0 and 9.5; and (**b**) reuse of MSTiM10 in three consecutive photodegradation reactions. Studies were carried out using 0.5 g L^−1^ of catalyst and 1 mg L^−1^ of CBZ.

The influence of the initial pH on the photodegradation of CBZ was also evaluated. Figure 11a depicts the First-order kinetic adjustment of the degradation curves obtained using 0.5 g L^−1^ of MSTiM10 at pH 3.2, 6 and 9.5.

The table included in Figure 11a indicates that the photocatalytic degradation of CBZ at acid and basic pHs gives lower performances compared to the non-controlled pH reaction (pH 6.0), with the *K*_app_ calculated from the reaction at pH = 3.2 being practically identical to that calculated from the reaction at pH = 9.5 (0.0299 and 0.0289 min^−1^, respectively). At basic pH, the lower degradation rates can be explained by the reaction of hydroxyl anions with hydroxyl radicals to produce an oxide radical anion (O^•−^) and a water molecule [27], resulting in a lower amount of OH^•^ available to degrade CBZ. The results obtained at acid pH can be explained by H_2_O_2_. The presence of this molecule in the medium is ephemeral since although it is usually generated by the combination of two HO^•^ radicals [27], it is split into two HO^•^ radicals by the incident UVC irradiation, maintaining the contribution of HO^•^ radicals participating in the degradation of CBZ. However, in an acidic medium, H^+^ can react with the H_2_O_2_ producing two water molecules and thus reducing the amount of OH^•^ that degrades CBZ.

The reusability of the materials was studied by performing three consecutive photodegradation reactions of 1 mg L^−1^ of CBZ using 0.5 g L^−1^ of MSTiM10 at pH 6.0. A large amount of catalyst (90%, ~87% and 84%) was easily recovered by vacuum filtration after each consecutive cycle. Figure 11b shows the degradation curves obtained in the three consecutive cycles. MSTiM10 achieved removal rates of ~93%, ~83% and ~71% at 90 min of irradiation in the first, second and third cycles, respectively, indicating that the catalyst losses approximately 10% of its effectiveness with each consecutive reuse. This lower performance with consecutive use may be caused by the adsorption of CBZ molecules and their transformation products, which block the active sites of the catalyst; nevertheless, these results indicate that despite the loss of performance, the materials can still be reused in at least 3 cycles.

#### 3.2.3. Identification and Evolution of CBZ and Its Transformation Products (TPs), and Phytotoxicity Tests

The identification of the CBZ transformation products (TPs) was performed by analyzing aliquots of the photodegradation experiments, collected at t = 0, 30, 60 and 90 min of the photoperiod, in an LC-QTOF equipment. Aliquots were subtracted from 1 mg L^−1^ CBZ photodegradation experiments performed using 0.5 g L^−1^ of MSTiM10 and three different pH = 3.2, 6.0 and 9.5, obtaining the Mass spectra shown in Figures S17–S19, S20–S23 and S24–S27, respectively.

The twelve TPs tentatively identified in the analyzed water samples are listed in Table S4, where the [M + H]^+^ peak at 4.47 min was identified as the CBZ compound (C_15_H_12_N_2_O) with a *m*/*z* of 237.1021. The identified TPs listed in Table S4 were studied based on their molecular formula and classified according to the study of Martínez-Escudero et al., in which they employed LC-MS/MS to determine the fragment masses of each species and accurately identify the CBZ TPs generated during the degradation of 10 mg L^−1^ of CBZ using TiO_2_ as a photocatalyst and UV LEDs as the irradiation source [28]. Peaks holding [M + H]^+^ with *m*/*z* between 269.0922 and 269.0924 for the calculated elemental composition C_15_H_12_N_2_O_3_ were found between 3.36 and 3.80 min. The suggested elemental formula might correspond to a hydroxylated derivative of N-amino-carbonylacridine-9-carboxaldehyde (TP-6), and to the hydroxylation of oxcarbazepine (TP-5). The peaks detected for the protonated molecular ions of m/z between 253.0969 and 253.0972 (with a retention time between 3.58 and 4.08 min) were compatible with the elemental composition C_15_H_12_N_2_O_2_. According to the aforementioned literature, this molecular formula may correspond to different TPs derived from the oxidized form of CBZ. These TPs correspond to CBZ-10,11-epoxide (TP-1), 2-hydroxy-CBZ (TP-2), 3-hydroxy-CBZ (TP-3), and oxcarbazepine (TP-4). Two protonated molecular ions of *m*/*z* 267.0765 and 267.0766 detected at 3.64 and 3.74 min matched the elemental composition of C_15_H_10_N_2_O_3_. Based on this molecular formula, we can tentatively identify 11-keto oxcarbazepine (TP-10) and the fragmentation of 1-(2-benzaldehyde)-(1H,3H)-quinazoline-2,4-dione (TP-11). The chromatogram also displayed two peaks at [M + H]^+^ with *m*/*z* between 271.1077 and 271.1079 detected at retention times of 3.03 and 3.60 min. The predicted accurate mass of the protonated species might correspond to the composition C_15_H_14_N_2_O_3_ suggesting a structure consistent with the addition of two hydroxyl groups to the CBZ molecule, which aligns with the tentative assignment of 10,11-dihydrodiol-CBZ (TP-7/TP-8). A peak detected at 3.66 min provided a protonated molecular ion with *m*/*z* 287.1027. Based on theoretically accurate mass measurements revealing a molecular formula of C_15_H_14_N_2_O_4_, the structure can be proposed as a trihydroxylated form of CBZ (TP-9). The protonated molecular ion of *m*/*z* 251.0821 observed at 2.29 min indicates an elemental composition of C_15_H_10_N_2_O_2_ which can be assigned to the compound 1-(2-benzaldehyde)-4-hydro-(1H,3H)-quinazoline-2-one (TP-12). Establishing a classification, the detected CBZ TPs can be classified as acridine derivatives (TP-6), oxidized CBZ derivatives (TP-1, TP-2, TP-3, TP-4, TP-5, TP-7, TP-8, TP-9, TP-10), and oxidized CBZ derivatives with structural rearrangement (TP-11, TP-12).

**Figure 12:**
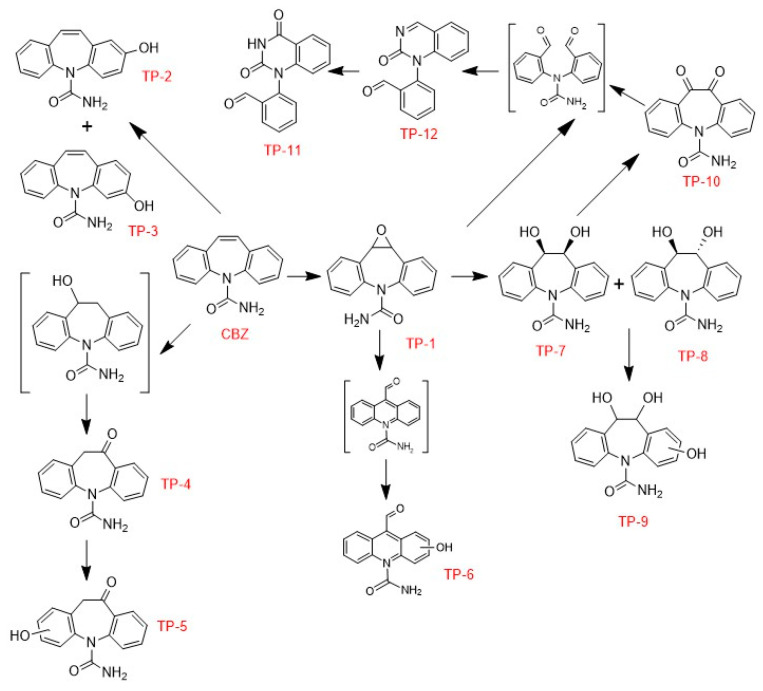
Proposed photocatalytic degradation pathway of CBZ in water using MSTiR% and 275 nm LEDS as the photocatalyst and irradiation source, respectively.

Based on the results obtained, Figure 12 shows a possible photolytic degradation pathway of CBZ that encompasses several steps.

One of the processes that can take place is the hydroxylation of an aromatic ring of CBZ by HO^•^ radicals, giving rise to the formation of hydroxylated derivatives, such as 2-hydroxy-CBZ (TP-2) and 3-hydroxy-CBZ (TP-3) [29]. On the other hand, the HO^•^ radicals could also react with the alkene double bond within the central heterocyclic ring of CBZ, leading to the formation of hydroxylated products, specifically CBZ-10,11-epoxide (TP-1) and 10,11-dihydro-10-hydroxyl-CBZ [30]. However, in this work, no peak was detected at [M + H]^+^ with *m*/*z* 255 corresponding to the compound 10,11-dihydro-10-hydroxyl-CBZ, with molecular formula C_15_H_14_N_2_O_2_. Martínez-Escudero et al. suggested that the formation of oxcarbazepine (TP-4) could be due to the oxidation of this undetected intermediate [28]. Oxcarbazepine (TP-4) was further hydroxylated to hydroxyl–ketone structures (TP-5) [31]. Compound CBZ-10,11-epoxide (TP-1) might experience further hydroxylation, resulting in an intermediate featuring two carbonyl functionalities, which is capable of generating the compound 1-(2-benzaldehyde)-4-hydro-(1H,3H)-quinazoline-2-one (TP-12) and the fragmentation of 1-(2-benzaldehyde)-(1H,3H)-quinazoline-2,4-dione (TP-11) [32]. Furthermore, CBZ-10,11-epoxide (TP-1) might also be subject to hydrolysis, yielding 10,11-dihydrodiol-CBZ (TP-7/TP-8), which could then undergo further hydroxylation to produce a trihydroxylated derivative of CBZ (TP-9) [33]. Dehydrogenation of 10,11-dihydrodiol- CBZ (TP-7/TP-8) might generate 11-keto oxcarbazepine (TP-10), which can suffer heterocyclic ring-opening that would also lead to intermediates and subsequent 1-(2-benzaldehyde)-4-hydro-(1H,3H)-quinazoline-2-one (TP-12) and the fragmentation of 1-(2-benzaldehyde)-(1H,3H)-quinazoline-2,4-dione (TP-11) [34]. Finally, the formation of the corresponding hydroxyl derivatives (TP-6) proceeds from an intermediate structure generated from the compound CBZ-10,11-epoxide (TP-1) [34].

**Figure 13:**
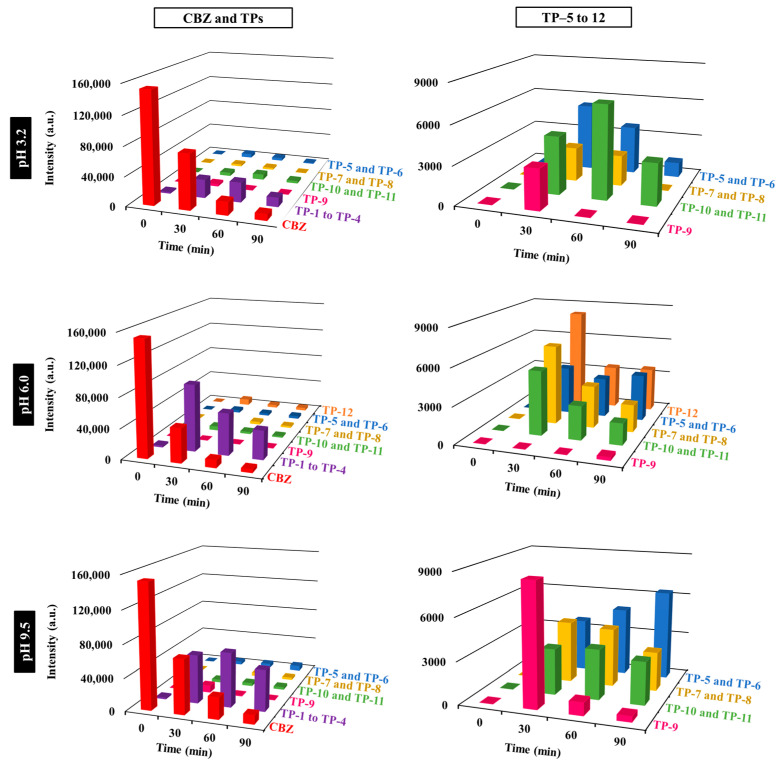
Relative abundance of the transformation products (TPs) with time, originated during the photodegradation of 1 mg L^−1^ CBZ using the 0.5 g L^−1^ of the MSTiM10 photocatalyst and a 275 nm LEDs irradiation source and three different levels of pH = 3.2, 6.0 and 9.5.

To elucidate the reaction mechanisms underlying the photolytic degradation of CBZ, the kinetic profiles of its principal transformation products were investigated during the course of the irradiation experiment. As evidenced in Figure 13, CBZ degradation was better at pH 6 than at acidic or basic pH, reaching almost 100% of total degradation at 90 min, as also confirmed by the results obtained from the photodegradation kinetics by HPLC (Figure 11a). Degradation at pH 3.2 was slightly better than at pH 9.5. The first column of graphs in Figure 13 shows that TP-1, TP-2, TP-3 and TP-4 were the most abundant transformation products for the three pH levels. Nevertheless, the absorption of these TPs differs depending on the pH, with pH 3.2 and 9.5 having the lowest and highest absorption, respectively, as expected by the higher concentration of HO^−^ anions in the latter. The second column of Figure 13 depicts the absorption of the other transformation products, whose relative concentrations at the end of the photodegradation process (t = 90 min) are severely affected by the pH reaction media: (i) with an acidic media, a small proportion of TP-5, TP-6; TP-10 and TP-11 were detected; (ii) under non-controlled pH reaction, all the transformation products from TP-5 to TP-12 were detected with the exception of TP-9, and the absorption of TP-12 and that of the TPs with C_15_H_12_N_2_O_3_ formula (TP-5 and TP-6) doubled that of the TPs with C_15_H_14_N_2_O_3_ (TP-7 and TP-8) and C_15_H_10_N_2_O_3_ (TP-10 and TP-11) formulas; and (iii) under basic media, the absorption of the TPs of C_15_H_12_N_2_O_3_ (TP-5 and TP-6) was considerably higher to that of the rest of the transformation products.

**Figure 14:**
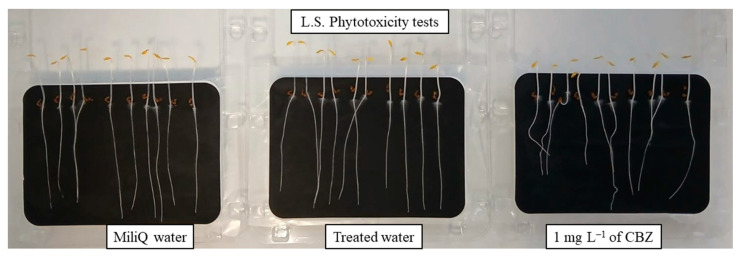
Phytotoxicity tests on *Lepidium sativum* (L.S.) seeds watered with miliQ water, 1 mg L^−1^ CBZ solution and water treated with 0.5 g L^−1^ of MSTiM10 and 275 nm LEDs.

Finally, to evaluate the harmful effect of 1 mg L^−1^ CBZ solutions after photocatalysis (at pH 6.0), and therefore the toxicity of the resulting TPs, phytotoxicity tests were performed using seeds of three fast-growing plant species. Three batches of ten seeds of each species were watered with miliQ water, the 1 mg L^−1^ CBZ solution, and treated water. Figure 14 shows germinated L.S. seeds after three days of growth at 25 °C and in the dark. Germinated S.A. and S.S. seeds are shown in Figure S28.

As can be seen in the photograph of Figure 14, L.S. the roots and stems grew more when watered with treated water than with the 1 mg L^−1^ CBZ solution, indicating that for L.S., the TPs formed during the photodegradation are less toxic than CBZ. Table 6 includes the measured length of the roots and stems of the three species of plants.

As expected, all three plants species had longer roots and stems when watered with mili Q water. Seeds watered with the treated solution showed greater growth than those watered with the CBZ solution, with L.S. species having a mean root + stem lengths 2 cm longer. Therefore, it can be concluded that the solution photocatalytic treated with MSTiM10 has a lower toxicity than the untreated solution.

#### 3.2.4. Comparison with Other CBZ Photocatalysts and TiO_2_-Supported in SiO_2_ Materials

To evaluate the effectiveness of the MSTiP10 catalyst, Table S5 illustrates the current state-of-the-art of CBZ photodegradation using different catalysts, detailing the applied experimental conditions and their degradation, while Table S6 covers the use SiO_2_-supported TiO_2_ materials in the photocatalytic degradation of the contaminants.

Table S5 indicates that most studies used a higher concentration of CBZ than this work. However, as reported by Fernández et al., the concentration of CBZ in WWTP effluents in northern Spain ranges between 30 and 400 ng L^−1^, levels similar to those of other studies worldwide [35]. Therefore, high concentrations are not necessary to evaluate the CBZ photodegradation performance of a catalyst, and the criterion followed selected 0.25–1 mg L^−1^ as the CBZ concentration, as in this work, the aim was to obtain reliable kinetics parameters through HPLC. Another topic worth highlighting is that only three of the references listed in the table used irradiation sources aligned with the principles of green chemistry, such as natural light or LEDs, as is the case of this work. Compared with the references listed in Table S5, MSiPh10 showed a removal efficiency of 97.57% of 1 g L^−1^ of CBZ after 1 h of irradiation, which is very similar to that reported by Meng et al., who for the same irradiation time achieved a removal rate of ~95% also using 1 g L^−1^ of catalyst (g-C_3_N_4_/TiO_2_ composite) and an irradiation source with a similar wavelength (285 nm) [36]. Furthermore, the kinetic rate in the photodegradation of 1 mg L^−1^ of CBZ using 1 g L^−1^ of catalyst was higher for MSTiPh10 (0.0877 min^−1^) than the reported when a mesoporous Fe_3_O_4_-modified Al-doped ZnO was used (0.076 min^−1^) [37]. However, the potassium and oxygen co-doped g-C_3_N_4_ synthesized by Wang et al. yielded higher efficiency than MSTiPh10, as it completely removed 1 g L^−1^ of CBZ using a lower amount of catalyst (0.4 g L^−1^) and requiring only 30 min of irradiation [38].

Finally, compared to the recently reported TiO_2_-SiO_2_ materials described in Table S6, MSTiR% exhibit an amorphous nature, contrasting with their highly ordered porosity, and a considerably lower amount of Ti (1.36 wt% of Mstiph10 vs. 15–30 wt%). For example, Zhou Y. et al. reported Si-doped hollow hemispherical anatase with a hierarchically porous structure that with only a 0.1 g L^−1^ catalyst dose completely removed 10 mg L^−1^ of CBZ in 60 min using simulated solar light [39]. However, this high performance was achieved through peroxymonosulfate activation, while MSTiR% materials do not require the use of oxidants and contain considerably less titanium.

## 4. Conclusions

Three series of materials consisting of anatase supported on mesoporous silica matrices were prepared using different organotriethoxysilanes, RTEOS (where R = M, methyl; P, propyl; or Ph, phenyl) with a molar content of 10 and 30% with respect to tetraethoxysilane (TEOS). A hydrothermal approach originally designed for the preparation of titanosilicalites was followed, where in this work tetrapropyl ammonium bromide (TPABr) was used as a surfactant instead of tetrapropyl ammonium hydroxide (TPAOH). The materials were thoroughly characterized using a plethora of techniques. For instance, the XRD pattern and the FTIR spectra of the materials confirmed that the use of TPABr prevented the formation of the crystalline modernite-inverted framework (MFI) of titanosilicalites, promoting the formation of anatase particles that are supported on amorphous SiO_2_. Furthermore, the degree of crystallinity of MSTiR30 materials was lower than that of their respective MSTiR10 analogs, indicating that an increase in the molar percentage of RTEOS hinders the formation of ordered structures during the hydrothermal synthesis. MSTiM10, the material synthesized using 10% of the less bulky organic precursors (MTEOS), showed the highest degree of crystallinity among all samples, as well as a higher UV-vis absorption in the range of 260–380 nm, corresponding to the TiO_6_ photoactive surface species. Textural characterization acquiring a N_2_ adsorption isotherm at −196 °C, demonstrated that (i) the bulkier the RTEOS precursor, the higher the *a*_BET_ and *V*_meso_ of the MSTiR% material; and (ii) while PhTEOS promotes the formation of micropores, PTEOS induces the formation of wider mesopores. This is consistent with the relative abundance of titanium determined at the surface of the materials by XPS, and in bulk by FE-SEM EDX, as MSTiPh10 was found to show significantly higher surface titanium content. Regarding the photodegradation of CBZ (1 mg L^−1^), all materials were able to degrade a significative proportion of the contaminant after 2 h of irradiation using a LED system (λ_Max_ = 275 nm) and a catalyst dose of 0.5 g L^−1^, with the three MSTiR10 materials achieving a removal rate of ~92.5%. The influence of the catalyst dose and pH was studied, finding that the performance of MSTiP10 increased with doses up to 1 g L^−1^ and that the use of acid and basic media (pH = 3.2 and 9.5, respectively) reported lower apparent First-order kinetic constants (*K*_app_) than in reactions without controlled pH (pH = 6.0). Therefore, it was concluded that the better performance of MSTiP10 is related to the higher amount of Ti in the bulk (1.36 wt%), probably due to its wider porosity that allows a better distribution of the anatase particles on the SiO_2_ surface, which favor its adsorption of CBZ and the generation of ROS. Finally, Tauc-plot analyses of the UV–Vis diffuse reflectance spectra and valence band XPS spectra were performed to elucidate the electronic configuration of the MSTiR% materials. All materials have bandgaps of ~3.3 eV and a valence band maximum and conduction band minimum energy positions, suggesting that HO^•^ radicals and presumably electrons, since the generation of O_2_^•−^ radicals is not favored, are the main species that degrade CBZ molecules. CBZ transformation products were analyzed by LC-QTOF, and traces of 12 transformation products originating from the hydroxylation and oxidation of CBZ were found. A photodegradation pathway was proposed based on the results and literature reviewed, and phytotoxicity tests demonstrated that the treated solutions were less toxic than the CBZ initial solution. In summary, this study has served to prove how focusing on textural properties can also have a critical impact on the performance of a photodegradation reaction, rather than only addressing the amount of photoactive Ti species, paving the way for the future design of SiO_2_-supported TiO_2_ nanoparticle-based materials with enhanced textural properties.

## Figures and Tables

**Table 1:** Identified anatase diffraction maxima and determined structural parameters of the MSTiR% materials.

MSTiR%	(h, k, l)	2θ	*d*_(h, k, l)_^a^	*D*_(101)_^b^	Degree of Crystallinity ^c^
		(°)	(nm)	(nm)	(%)
**MSTiM10**	(101)	25.48	0.350	8.4	13.2
	(200)	48.23	0.189		
	(105)	54.46	0.168		
	(211)	55.27	0.166		
**MSTiP10**	(101)	25.37	0.351	9.1	11.4
	(200)	48.05	0.189		
	(105)	54.39	0.169		
	(211)	55.08	0.167		
**MSTiPh10**	(101)	25.47	0.350	9.0	9.2
	(200)	48.28	0.189		
**MSTiM30**	(101)	25.53	0.349	8.8	11.1
	(200)	48.24	0.189		
	(105)	54.52	0.168		
	(211)	55.18	0.166		
**MSTiP30**	(101)	25.39	0.351	9.1	8.1
	(200)	48.05	0.189		
**MSTiPh30**	(101)	25.34	0.351	10.7	6.9
	(200)	48.13	0.189		

^a-^Interplanar space calculated using the Bragg equation; ^b-^crystallite size calculated from the Debye–Scherrer equation; ^c-^degree of crystallinity.

**Table 2:** Relative surface atomic composition (wt%) of the MSTiR% materials determined by XPS.

MSTiR%	C1s	O1s	Si2p	Ti2p	
	Weight (%)				
**MSTiM10**	3.75	54.52	41.27	0.45	
**MSTiP10**	4.28	54.51	40.63	0.57	
**MSTiPh10**	3.78	55.20	40.23	0.77	
**MSTiM30**	5.75	53.32	40.57	0.35	
**MSTiP30**	4.52	54.69	40.53	0.25	
**MSTiPh30**	4.50	54.61	40.43	0.45	

**Table 3:** Tauc-plot bandgap energy (*E*_g_), valence band maximum and conduction band minimum edge potential (*E*_VBM_ and *E*_CBM_, respectively) of the MSTiR% materials relative to the vacuum level (*V*_Vacuum_) and the Normal Hydrogen Electrode at 25 °C and pH = 0 (*V*_NHE_).

MSTiR%	Bandgap Energy (*E*_g_)	Valence Band Maximum Edge Potential (*E*_VBM_)		Conduction Band Minimum Edge Potential (*E*_CBM_)	
	(*eV*)	(*V*_Vacuum_)	(*V*_NHE_)	(*V*_Vacuum_)	(*V*_NHE_)
**MSTiM10**	3.33	−9.09	4.65	−5.76	1.32
**MSTiP10**	3.33	−8.88	4.44	−5.55	1.11
**MSTiPh10**	3.33	−9.05	4.61	−5.72	1.28
**MSTiM30**	3.34	−9.05	4.61	−5.71	1.27
**MSTiP30**	3.31	−9.28	4.84	−5.97	1.53
**MSTiPh30**	3.32	−9.32	4.88	−6.00	1.56

**Table 4:** Textural parameters of the MSTiR% materials.

Material	*a*_BET_	*a*_DR_	*V*_micro_^a^	*V*_meso_^b^	*V*_macro_^c^	*V*_total_^d^	BJH APS ^e^	*E*_c_^f^
	(m^2^ g^−1^)		(cm^3^ g^−1^)				(nm)	(KJ mol^−1^)
**MSTiM10**	608	668	0.24	0.60	0.14	0.98	6.83	12.19
**MSTiP10**	810	902	0.32	0.59	0.11	1.02	6.12	12.88
**MSTiPh10**	1047	1191	0.42	0.67	0.20	1.28	6.31	12.57
**MSTiM30**	753	837	0.30	0.60	0.12	1.02	6.02	11.83
**MSTiP30**	774	879	0.31	0.47	0.35	1.13	7.91	13.28
**MSTiPh30**	921	1062	0.38	0.34	0.10	0.82	5.26	13.35

^a-^Micropore volume obtained from Dubinin–Raduskevich; ^b-^mesopore volume obtained subtracting the micro and macropore volume to total pore volume; ^c-^macropore volume obtained from 0.8 ≥ *p*/*p*° ≤ 0.95; ^d-^Total pore volume obtained from isotherm at *p*/*p*° = 0.95; ^e-^BJH average pore size obtained from desorption branch; ^f-^characteristic energy from Dubinin–Raduskevich.

**Table 5:** Estimated weight percentage of C, O, Si, and Ti in the bulk MSTiR% materials by EDX sum spectra; mean particle size (MPS) and standard deviation (S) based on the micrographs of Figure 7, Figures S10 and S11; and ratio of Ti on the surface and in the bulk (Ti_XPS_/Ti_EDX_).

MSTiR%	C	O	Si	Ti	MPS	S	Ti_XPS_/Ti_EDX_
	Weight (%)				(µm)		
**MSTiM10**	24.47	46.59	27.93	1.01	46.0	41.8	0.45
**MSTiP10**	21.63	46.04	30.98	1.36	29.6	26.7	0.42
**MSTiPh10**	34.41	43.30	21.18	1.10	34.8	26.6	0.70
**MSTiM30**	31.39	42.05	26.03	0.52	37.4	31.3	0.67
**MSTiP30**	26.02	45.07	28.21	0.70	44.9	35.0	0.36
**MSTiPh30**	26.27	43.14	29.41	1.19	38.4	25.9	0.38

**Table 6:** Phytotoxicity test results of the photocatalytically treated 1 mg L^−1^ CBZ solution (0.5 g L^−1^ MSTiM10 at pH = 6.0) using seeds of three different plants: *Sinapis alba* (S.A.), *Sorghum saccharate* (S.S.), and *Lepidium sativum* (L.S.).

Plants	Used Water	Germinated Seeds	Root Average Length	Stem Average Length	Root + Stem Mean Length
			(cm)		
S.A.	Mili Q	5/10	7.4 ± 0.4	1.7 ± 0.7	9.1 ± 0.4
	CBZ solution	8/10	4.0 ± 0.8	1.7 ± 0.9	5.8 ± 1.0
	Treated	7/10	4.0 ± 1.0	2.0 ± 0.3	6.0 ± 1.3
S.S.	Mili Q	10/10	4.0 ± 0.5	0.8 ± 0.3	4.8 ± 0.8
	CBZ solution	9/10	3.3 ± 0.5	0.4 ± 0.3	3.7 ± 0.7
	Treated	9/10	3.7 ± 0.5	0.8 ± 0.3	4.5 ± 0.9
L.S.	Mili Q	10/10	6.5 ± 0.1	3.0 ± 0.1	9.5 ± 0.2
	CBZ solution	10/10	5.1 ± 0.4	2.3 ± 0.1	7.5 ± 0.5
	Treated	10/10	6.6 ± 0.2	2.9 ± 0.1	9.5 ± 0.1

## Data Availability

The data presented in this study are available from the corresponding authors upon reasonable request.

## Supplementary Materials

The following supporting information can be downloaded at: https://www.mdpi.com/article/10.3390/nano15191533/s1, **Table S1:** Chemical and physical properties of the carbamazepine molecule; **Figure S1:** (a) LED system used in the photodegradation experiments; and (b) UV-vis emission spectrum of the LEDs (Luminus, mod XFM-5050-UV-D130-FE270-00) provided by the manufacturer; **Figure S2:** Zoomed XRD patterns of the (a) MSTiR10 and (b) MSTiR30 materials in the 35–40°, 45–50° and 50–60° ranges; **Table S2:** Identified anatase diffraction maxima, recorded intensity (*I*), Full Width at Half maximum (*FWHM*) and integrated Area (*A*); **Figure S3:** Diffraction patterns of the TiO_2_ Degussa P25 Aeroxide to illustrate the more intense diffraction maxima belonging to anatase and rutile in the 10–60° range; **Figure S4:** FTIR spectra of (a) MSTiR10 and (b) MSTiR30 in the 4000–2750 and 1600 400 cm^−1^ spectral range; **Figure S5:** Tauc-plot analyses of the UV-Vis reflectance diffuse spectra of the (a) MSTiR10 and (b) MSTiR30 materials; **Figure S6:** XPS spectra of the (a) MSTiR10 and (b) MSTiR30 materials and peaks assignation; **Figure S7:** Linear fit of the MSTiP10, MSTiPh10, MSTiR30 spectra to determine their valence band maximum edge potential (*E*_VBM_); **Figure S8:** Bandgaps and valence and conduction bands (blue and red, respectively) of the MSTiM10 material and of pure anatase, and the redox potentials for the generation of hydroxyl (HO^•^) and superoxide (O_2_^•−^) radicals; **Figure S9:** Isotherms of the materials prepared using TPAOH instead of TPBr: (a) titanosilicalites prepared with 10% of RTEOS; and (b) amorphous materials with no significative micro or narrow mesoporosity prepared with 30% of RTEOS; **Figure S10:** FE-SEM micrographs of the MSTiR30 materials obtained at magnifications of 6.00 KX and 48.00 KX (particles in red circles in 6.00 KX micrographs). MPS = mean particle size; S = standard deviation; **Figure S11:** FE-SEM micrographs of the MSTiR% materials obtained at magnifications of 1.00 KX. MPS = mean particle size; S = standard deviation; **Figure S12:** EDX mapping analysis of the MSTiP10 6.0 KX micrograph and relative abundances of C, O, Si and Ti calculated from the acquired spectra of different particles and from their sum spectra; **Figure S13:** EDX mapping analysis of the MSTiPh10 6.0 KX micrograph and relative abundances of C, O, Si and Ti calculated from the acquired spectra of different particles and from their sum spectra; **Figure S14:** EDX mapping analysis of the MSTiM30 6.0 KX micrograph and relative abundances of C, O, Si and Ti calculated from the acquired spectra of different particles and from their sum spectra; **Figure S15:** EDX mapping analysis of the MSTiP30 6.0 KX micrograph and relative abundances of C, O, Si and Ti calculated from the acquired spectra of different particles and from their sum spectra; **Figure S16:** EDX mapping analysis of the MSTiPh30 6.0 KX micrograph and relative abundances of C, O, Si and Ti calculated from the acquired spectra of different particles and from their sum spectra; **Table S3:** Removal of CBZ, First-order kinetic constant (*K*_app_), and half-life (*t*_1/2_) for each catalyst dose of the MSTiR% materials; **Figure S17:** HPLC chromatograms and Mass spectra of carbamazepine (CBZ) acquired from aliquots removed at t = 0, 30, 60, and 90 min from the photodegradation reaction of 1 mg L^−1^ of CBZ using 0.5 g L^−1^ of MSTiM10 at pH = 3.2; **Figure S18:** Mass spectra showing the detected transformation products TP-1 to TP-6 in the aliquots removed at t = 0, 30, 60, and 90 min from the photodegradation reaction of 1 mg L^−1^ of CBZ using 0.5 g L^−1^ of MSTiM10 at pH = 3.2; **Figure S19:** Mass spectra showing the detected transformation products TP-7 to TP-11 in the aliquots removed at t = 0, 30, 60, and 90 min from the photodegradation reaction of 1 mg L^−1^ of CBZ using 0.5 g L^−1^ of MSTiM10 at pH = 3.2; **Figure S20:** HPLC chromatograms and Mass spectra of carbamazepine (CBZ) acquired from aliquots removed at t = 0, 30, 60, and 90 min from the photodegradation reaction of 1 mg L^−1^ of CBZ using 0.5 g L^−1^ of MSTiM10 at pH = 6.0; **Figure S21:** Mass spectra showing the detected transformation products TP-1 to TP-6 in the aliquots removed at t = 0, 30, 60, and 90 min from the photodegradation reaction of 1 mg L^−1^ of CBZ using 0.5 g L^−1^ of MSTiM10 at pH = 6.0; **Figure S22:** Mass spectra showing the detected transformation products TP-7 to TP-9 in the aliquots removed at t = 0, 30, 60, and 90 min from the photodegradation reaction of 1 mg L^−1^ of CBZ using 0.5 g L^−1^ of MSTiM10 at pH = 6.0; **Figure S23:** Mass spectra showing the detected transformation products TP-10 and TP-11, and HPLC chromatograms showing the detected TP-12 in the aliquots removed at t = 0, 30, 60, and 90 min from the photodegradation reaction of 1 mg L^−1^ of CBZ using 0.5 g L^−1^ of MSTiM10 at pH = 6.0; **Figure S24:** HPLC chromatograms and Mass spectra of carbamazepine (CBZ) acquired from aliquots removed at t = 0, 30, 60, and 90 min from the photodegradation reaction of 1 mg L^−1^ of CBZ using 0.5 g L^−1^ of MSTiM10 at pH = 9.5; **Figure S25:** Mass spectra showing the detected transformation products TP-1 to TP-6 in the aliquots removed at t = 0, 30, 60, and 90 min from the photodegradation reaction of 1 mg L^−1^ of CBZ using 0.5 g L^−1^ of MSTiM10 at pH = 9.5; **Figure S26:** Mass spectra showing the detected transformation products TP-7 to TP-9 in the aliquots removed at t = 0, 30, 60, and 90 min from the photodegradation reaction of 1 mg L^−1^ of CBZ using 0.5 g L^−1^ of MSTiM10 at pH = 9.5; **Figure S27:** Mass spectra showing the detected transformation products TP-10 and TP-11 in the aliquots removed at t = 0, 30, 60, and 90 min from the photodegradation reaction of 1 mg L^−1^ of CBZ using 0.5 g L^−1^ of MSTiM10 at pH = 9.5; **Table S4**: Tentative assignation of the transformation products of CBZ analyzed by LC-QTOF with their detected mass/charge (*m*/*z*) and retention times (*t*_r_); **Figure S28**: Phytotoxicity tests on (a) *Sinapis alba* (S.A.) and (b) *Sorghum saccharate* (S.S.) seeds watered with miliQ water, 1 mg L^−1^ CBZ solution and water treated with 0.5 g L^−1^ of MSTiM10 and 275 nm LEDs; **Table S5:** Performance of the catalyst on the photodegradation of CBZ; **Table S6:** Recent published studies on TiO_2_-based materials supported on SiO_2_ for the removal of water contaminants; references [40,41,42,43,44,45,46,47,48,49,50,51,52,53,54,55,56,57,58,59,60,61,62,63,64,65,66].
