# Supplementary material for: Photocatalytic Enhancement of Anatase Supported on Mesoporous Modified Silica for the Removal of Carbamazepine
Source: Nanomaterials (Basel). 2025 Oct 8;15(19):1533. doi: 10.3390/nano15191533 (PMC12526347; doi:10.3390/nano15191533)
Supplement: Supplementary file 1 [file nanomaterials-15-01533-s001.zip › nanomaterials-3818499-supplementary.pdf]

Supplementary Material

Photocatalytic enhancement of anatase supported on mesoporous modified silica for the removal of carbamazepine

Guillermo Cruz-Quesada[1], Beatriz Rosales-Reina[2], Inmaculada Velo-Gala[3], María del Pilar Fernández-Poyatos[1], Miguel A. Álvarez[1], Cristian García-Ruiz[1], María Victoria López-Ramón[1*], Julián J. Garrido[2*]

[1] Department of Inorganic and Organic Chemistry, Faculty of Experimental Science, University of Jaén (UJA), Campus Las Lagunillas, 23071 Jaén, Spain.

[2] Department of Science, Institute for Advanced Materials and Mathematics (INAMAT[2]), Public University of Navarre (UPNA), Campus Arrosadía, 31006 Pamplona, Spain

[3] Department of Inorganic Chemistry, Faculty of Farmacy, University of Granada, 18011 Granada, Spain.

*Corresponding authors: mvlro@ujaen.es (M. V. L.-R.); j.garrido@unavarra.es (J. J. G.)

Tel.: +34-953-212747 (M.V. L.-R.); +34-948-168082 (J. J. G.)

INDEX

S1. Detailed characterization methods…………………………………… 2

S2. FT-IR bands identification and discussion ………………………… 4

S3. Supplementary Figures and Tables…………………………………... 5

S4. References…………………………………………………………...... 33

S1. DETAILED CHARACTERIZATION METHODS

X-ray diffraction patterns were obtained at room temperature, using a PANalyticalEmpyrean XRD instrument (Empyrean, Almelo, The Netherlands) with copper rotating anode and graphite monochromator (at 45 kV and 40 mA) to select the CuK$\alpha_{1/2}$ wavelength of the incident beam at 0.154 nm. Measurements were performed in a stepped scan mode of 5 ≤ 2θ ≤ 60° in steps of 0.013° at a rate of 0.5 steps s^{-1}. The interplanar distances ($d_{(h, k, l)}$) were calculated from each crystalline maxima applying the Bragg equation (*Eq. S1*) [40]; the Crystallite Size ($D_{(h, k, l)}$) was calculated applying the Debye–Scherrer equation (*Eq. S2*) [41]; and the Degree of Crystallinity was calculated from the areas of the diffraction maxima (obtained by integration) and the area of the full diffractogram (*Eq. S3*).

$$d_{(h,k,l)} = \frac{n\lambda}{2\sin\theta} \qquad (Eq.\ S1)$$

$$D_{(h,k,l)} = \frac{k\lambda}{\beta\cos\theta} \qquad (Eq.\ S2)$$

$$\text{Crystallinity (\%)} = \frac{\sum A_{(h,k,l)}}{A_{Total}} \times 100 \qquad (Eq.\ S3)$$

where n is the diffraction order which is equal to 1; λ is the wavelength of the incident beam (0.154 nm); θ is the diffraction angle in radians; k is a dimensionless shape factor, which has a typical value of 0.9; β is the FWHM in radians of the maximum with the highest intensity; $A(h, k, l)$ is the area of each crystalline maximum; and A_{Total} is the area of the whole diffractogram (crystalline + amorphous phases).

Infrared spectra were obtained using a FTIR spectrometer (Jasco mod. 4700, Japan), yielding the following two spectral ranges with two different sample concentrations in order to facilitate band comparisons: (i) 4000 – 2200 cm^{-1}, recorded with 2 mg of sample dispersed in 200 mg KBr to examine in greater detail the region where hydroxyl and C–H bonds appear and (ii) 2200 – 400 cm^{-1}, recorded with 0.6 mg of sample in 200 mg KBr to avoid saturation of the Si–O–Si asymmetric stretching signa [42]. Tablets were dried overnight in an oven at 115 ºC under vacuum to minimize the amount of water adsorbed. Spectra were recorded using 25 scans and a resolution of 4 cm^{-1}.

Ultraviolet-Visible diffuse reflectance spectra (UV-Vis DRS) were acquired in the measurement range of 200–2000 nm at 25 °C. The samples' reflectance (R) was recorded in a UV-Visible-NIR Varian spectrometer (model CARY-5E) equipped with a spherical diffuse reflectance accessory, where $BaSO_4$ was used as the reflectance standard due to its 100% reflectance in the visible region. The reflectance of the samples considering an infinitely thick specimen (R_∞) was

calculated applying *Eq. S4*, and then transformed to Kubelka–Munk units using *Eq. S5* to allow the calculation of the bandgap energy applying the Tauc-plot (*Eq. S6*) [43].

$$R_\infty = \frac{R_{sample}}{R_{BaSO_4}} \quad (Eq.\ S4)$$

$$F(R_\infty) = \frac{K}{S} = \frac{(1 - R_\infty)^2}{2R_\infty} \quad (Eq.\ S5)$$

$$(F(R_\infty)h\upsilon)^{\frac{1}{r}} = \mathrm{B}(\mathrm{h}\upsilon - E_g) \quad (Eq.\ S6)$$

where K is the absorption coefficient; S is the scattering coefficient; h is the Planck's constant (4.14×10^{-15} eV s); υ is the frequency of the photons; r is a factor that depends on the nature of the materials—for the MSTiR% materials it is equal to 2 since TiO_2 is considered an indirect semiconductor; B is a constant; and E_g is the optical bandgap energy in eV.

The surface atomic composition and the valence band maximum edge potential (E_{VBM}) of the materials were determined by X-ray photoelectron spectroscopy (XPS). The spectra were recorded with a constant pass energy value of 29.35 eV, 0.125 eV step^{-1} and a beam diameter of 200 μm using a physical electronic spectrometer (PHI Versa Probe II, Physical Electronics, Minneapolis, MN, USA) with monochromatic X-ray Al Kα radiation (15 kV, 1486.6 eV), a hemispherical multichannel detector, and a dual beam charge neutralizer for analyzing the core-level signals of the elements of interest which were C1s, O1s, O2s, Si2s, Si2p and Ti2p. The position and areas of the peaks were determined using the C1s adventitious carbon peak at 284.6 eV as an internal standard. The E_{VBM} respective to the Fermi level (V_{Fermi}) was obtained from the x-axis intersection of the linear regression applied to the plot of the recorded XPS intensity vs. the Binding energy (BE) at its lowest eV range (32 to 0). The value of E_{VBM} respective to the vacuum level (V_{Vacuum}) was calculated using *Eq. S7-9*.

$$E_{VBM}\ (V_{Vacuum}) = E_{Fermi} - E_{VBM}\ (V_{Fermi}) \quad (Eq.\ S7)$$

$$\phi = E_{Vacuum\ level} - E_{Fermi} \quad (Eq.\ S8)$$

$$E_{VBM}\ (V_{Vacuum}) \approx -(\phi + E_{VBM}\ (V_{Fermi})) \quad (Eq.\ S9)$$

where E_{Fermi} is the energy of the Fermi level (eV); $E_{Vacuum\ level}$ is the energy of an electron at rest in vacuum near the surface of the semiconductor, which is ~ 0 eV; and ϕ (eV) is the work function, which is defined as the minimum energy required to remove an electron from a material at 0 K. The ϕ of pure TiO_2 (101) anatase measured at vacuum, 4.64 eV [44], was used to calculate E_{VBM} respective to the vacuum level in *Eq. S9*. Once E_{VBM} and the E_g (from the UV-vis diffuse reflectance) were determined, the conduction band minimum edge potential (E_{CBM}) was calculated using *Eq. S10*.

$$E_{CBM}\ (V_{Vacuum}) = E_{VBM}\ (V_{Vacuum}) + E_g \quad (Eq.\ S10)$$

Finally, *Eq. S11* was used to obtain the value of E_{VBM} and E_{CBM} respective to the Normal Hydrogen Electrode at 25 °C and pH = 0 (V_{NHE}).

$$E\ (V_{NHE}) = -(E\ (V_{Vacuum}) + E_{NHE\ vs\ Vacuum}) \qquad (Eq.\ S11)$$

where $E_{NHE\ vs\ Vacuum}$ is the potential of the hydrogen electrode relative to vacuum, which according to IUPAC has a value of 4.44 eV.

N_2 adsorption isotherms (−196 °C) were determined with a volumetric adsorption system (ASAP2020, Micromeritics, Norcross, GA, USA), weighing approximately 150 mg of sample into a straight-walled Pyrex glass tube followed by degassing at 200 °C for ≤ 2 h with a residual vacuum of < 0.66 Pa. Analysis time ranged from 14 to 55 h. The sample tube was covered with an isothermal jacket and immersed in a Dewar with liquid nitrogen (−196 °C). The recorded adsorption data were analyzed with the Microactive (version 4.06) software of the system, adjusting the parameters as appropriate for each model. Specific surface areas were calculated using the Brunauer–Emmett–Teller (BET) model (a_{BET}) and the Rouquerol criteria [45], and the characteristic adsorption energy ($E_c(N_2)$) were calculated using the Dubinin–Radushkevich method (DR) [46]. The Total pore volume of the samples (V_{Total}) was calculated with the amount of N_2 adsorbed at = 0.95 partial pressure (p/pº), while the other pore volumes were defined by their pore width (∅): (i) the volume of micropores (∅ ≤ 2 nm) was obtained from the DR method (V_{micro}); (ii) the volume of mesopores (2 < ∅ ≤ 50 nm) was calculated by abstracting V_{micro} and the volume of macropores (V_{macro}) from V_{Total}; (iii) the V_{macro} was obtained from the isotherm data at 0.8 > p/pº < 0.95. Liquid density of N_2 was obtained from the literature (0.808 g cm^{-3}) [47]. Mean pore sizes were further determined by applying the Barrett–Joyner–Halenda (BJH) method to the desorption curves [48], using a Kruk–Jaroniec–Sayari correction and thickness curve, and porosity distributions were estimated according to density functional theory (DFT) using SAIEUS software and applying the "carbon-N_2-77, 2D-NLDFT heterogeneous Surface" model [49].

S2. FT-IR bands identification and discussion

The characteristic bands of amorphous silica are present in the FT-IR spectra (Figure S3) of all samples: (i) 455 cm^{-1}, O–Si–O rocking (ϱ O–Si–O); (ii) 800 cm^{-1}, Si–O–Si symmetric stretching (vs. Si–O–Si); (iii) 955 cm^{-1}, Si–O symmetric stretching of the surface silanols groups (vs. Si–OH); (iv) 1090 cm^{-1}, asymmetric stretching vibration of Si–O–Si (νas Si–O–Si); (v) 3450 cm^{-1}, stretching of surface silanols (ν Si–OH); and (vi) 3660 cm^{-1}, stretching of surface silanols interacting through hydrogen bonds (ν Si–OH–H) [50]. The lack of bands belonging to the MFI Zeolite (1230 and 550 cm^{-1}) [51] is consistent with the XRD patterns of the materials; in addition, the ν_{as} Si–O–Si band is located at 1090 rather than 1110 cm^{-1} as is usually observed in titanosilicalites. No C–H stretching

bands were observed in the 3000 – 2850 cm^{-1} spectral range, indicating the removal of the organic moieties from the hybrid precursors during the calcination at 550 °C. Nevertheless, the methyl group of the MTEOS precursors is still present in the calcinated MSTiM10 and MSTiM30 materials, whose spectra show the band associated with the C–H wagging vibration mode (ω C–H) highlighted at 1278 cm^{-1} [52] in Figure S3.

S3. Supplementary Figures and Tables

Table S1. Chemical and physical properties of the carbamazepine molecule [53–55].

**CBZ Molecule ($C_{15}H_{12}N_2O$)**	**MW[a]** (g mol^{-1})	**Stokes radius** (nm)	**TPSA[b]** (nm^2)	**Water Solubility** (mg L^{-1})	**Molecular Volume** (nm^3)	**pK_a**	**D[c]**
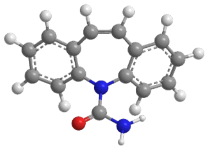	236.27	0.37	0.463	17.7	0.31	2.3	3.6

[a]–Molecular weight; [b]–topological polar surface area; [c]–dipole moment.

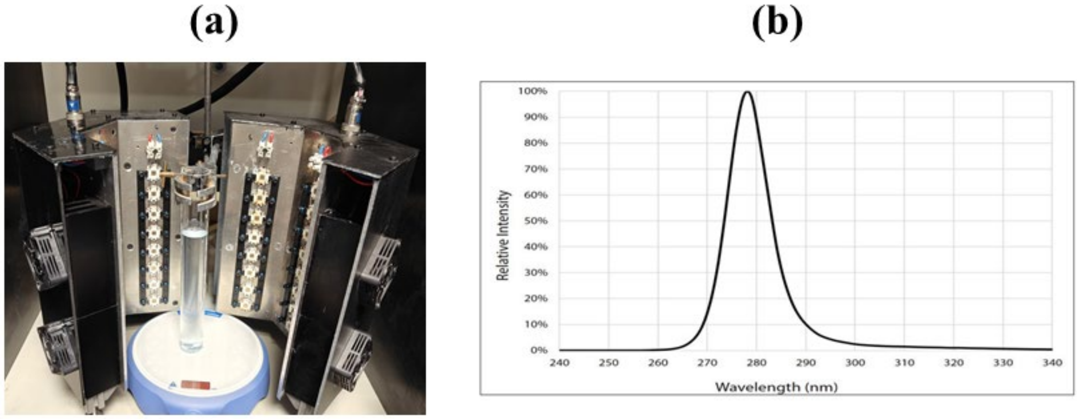

Figure S1. (a) LED system used in the photodegradation experiments; and (b) UV-vis emission spectrum of the LEDs (Luminus, mod XFM-5050-UV-D130-FE270-00) provided by the manufacturer.

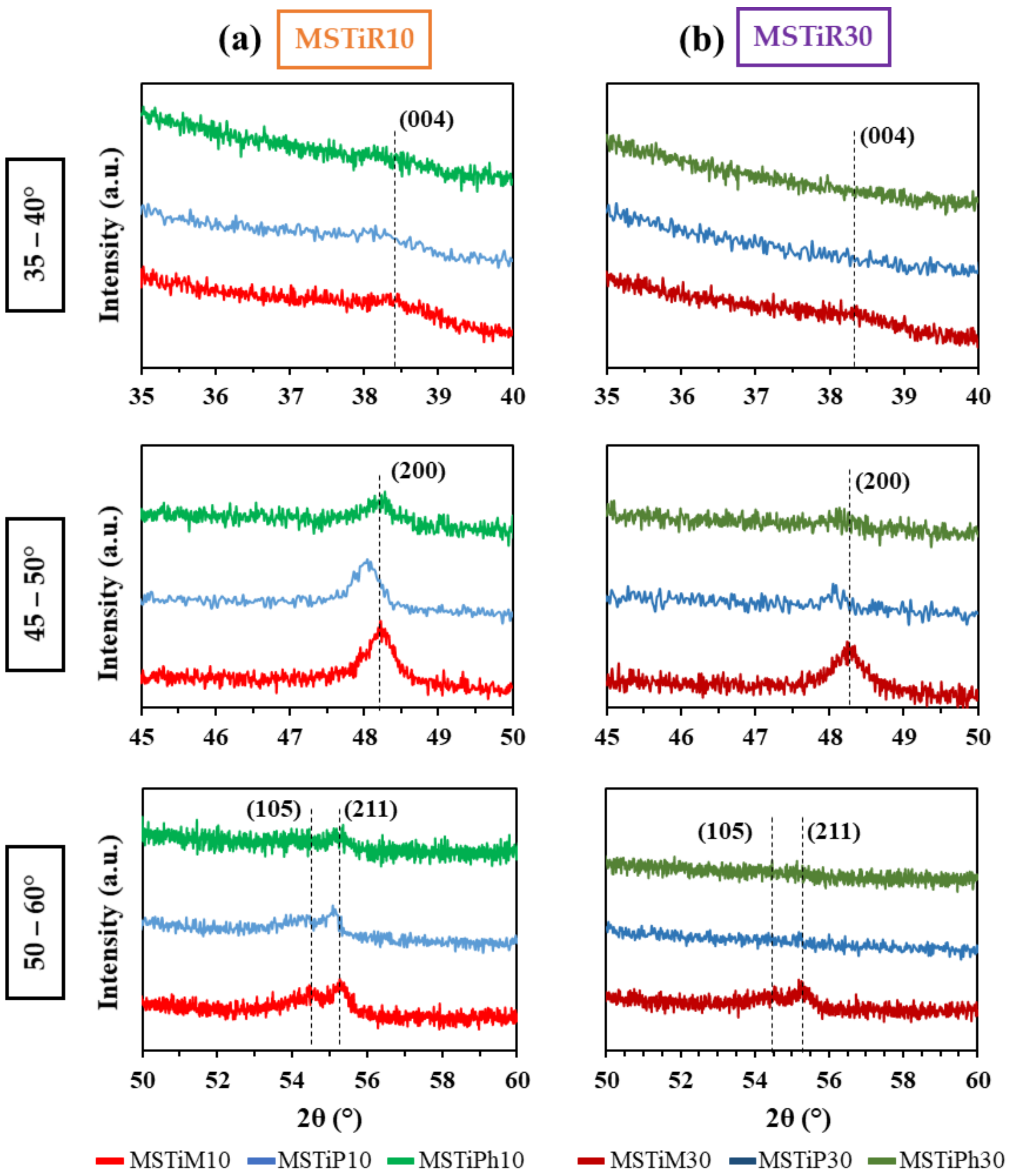

Figure S2. Zoomed XRD diffraction patterns of the (a) MSTiR10 and (b) MSTiR30 materials in the 35 – 40°, 45 – 50° and 50 – 60° ranges.

Table S2. Identified anatase diffraction maxima, recorded intensity (*I*), full width at half maximum (*FWHM*) and integrated area (*A*).

MSTiR%	(h, k, l)	2θ (°)	I (a.u.)	FWHM (°)	A (a.u.)
MSTiM10	(101)	25.48	7720	0.97	3195.91
	(200)	48.23	1924	0.33	366.35
	(105)	54.46	1468	[a]	[a]
	(211)	55.27	1519	0.41	188.60
MSTiP10	(101)	25.37	9909	0.90	4658.93
	(200)	48.05	2179	0.40	275.90
	(105)	54.39	1635	0.65	160.43
	(211)	55.08	1738	0.33	188.73
MSTiPh10	(101)	25.47	5998	0.91	2553.42
	(200)	48.28	1585	[a]	129.25
MSTiM30	(101)	25.53	6138	0.93	2516.91
	(200)	48.24	1693	0.38	195.72
	(105)	54.52	1403	[a]	152.12
	(211)	55.18	1461	[a]	140.75
MSTiP30	(101)	25.39	7093	0.90	3430.70
	(200)	48.05	1822	[a]	66.37
MSTiPh30	(101)	25.34	5943	0.76	2070.06
	(200)	48.13	1597	0.03	71.96

[a]-Unable to calculate

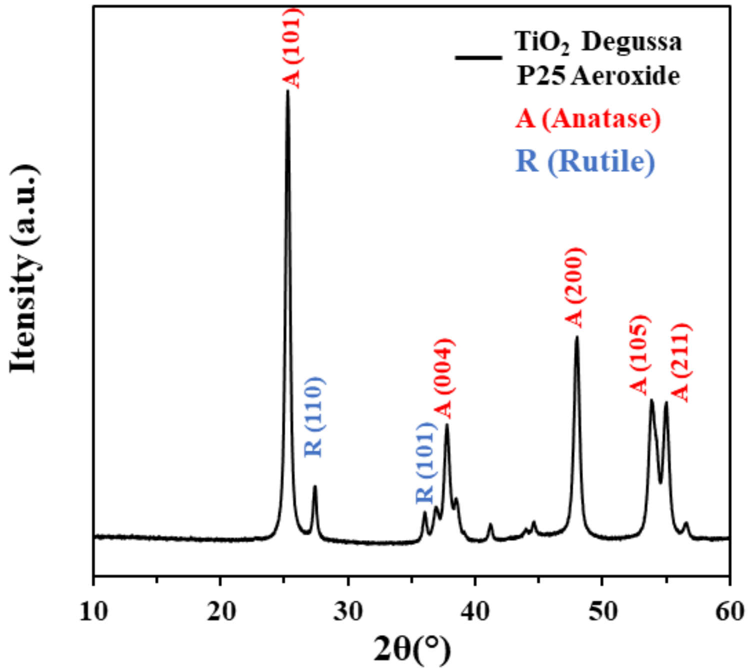

Figure S3. Diffraction patterns of the TiO_2 Degussa P25 Aeroxide to ilustrate the more intense diffraction maxima belonging to anatase and rutile in the 10 – 60° range.

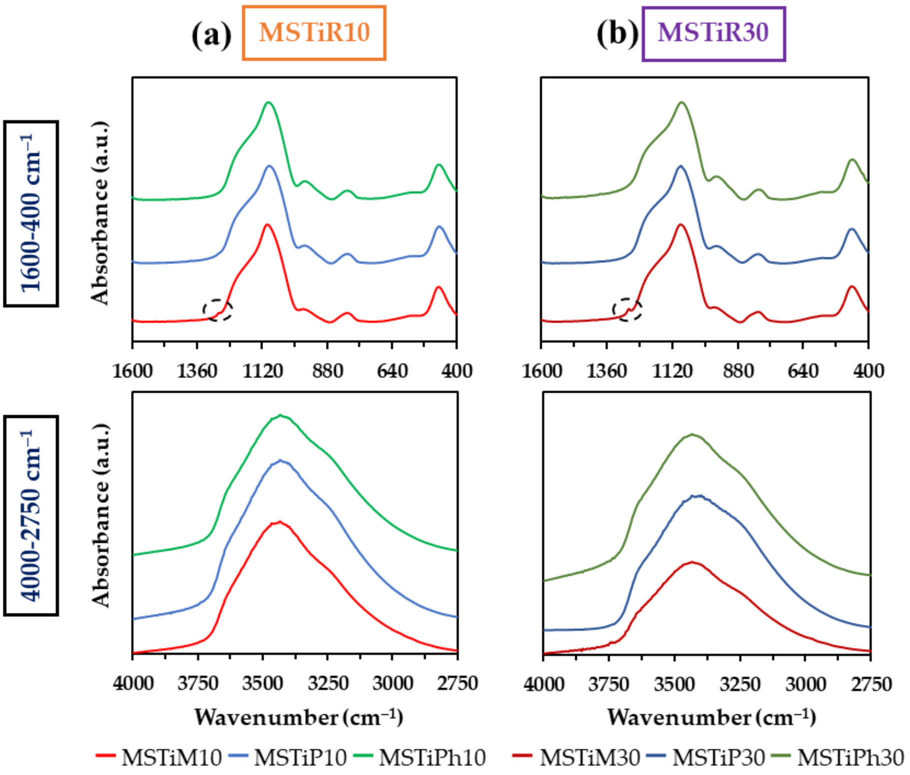

Figure S4. FTIR spectra of (a) MSTiR10 and (b) MSTiR30 in the 4000 – 2750 and 1600 – 400 cm^{-1} spectral range.

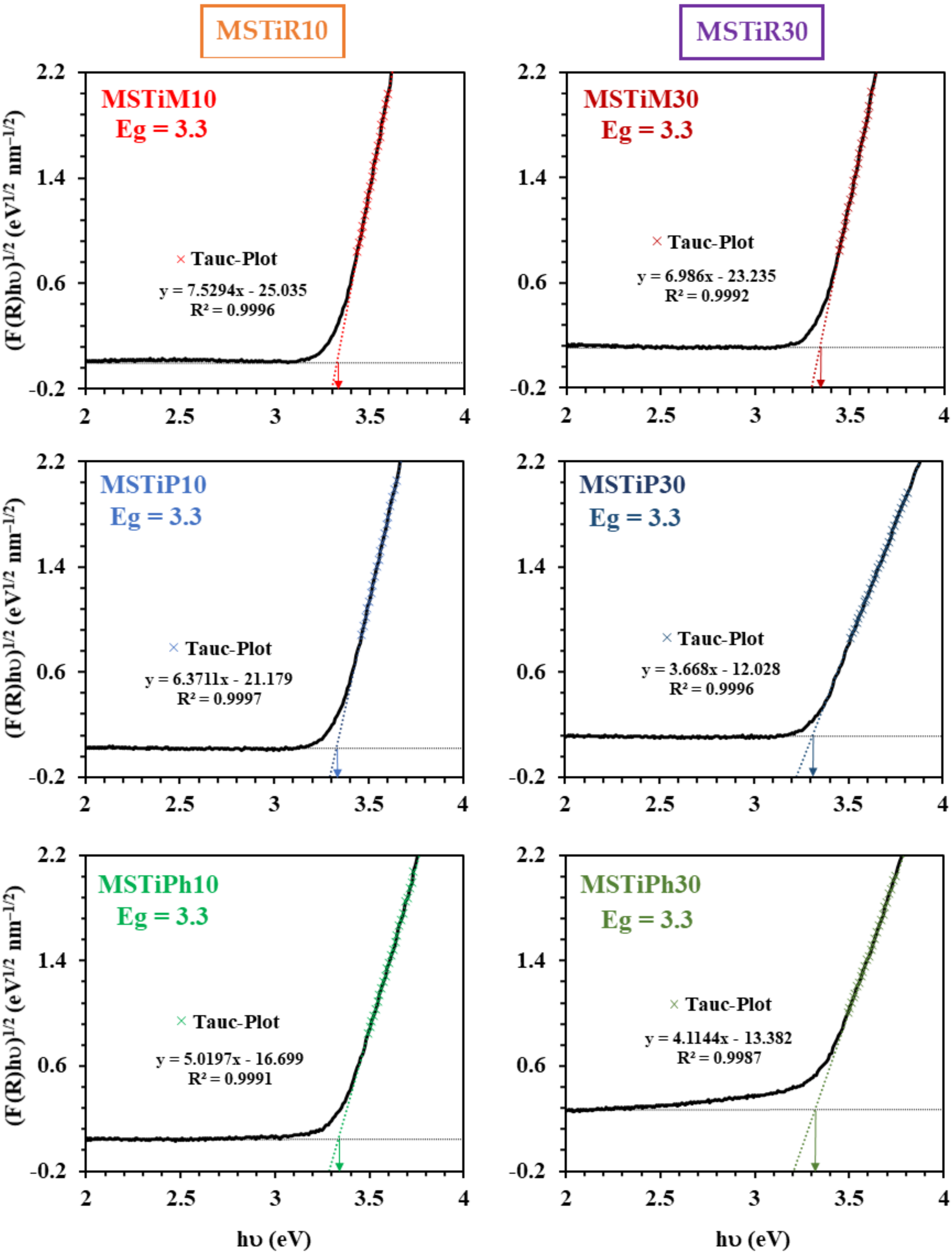

Figure S5. Tauc-plot analyses of the UV-Vis reflectance diffuse spectra of the (a) MSTiR10 and (b) MSTiR30 materials.

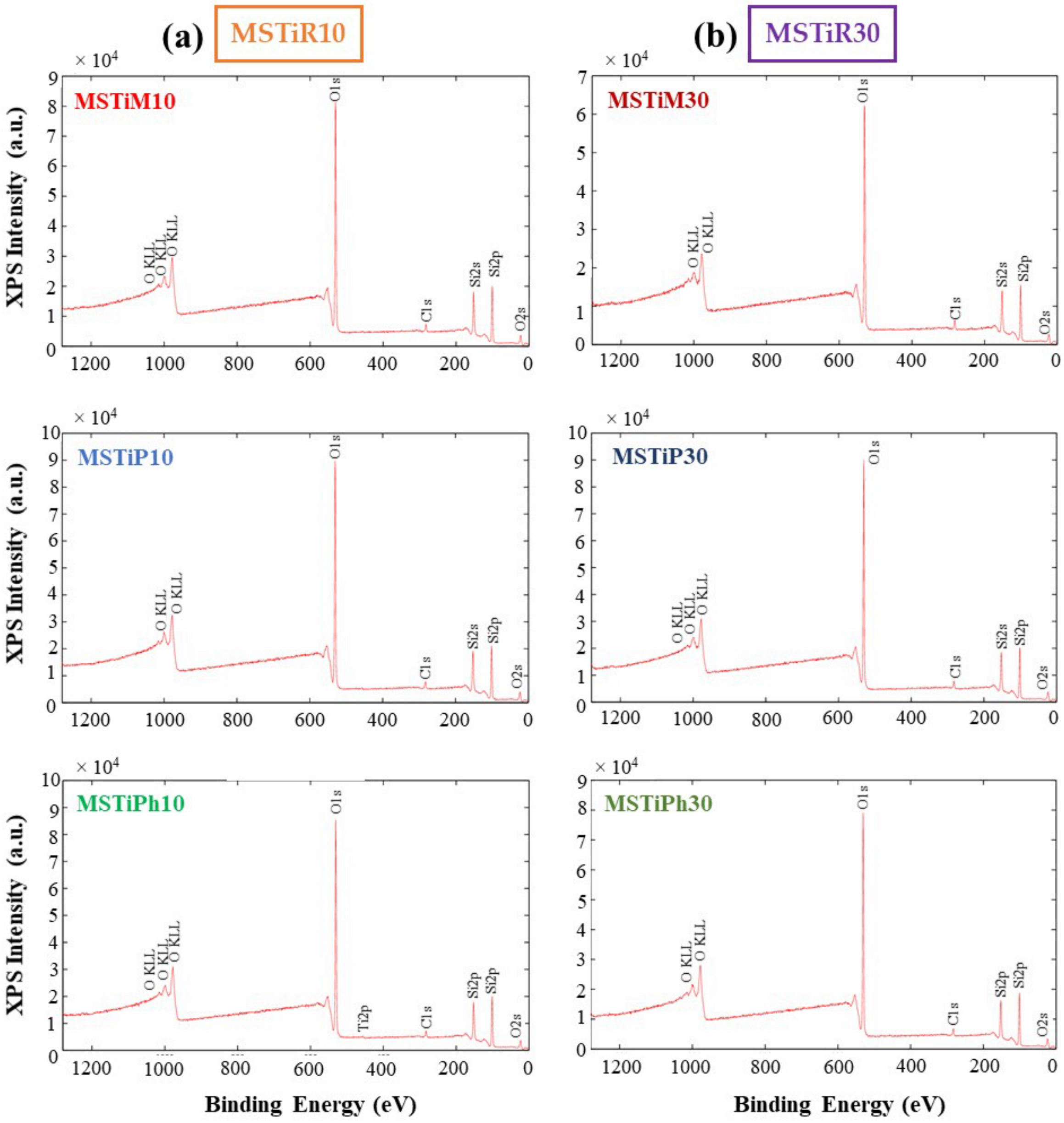

Figure S6. XPS spectra of the (a) MSTiR10 and (b) MSTiR30 materials and peak assignation.

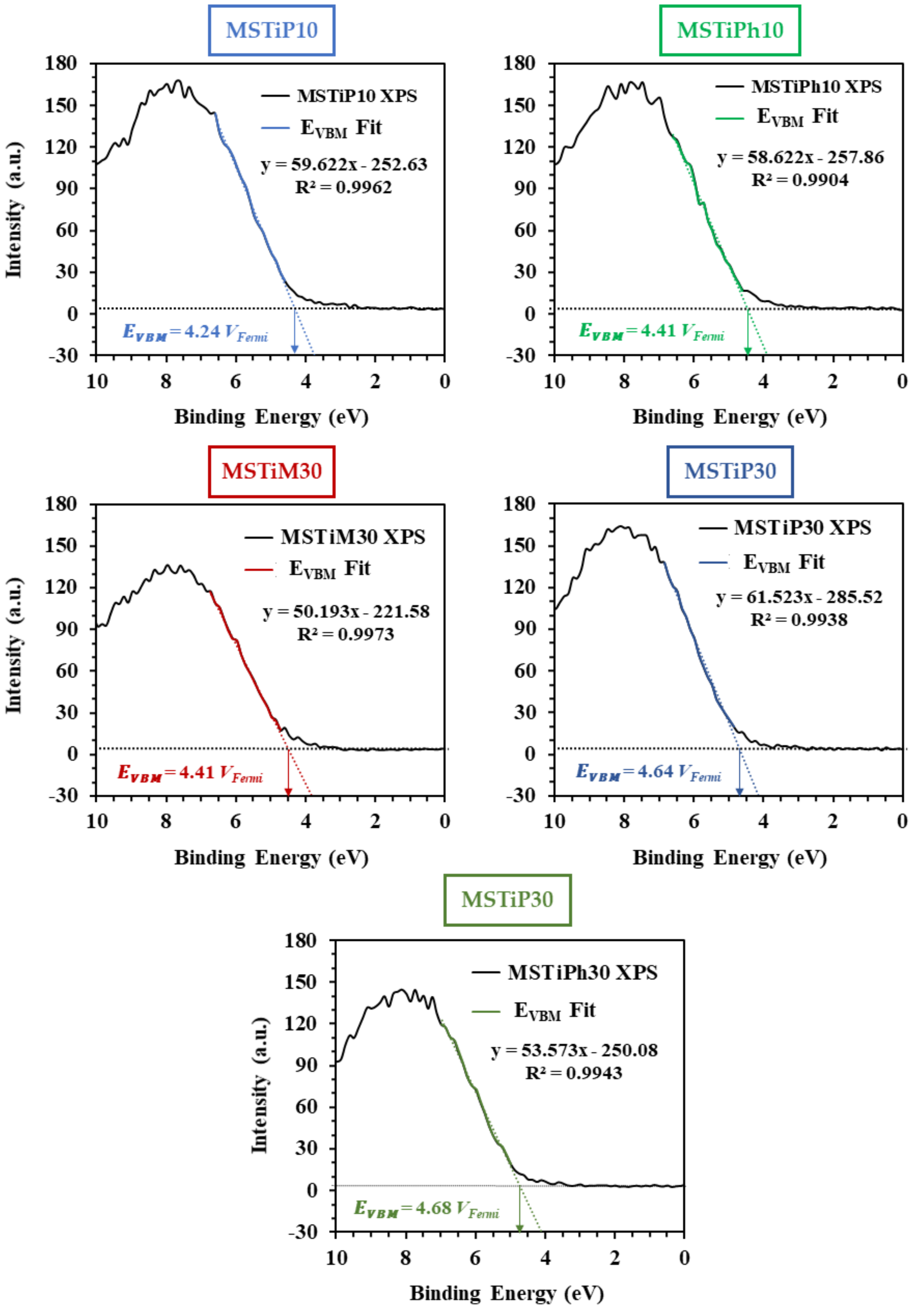

Figure S7. Linear fits of the MSTiP10, MSTiPh10, MSTiR30 spectra to determine their valence band maximum edge potential (E_{VBM}).

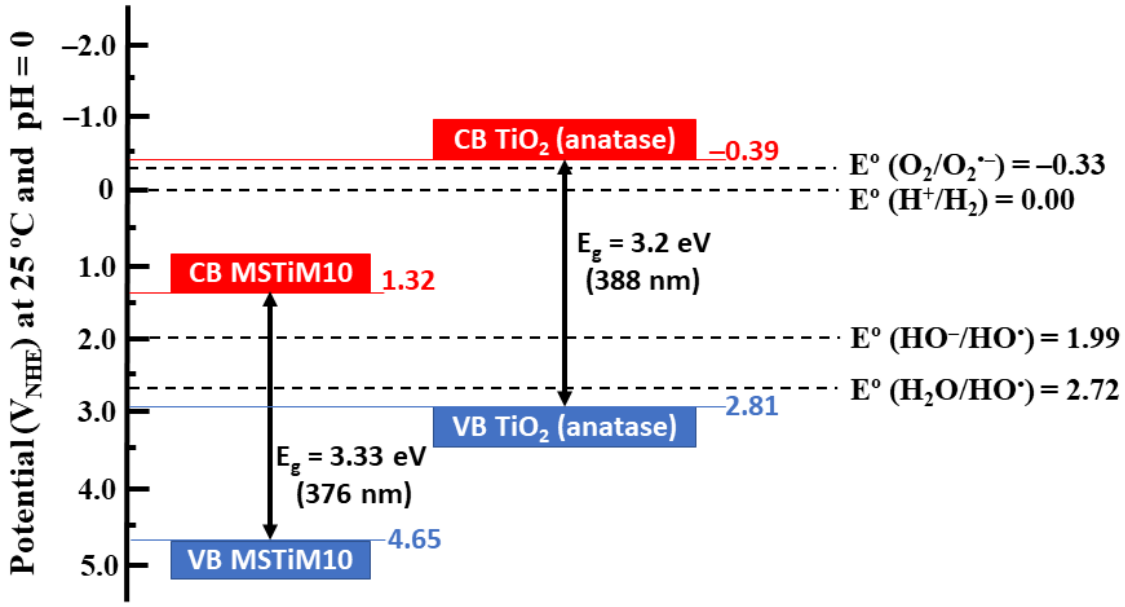

Figure S8. Bandgaps and valence and conduction bands (blue and red, respectively) of the MSTiM10 material and of pure anatase [56], and the redox potentials for the generation of hydroxyl ($HO^•$) and superoxide ($O_2^{•-}$) radicals [25].

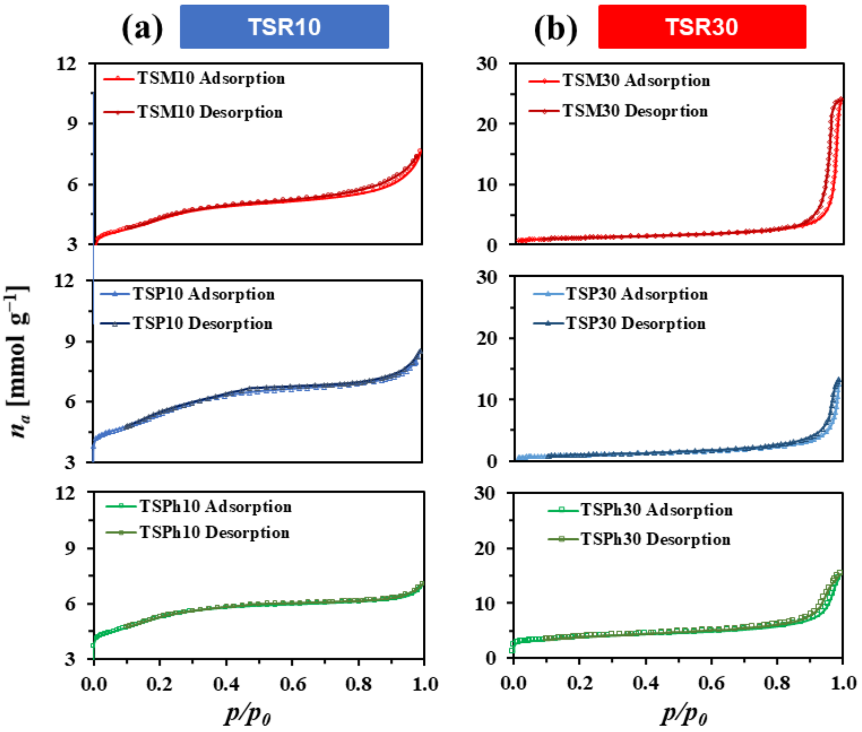

Figure S9. Isotherms of the materials prepared using TPAOH instead of TPBr: (a) titanosilicalites prepared with 10% of RTEOS; and (b) amorphous materials with no significant micro- or narrow mesoporosity prepared with 30% of RTEOS. Adapted from "Design of Novel Photoactive Modified Titanium Silicalites and their Application for Venlafaxine Degradation under Simulated Solar Irradiation", published in Solar RLL (Open access) in 2024 and authored by Cruz-Quesada G. et al. [12]. Copyright © 2024, Wiley-VCH GmbH, Weinheim.

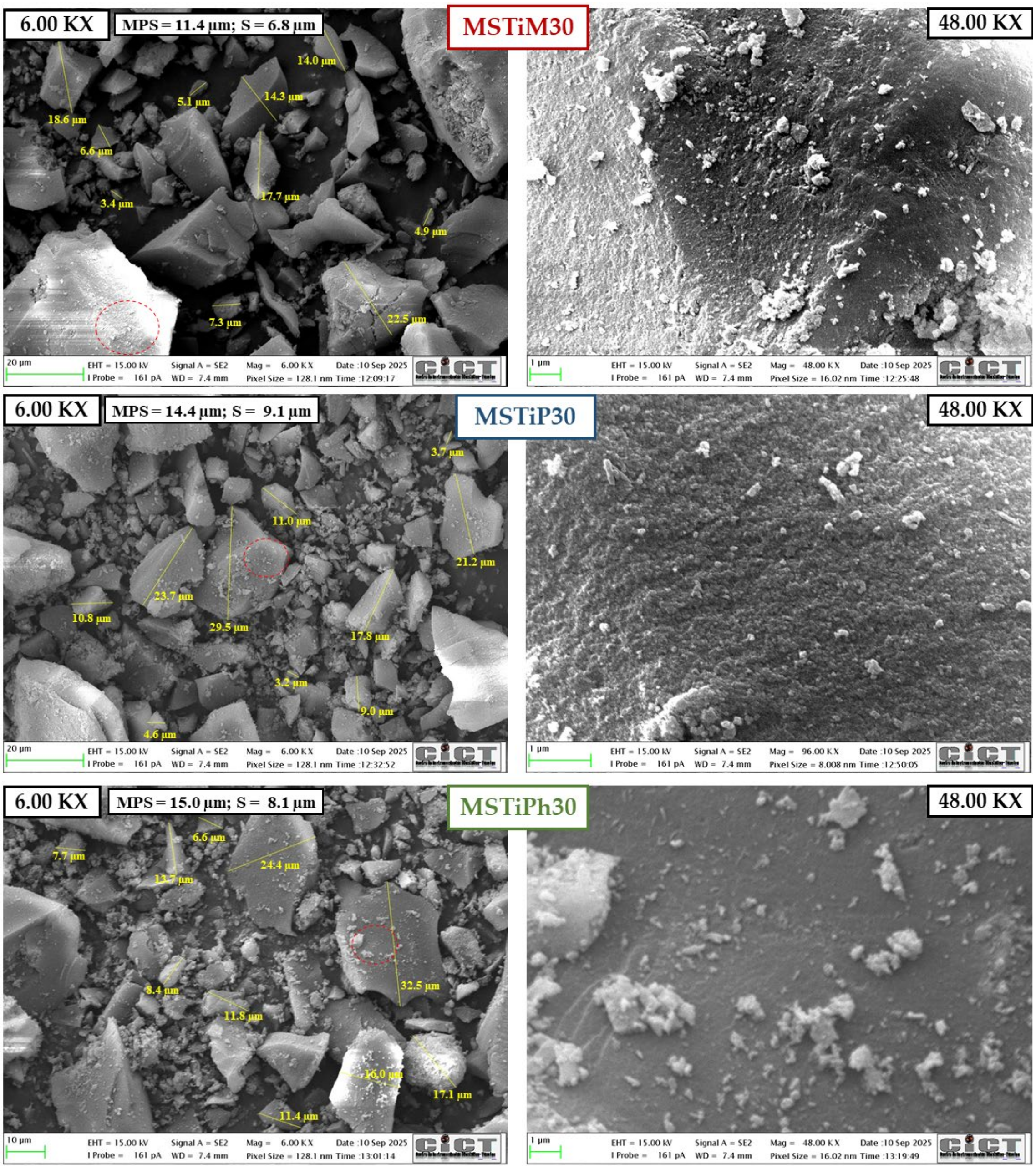

Figure S10. FE-SEM micrographs of the MSTiR30 materials obtained at magnifications of 6.00 KX and 48.00 KX (particles in red circles in 6.00 KX micrographs). MPS = mean particle size; S = standard deviation.

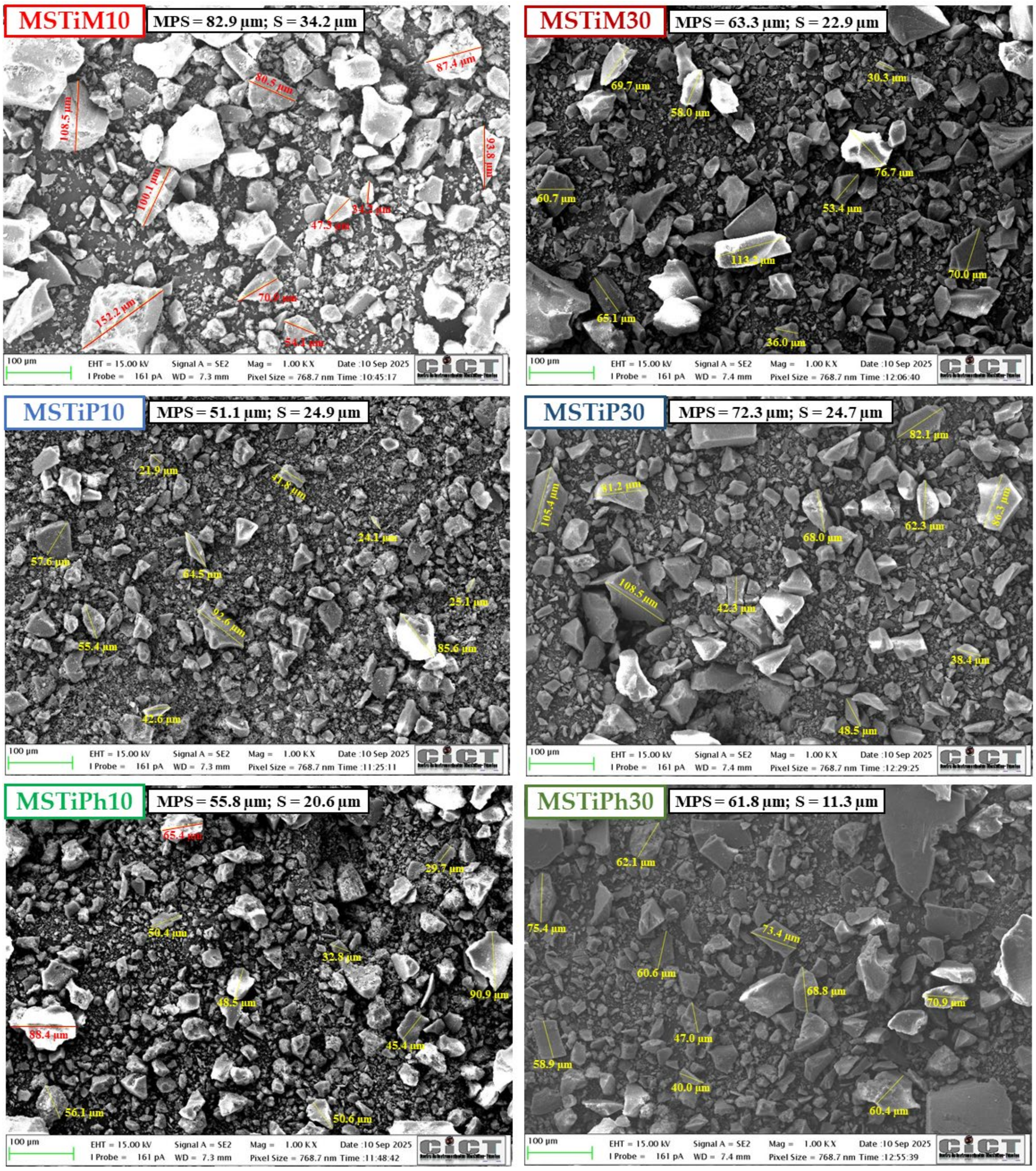

Figure S11. FE-SEM micrographs of the MSTiR% materials obtained at magnifications of 1.00 KX. MPS = mean particle size; S = standard deviation.

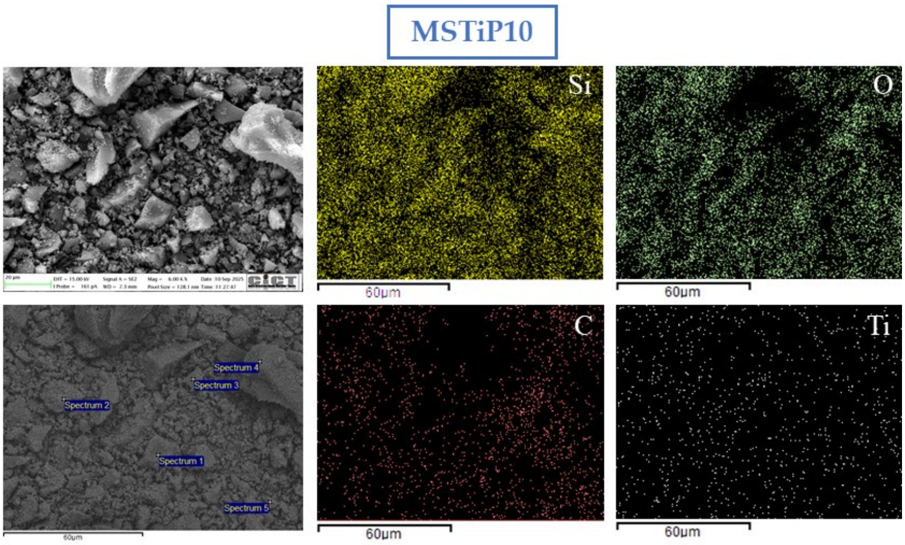

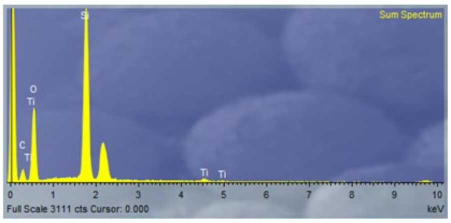

Spectrum	C	O	Si	Ti
	Weight (%)			
1	18.86	37.38	43.76	
2	15.59	49.33	35.08	
3	19.30	28.17	52.53	
4	15.17	53.78	30.31	0.74
5	21.30	50.80	27.90	
Sum	21.63	46.04	30.98	1.36

Figure S12. EDX mapping analysis of the MSTiP10 6.0 KX micrograph and relative abundances of C, O, Si and Ti calculated from the acquired spectra of different particles and from their sum spectra.

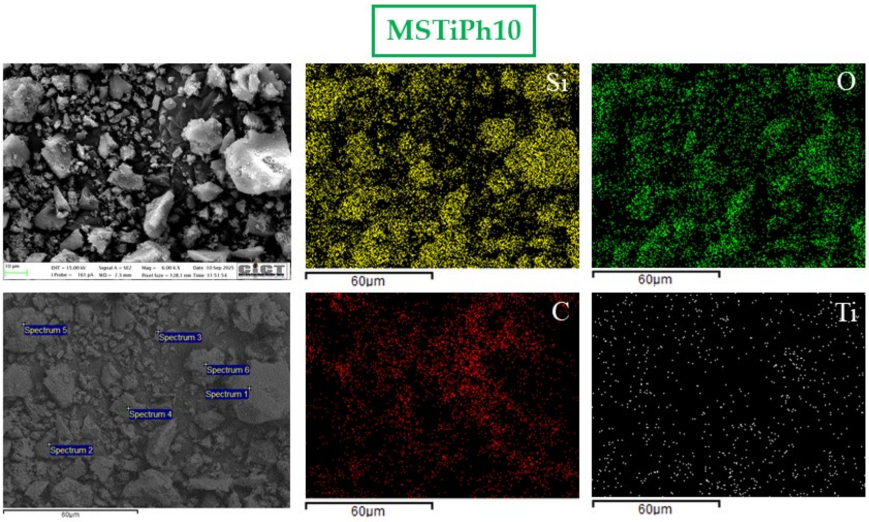

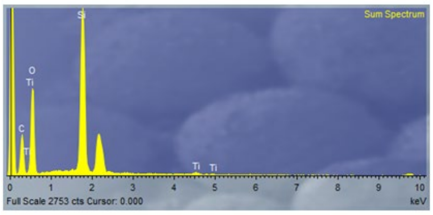

Spectrum	C	O	Si	Ti
	Weight (%)			
1	13.45	46.87	37.81	1.87
2	13.10	51.05	35.85	
3	33.43	45.72	20.85	
4	14.22	45.67	40.11	
5	16.87	48.20	34.19	0.74
6	10.61	42.15	44.65	2.59
Sum	34.41	43.30	21.18	1.10

Figure S13. EDX mapping analysis of the MSTiPh10 6.0 KX micrograph and relative abundances of C, O, Si and Ti calculated from the acquired spectra of different particles and from their sum spectra.

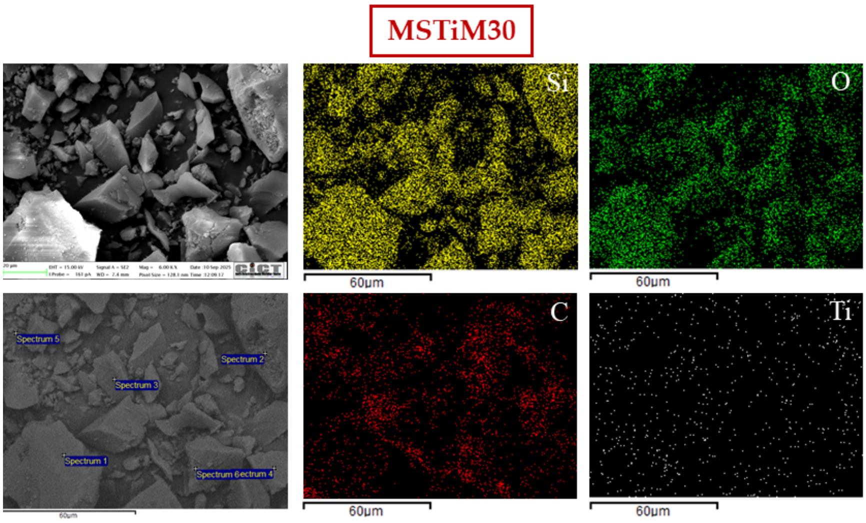

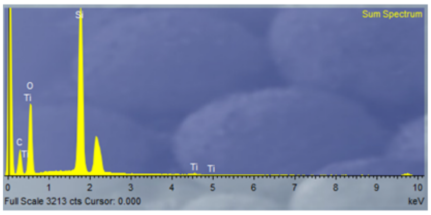

Spectrum	C	O	Si	Ti
	Weight (%)			
1	11.61	47.85	40.54	
2	8.85	42.22	45.84	3.09
3	15.44	43.13	41.43	
4	19.73	42.58	37.70	
5	50.21	36.24	12.44	1.10
6	21.27	48.22	29.93	0.58
Sum	31.39	42.05	26.03	0.52

Figure S14. EDX mapping analysis of the MSTiM30 6.0 KX micrograph and relative abundances of C, O, Si and Ti calculated from the acquired spectra of different particles and from their sum spectra.

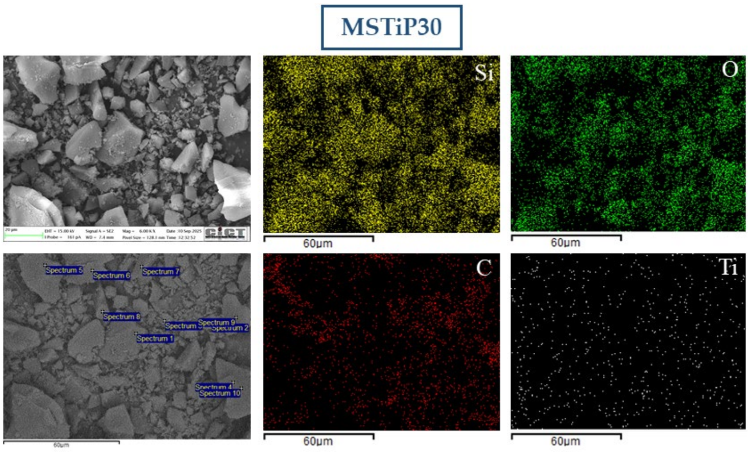

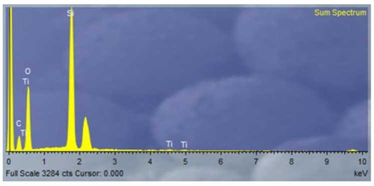

Spectrum	C	O	Si	Ti
	Weight (%)			
1	15.42	39.25	45.32	
2	23.16	45.92	30.92	
3	26.11	45.68	28.21	
4	13.58	53.30	32.14	0.97
5	13.86	48.62	37.52	
6	9.62	37.53	52.85	
7	16.61	51.28	32.11	
8	11.09	50.73	38.18	
9	17.48	52.11	29.72	0.69
10	12.35	52.20	35.45	
Sum	26.02	45.07	28.21	0.70

Figure S15. EDX mapping analysis of the MSTiP30 6.0 KX micrograph and relative abundances of C, O, Si and Ti calculated from the acquired spectra of different particles and from their sum spectra.

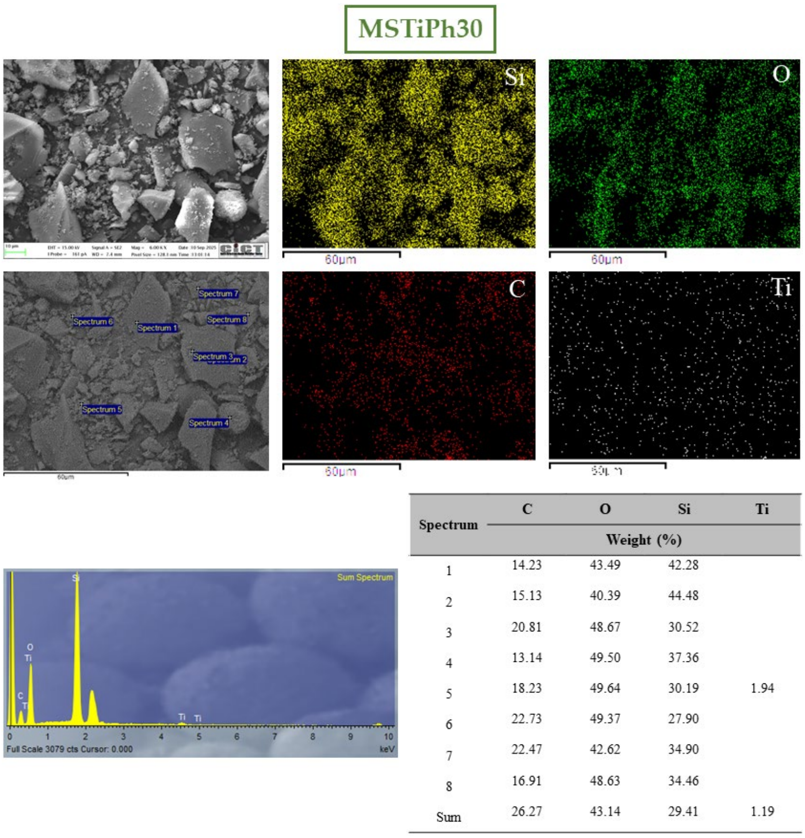

Spectrum	C	O	Si	Ti
	Weight (%)			
1	14.23	43.49	42.28	
2	15.13	40.39	44.48	
3	20.81	48.67	30.52	
4	13.14	49.50	37.36	
5	18.23	49.64	30.19	1.94
6	22.73	49.37	27.90	
7	22.47	42.62	34.90	
8	16.91	48.63	34.46	
Sum	26.27	43.14	29.41	1.19

Figure S16. EDX mapping analysis of the MSTiPh30 6.0 KX micrograph and relative abundances of C, O, Si and Ti calculated from the acquired spectra of different particles and from their sum spectra.

Table S3. Removal of CBZ, First-order kinetic constant (K_{app}), and half-life ($t_{1/2}$) for each catalyst dose of the MSTiR% materials.

MSTiR%	MSTiR% Dose (g L^{-1})	CBZ Removal (%) Adsorbed[a]	Degraded[b]	Total	First-Order Kinetic Adjustment k_{app}[c] (min^{-1})	$t_{1/2}$[d] (min)	R^2
None	0.00	-	17.36	17.36	0.0018	391.28	0.9958
MSTiM10	0.25	1.78	96.92	98.70	0.0430	16.12	0.9812
	0.50	5.00	92.58	97.58	0.0448	15.46	0.9984
	0.75	6.47	90.19	96.66	0.0404	17.14	0.9985
MSTiP10	0.25	0.00	97.92	97.92	0.0331	20.92	0.9912
	0.50	4.91	92.50	97.41	0.0512	13.54	0.9939
	0.75	6.49	91.70	98.19	0.0707	9.80	0.9844
	1.00	7.73	90.85	98.58	0.0877	7.90	0.9972
MSTiPh10	0.25	1.78	92.63	94.41	0.0265	26.11	0.9948
	0.50	5.70	92.48	98.18	0.0511	13.57	0.9980
	0.75	8.32	86.40	94.72	0.0356	19.48	0.9988
MSTiM30	0.50	10.97	81.41	92.38	0.0302	22.95	0.9993
MSTiP30	0.50	5.85	61.78	67.63	0.0099	70.32	0.9947
MSTiPh30	0.50	4.24	76.51	80.75	0.0166	35.53	0.9997

[a] Percentage of CBZ adsorbed after 60 min at dark; [b] percentage of CBZ degraded after 120 min of irradiation; [c] apparent First-order kinetic constant; [d] half-life calculated from k_{app}.

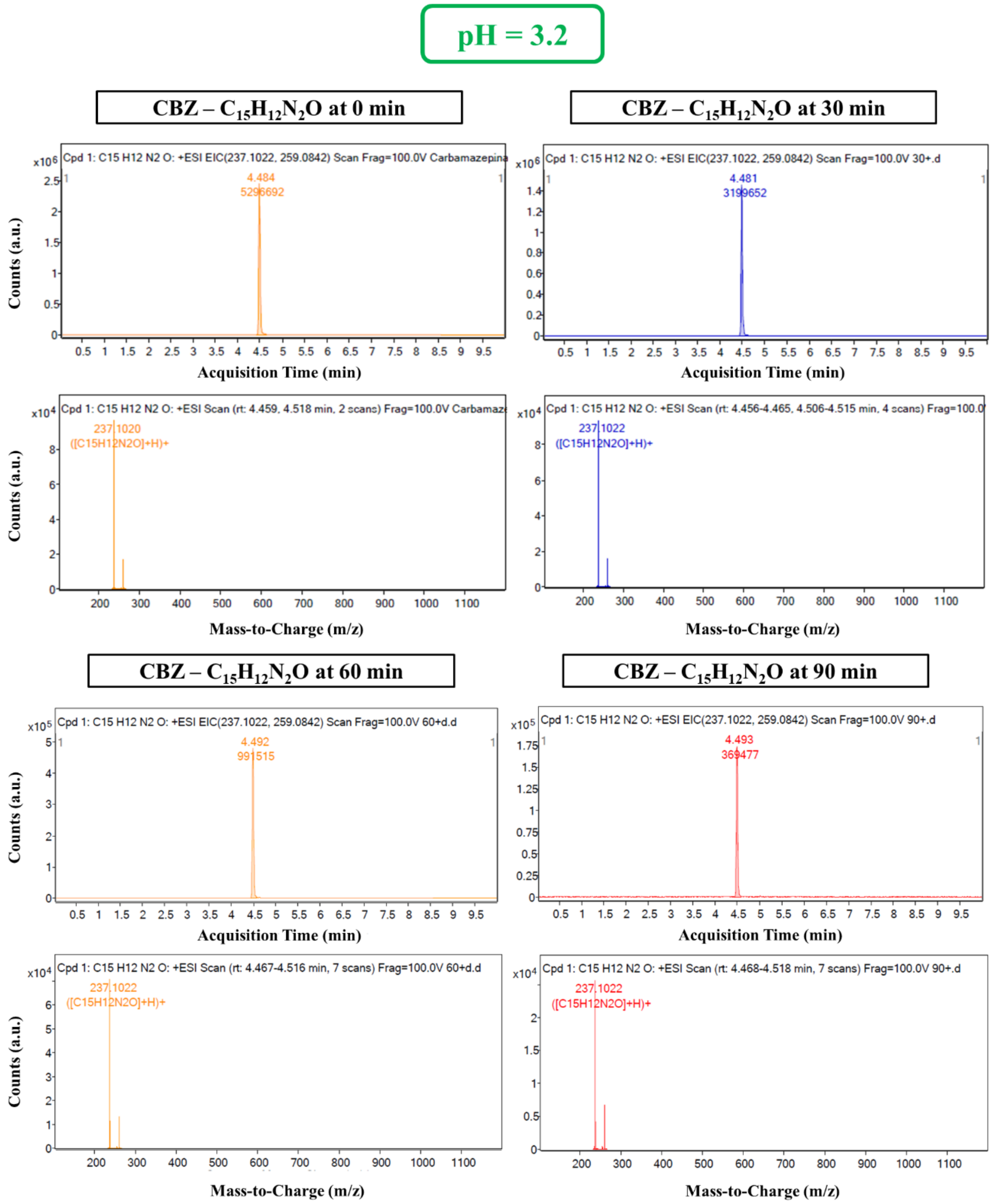

Figure S17. HPLC chromatograms and mass spectra of carbamazepine (CBZ) acquired from aliquots removed at t = 0, 30, 60, and 90 min from the photodegradation reaction of 1 mg L^{-1} of CBZ using 0.5 g L^{-1} of MSTiM10 at pH = 3.2.

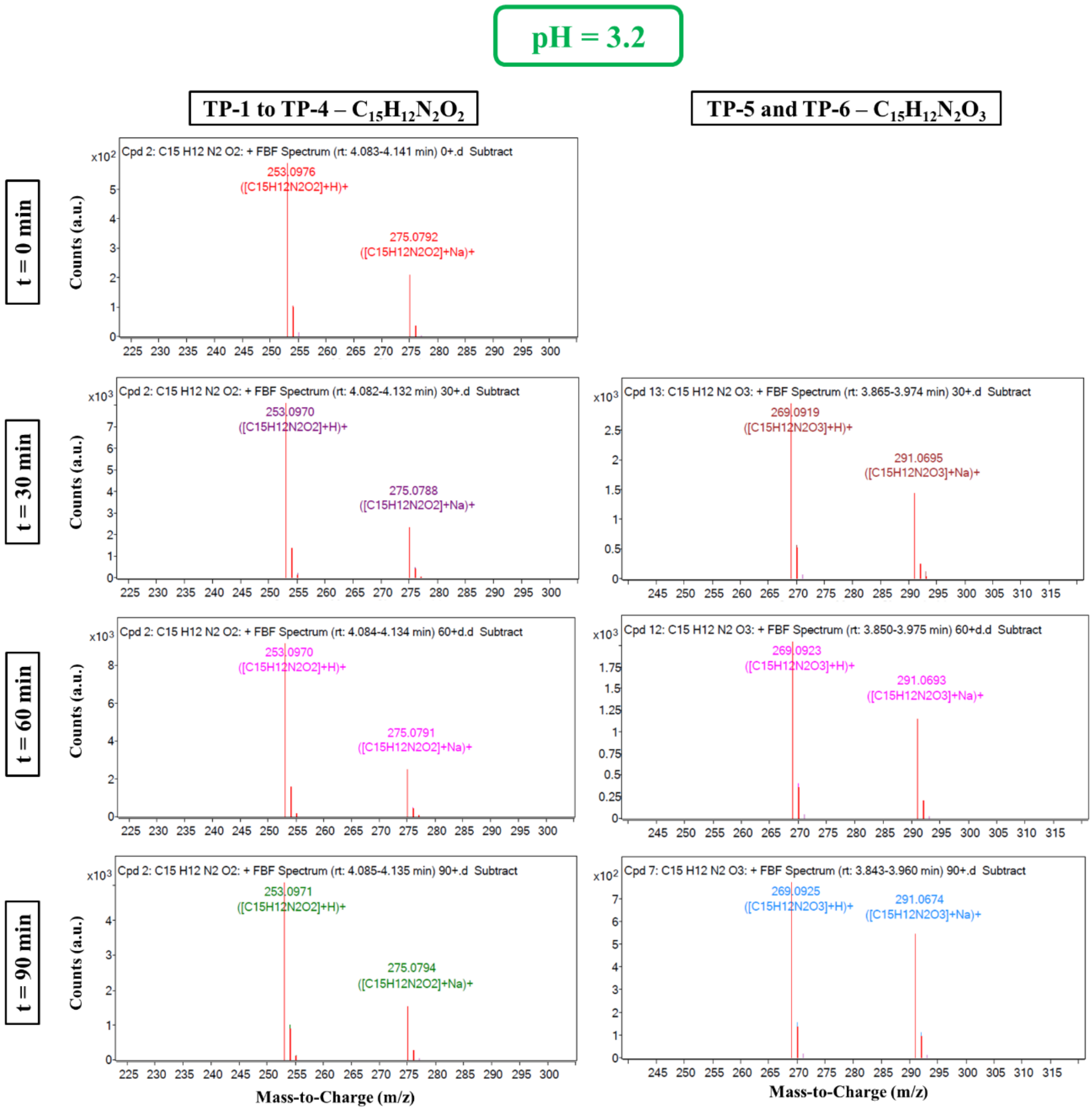

Figure S18. Mass spectra showing the detected transformation products TP-1 to TP-6 in the aliquots removed at t = 0, 30, 60, and 90 min from the photodegradation reaction of 1 mg L^{-1} of CBZ using 0.5 g L^{-1} of MSTiM10 at pH = 3.2.

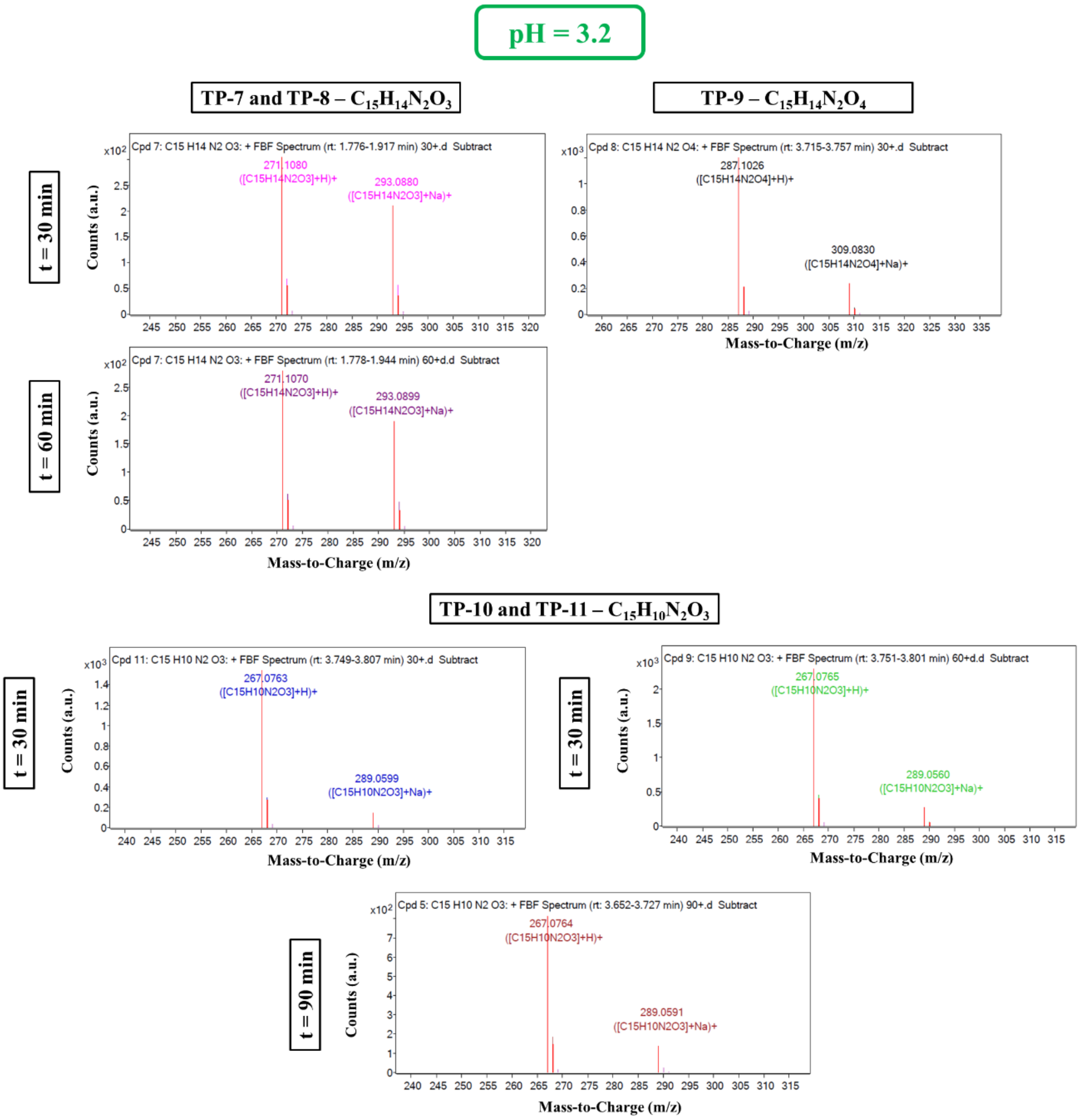

Figure S19. Mass spectra showing the detected transformation products TP-7 to TP-11 in the aliquots removed at t = 0, 30, 60, and 90 min from the photodegradation reaction of 1 mg L^{-1} of CBZ using 0.5 g L^{-1} of MSTiM10 at pH = 3.2.

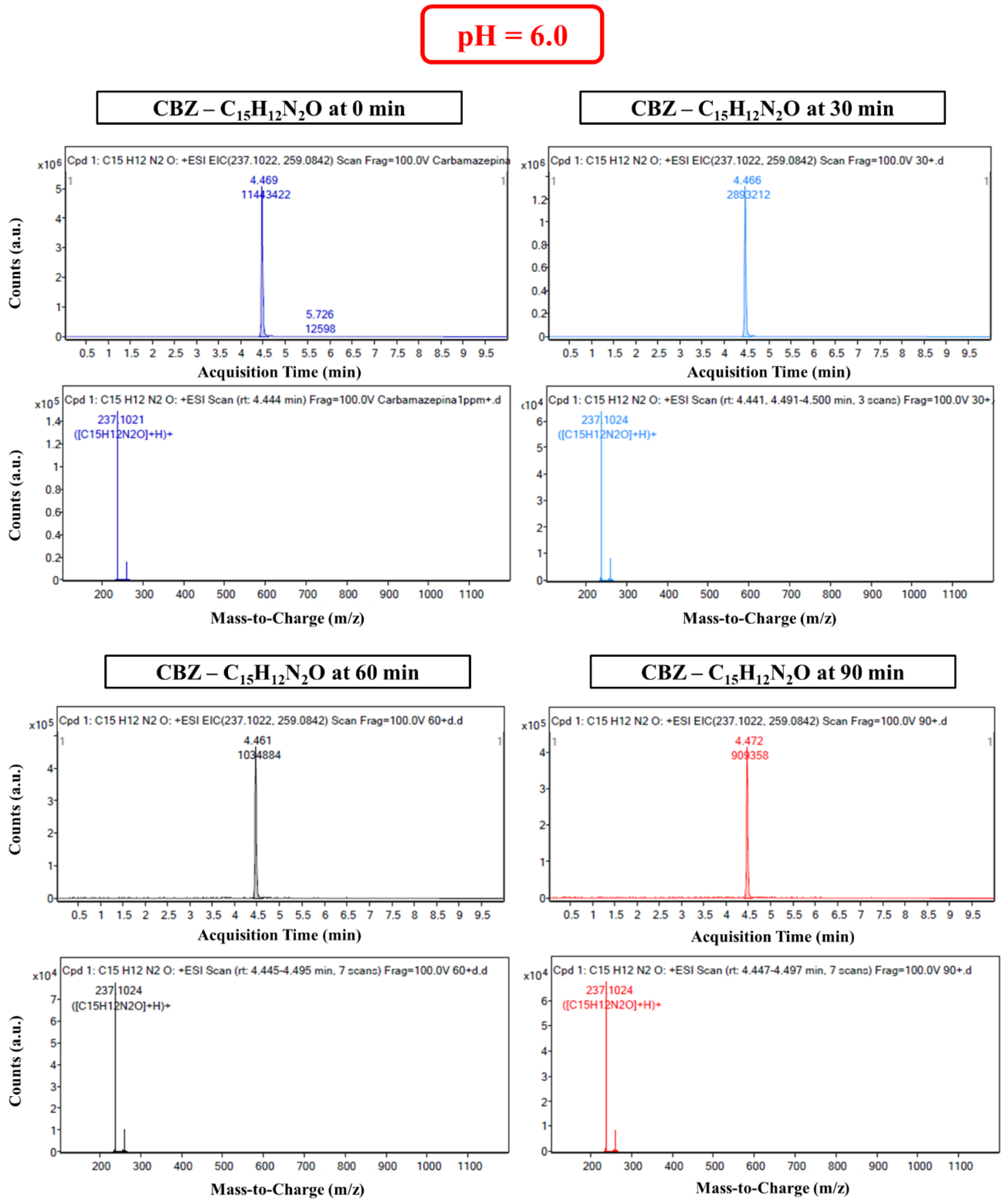

Figure S20. HPLC chromatograms and mass spectra of carbamazepine (CBZ) acquired from aliquots removed at t = 0, 30, 60, and 90 min from the photodegradation reaction of 1 mg L^{-1} of CBZ using 0.5 g L^{-1} of MSTiM10 at pH = 6.0.

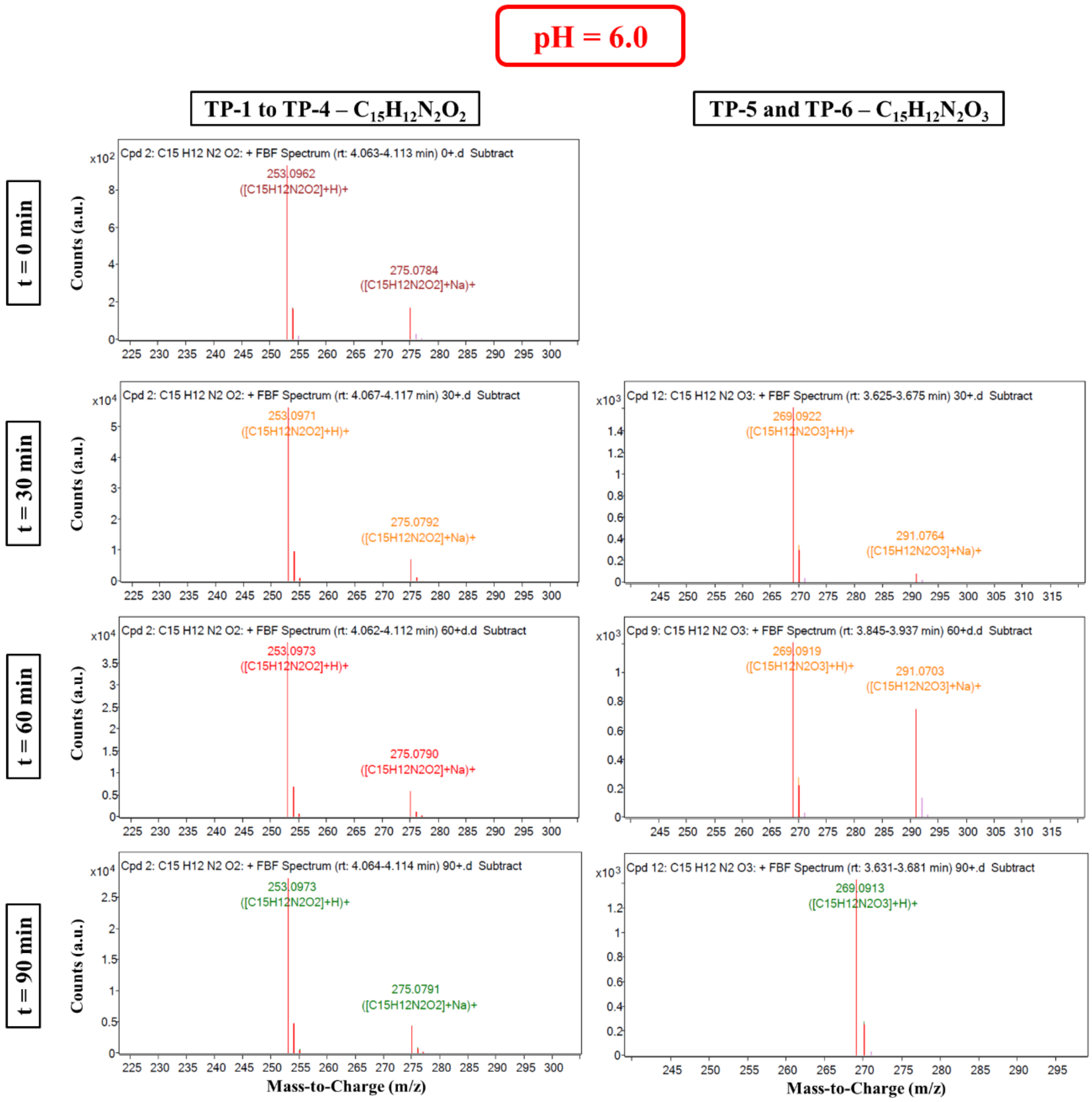

Figure S21. Mass spectra showing the detected transformation products TP-1 to TP-6 in the aliquots removed at t = 0, 30, 60, and 90 min from the photodegradation reaction of 1 mg L^{-1} of CBZ using 0.5 g L^{-1} of MSTiM10 at pH = 6.0.

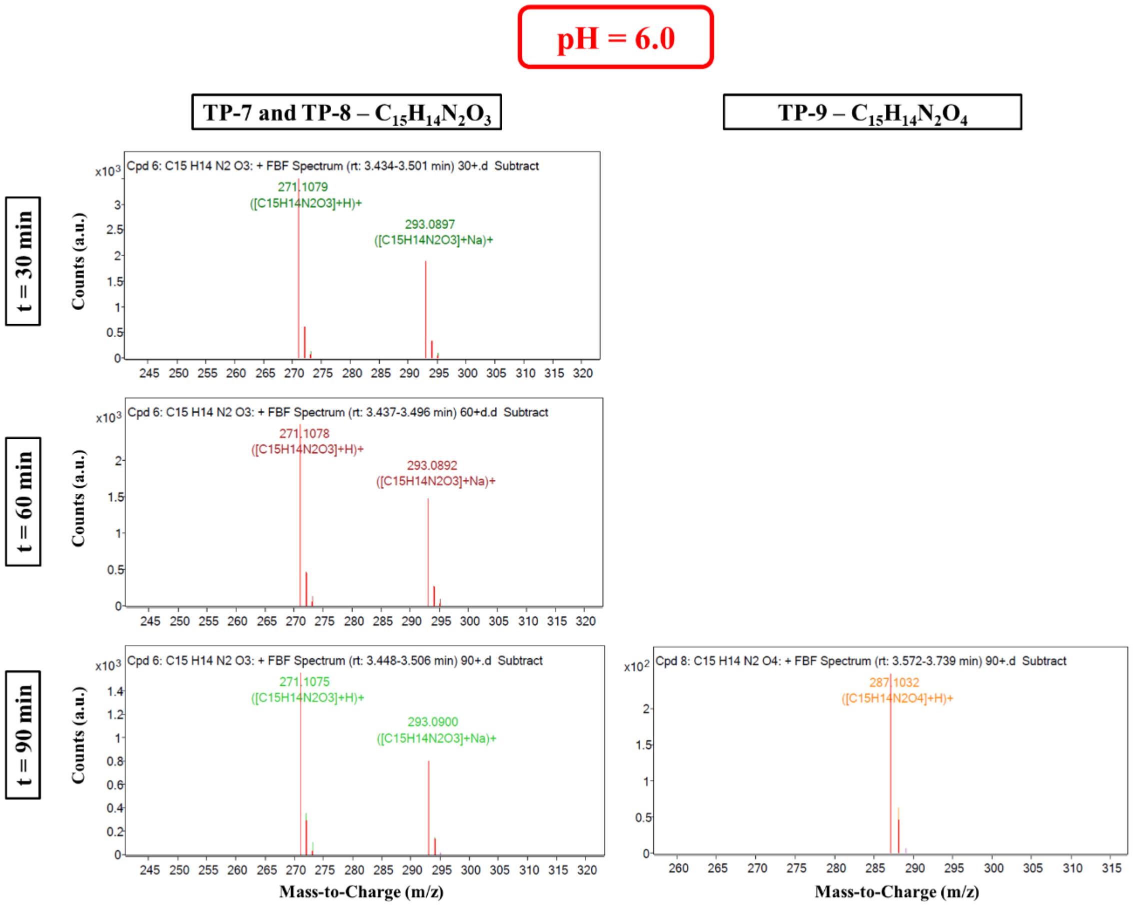

Figure S22. Mass spectra showing the detected transformation products TP-7 to TP-9 in the aliquots removed at t = 0, 30, 60, and 90 min from the photodegradation reaction of 1 mg L^{-1} of CBZ using 0.5 g L^{-1} of MSTiM10 at pH = 6.0.

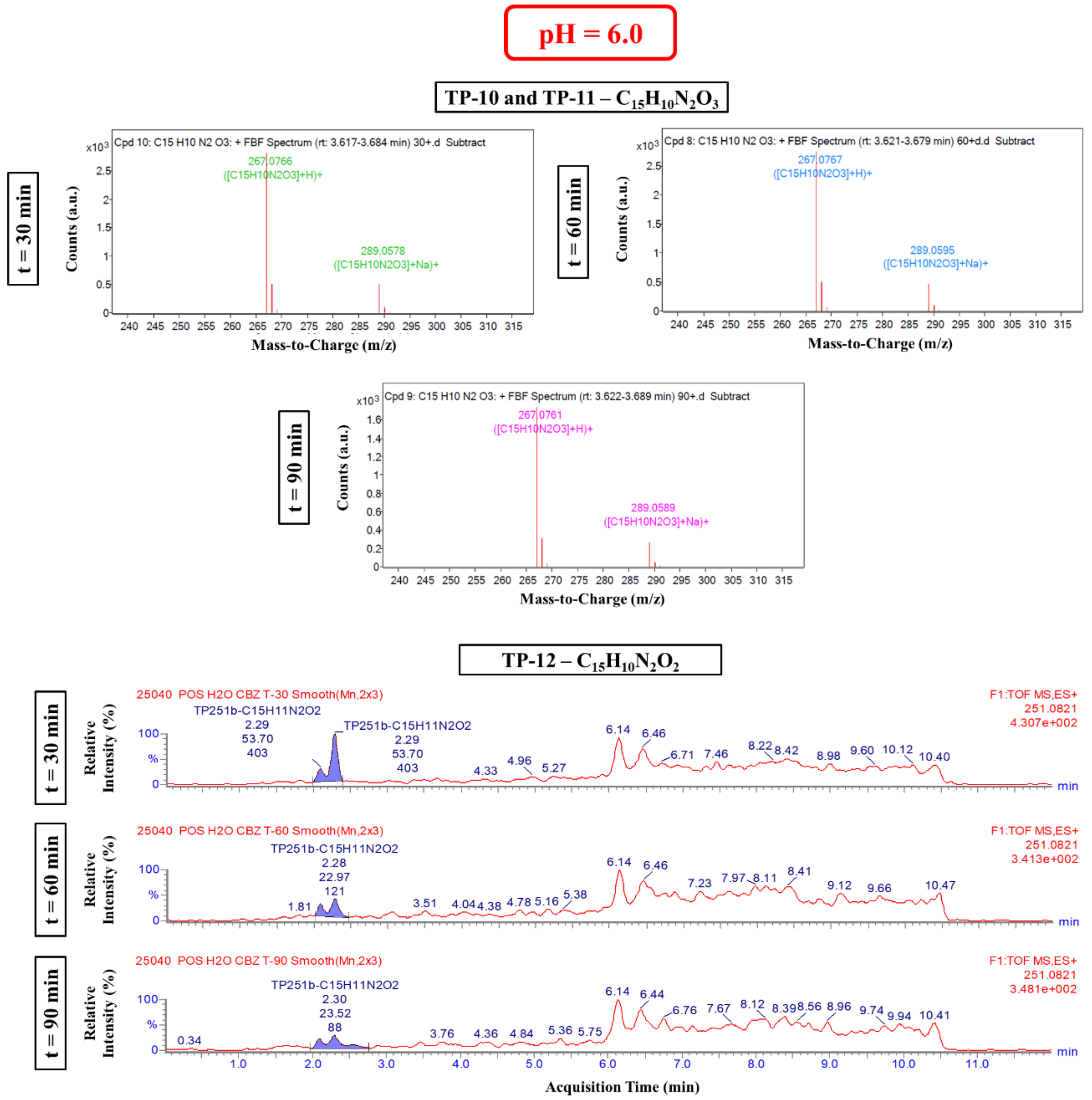

Figure S23. Mass spectra showing the detected transformation products TP-10 and TP-11, and HPLC chromatograms showing the detected TP-12 in the aliquots removed at t = 0, 30, 60, and 90 min from the photodegradation reaction of 1 mg L^{-1} of CBZ using 0.5 g L^{-1} of MSTiM10 at pH = 6.0.

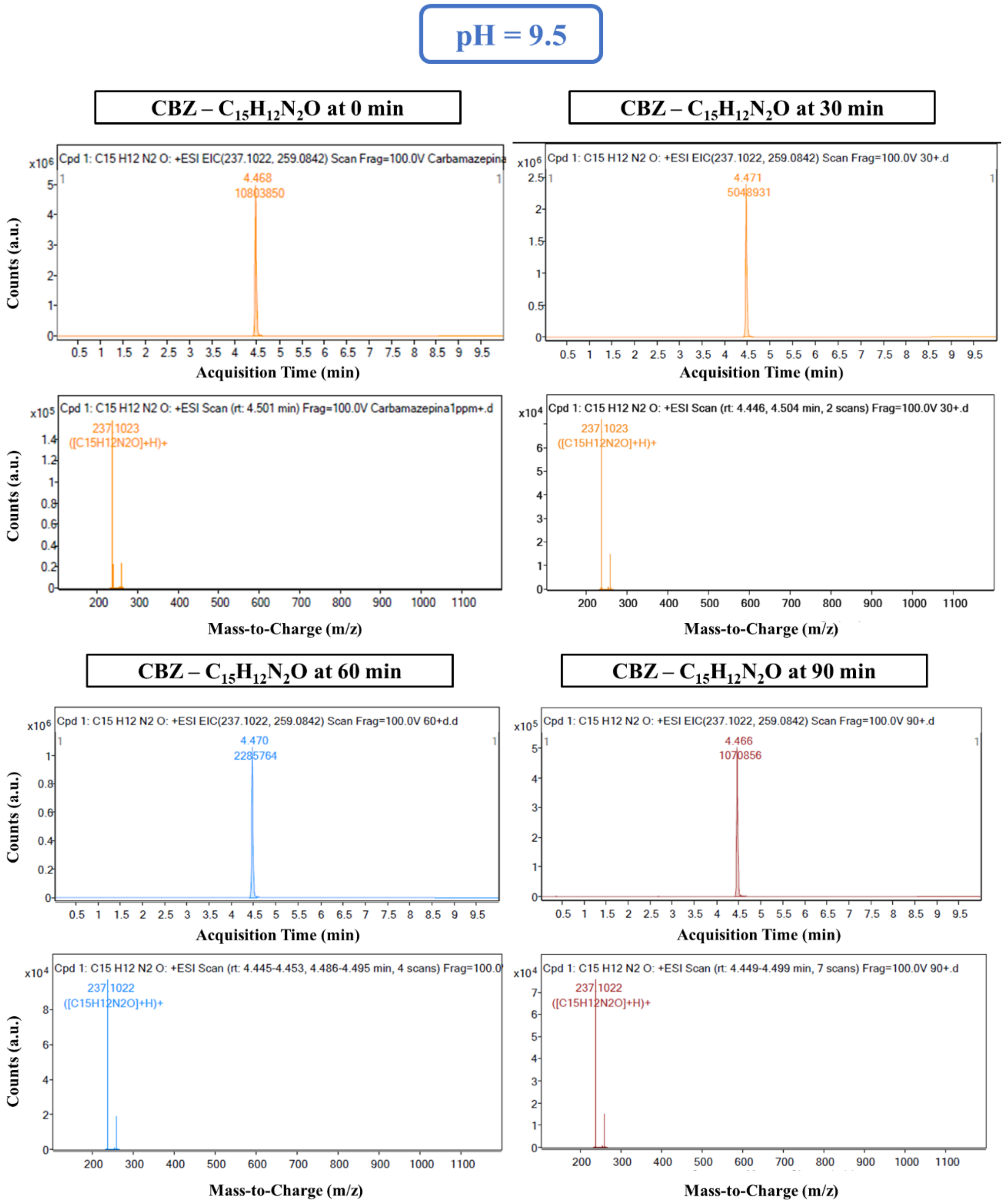

Figure S24. HPLC chromatograms and mass spectra of carbamazepine (CBZ) acquired from aliquots removed at t = 0, 30, 60, and 90 min from the photodegradation reaction of 1 mg L^{-1} of CBZ using 0.5 g L^{-1} of MSTiM10 at pH = 9.5.

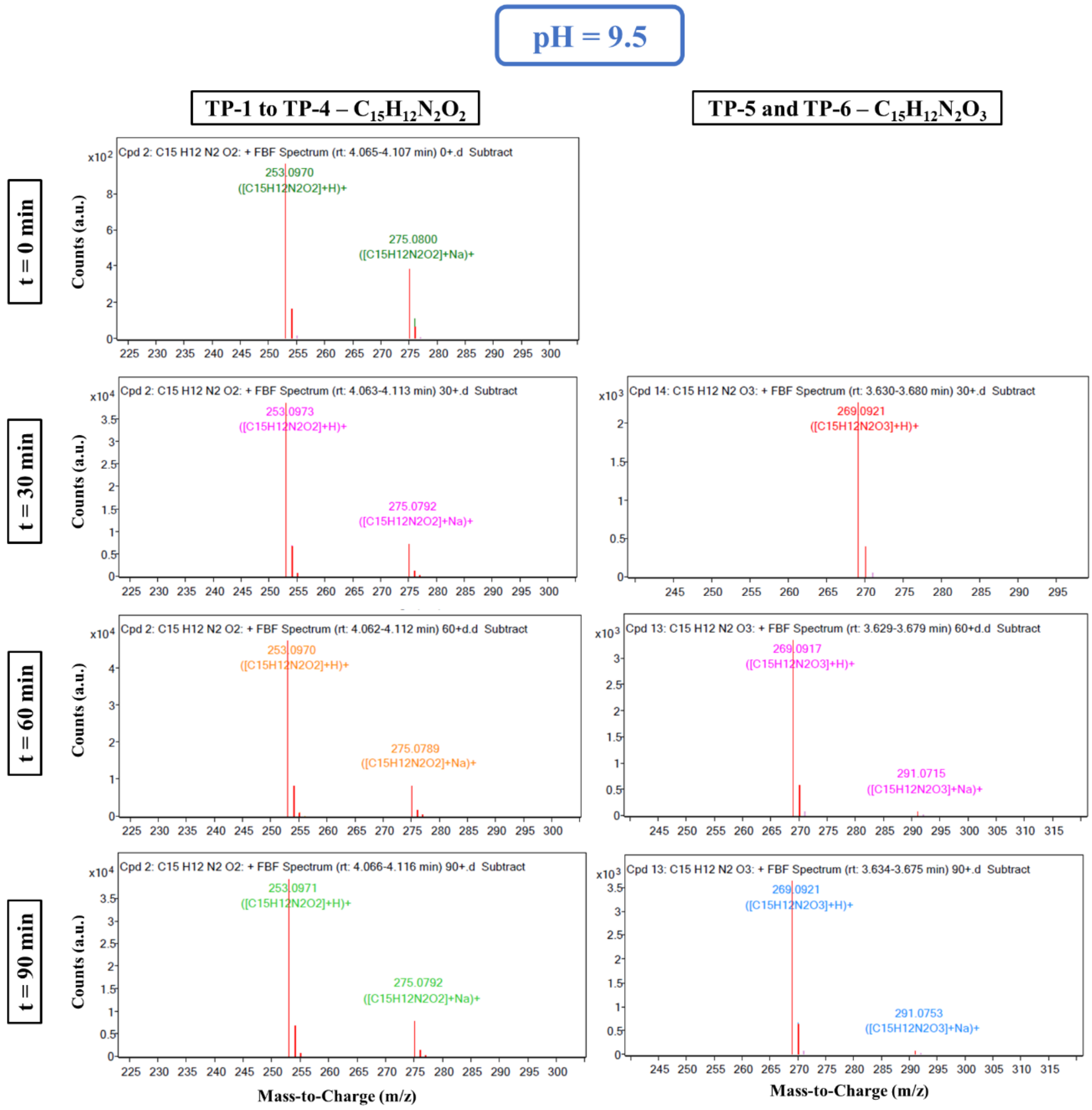

Figure S25. Mass spectra showing the detected transformation products TP-1 to TP-6 in the aliquots removed at t = 0, 30, 60, and 90 min from the photodegradation reaction of 1 mg L^{-1} of CBZ using 0.5 g L^{-1} of MSTiM10 at pH = 9.5.

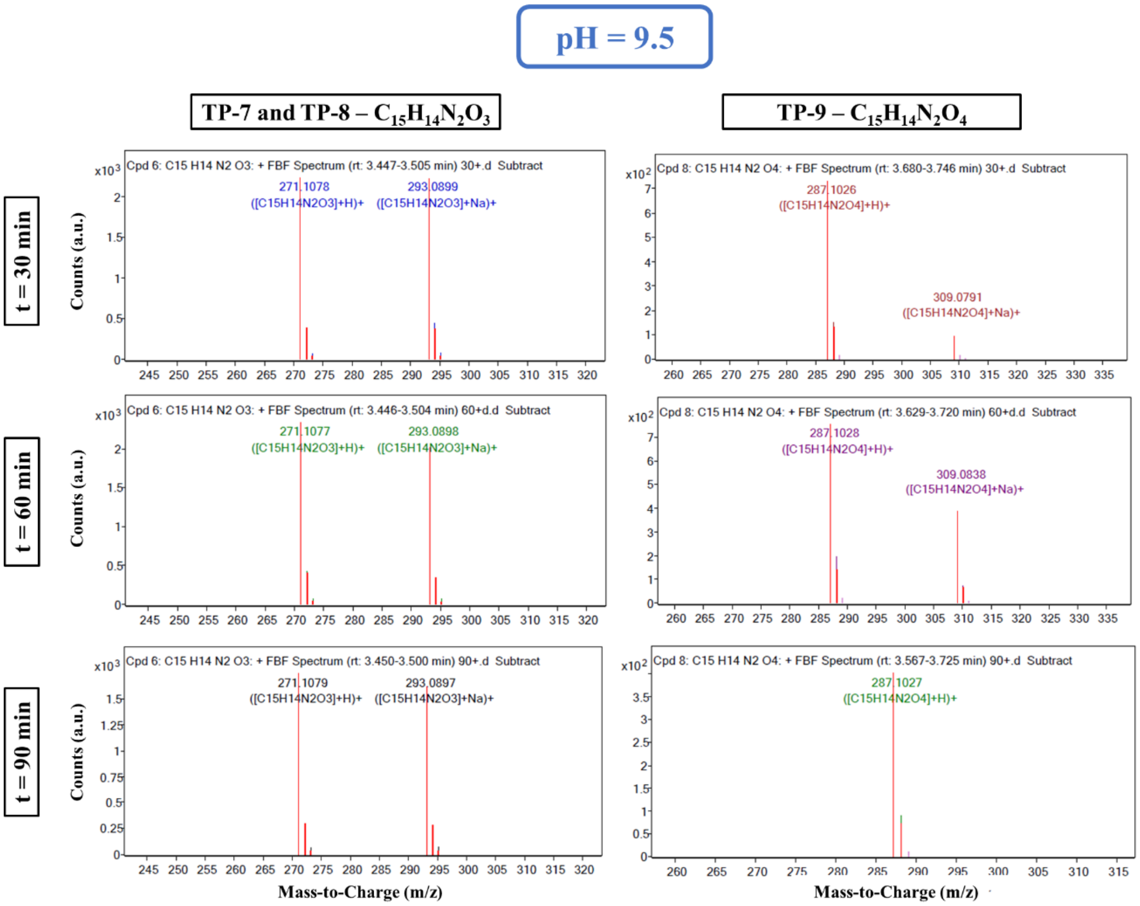

Figure S26. Mass spectra showing the detected transformation products TP-7 to TP-9 in the aliquots removed at t = 0, 30, 60, and 90 min from the photodegradation reaction of 1 mg L^{-1} of CBZ using 0.5 g L^{-1} of MSTiM10 at pH = 9.5.

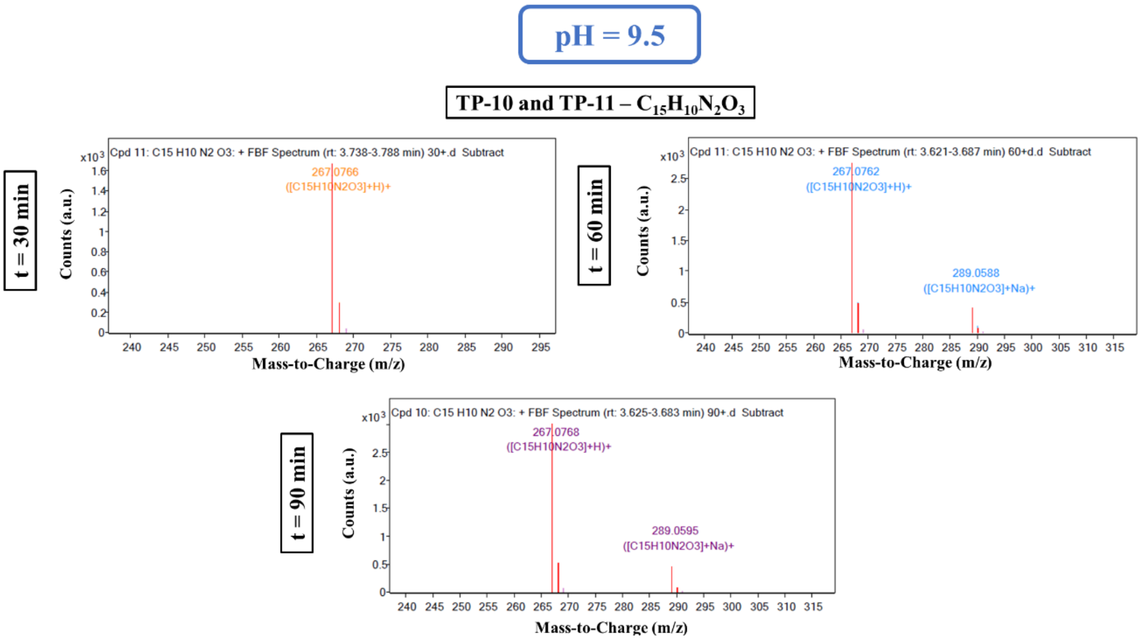

Figure S27. Mass spectra showing the detected transformation products TP-10 and TP-11 in the aliquots removed at t = 0, 30, 60, and 90 min from the photodegradation reaction of 1 mg L^{-1} of CBZ using 0.5 g L^{-1} of MSTiM10 at pH = 9.5.

Table S4. Tentative assignation of the transformation products of CBZ analyzed by LC-QTOF with their detected mass/charge (*m*/*z*) and retention times (t_r).

Compound	Name	Molecular formula	*m*/*z*	t_r (min)
CBZ	Carbamazepine	$C_{15}H_{12}N_2O$	237.1021	4.47
TP-1	CBZ-10,11-epoxide	$C_{15}H_{12}N_2O_2$	253.0969 – 253.0972	3.58 – 4.08
TP-2	2-hydroxy-CBZ			
TP-3	3-hydroxy-CBZ			
TP-4	Oxcarbazepine			
TP-5	Hydroxylation of oxcarbazepine	$C_{15}H_{12}N_2O_3$	269.0922 – 269.0924	3.36 – 3.80
TP-6	Hydroxylated derivative of N-amino-carbonylacridine-9-carboxaldehyde			
TP-7	Derivatives 10,11-dihydrodiol-CBZ	$C_{15}H_{14}N_2O_3$	271.1077 – 271.1079	3.03 – 3.60
TP-8				
TP-9	Trihydroxylated form of CBZ	$C_{15}H_{14}N_2O_4$	287.1027	3.66
TP-10	11-keto oxcarbazepine	$C_{15}H_{10}N_2O_3$	267.0765 - 2670766	3.64 – 3.74
TP-11	Fragmentation of 1-(2-benzaldehyde)-(1H,3H)-quinazoline-2,4-dione			
TP-12	1-(2-benzaldehyde)-4-hydro-(1H,3H)-quinazoline-2-one	$C_{15}H_{10}N_2O_2$	251.0821	2.29

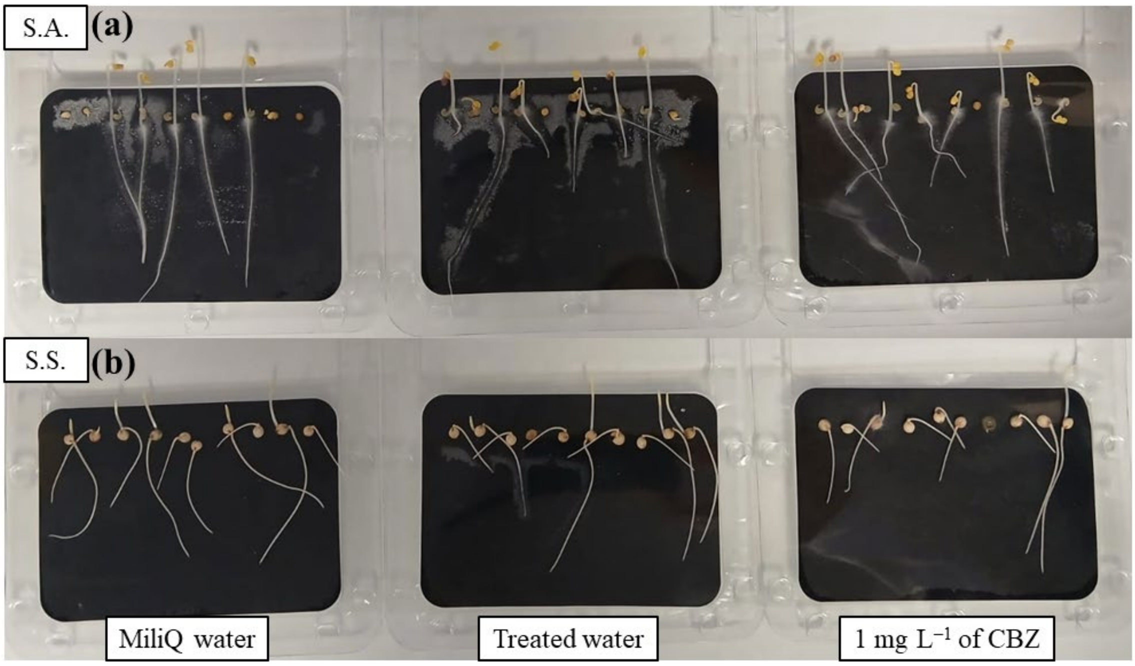

Figure S28. Phytotoxicity tests on (a) *Sinapis alba (S.A.)* and (b) *Sorghum saccharate (S.S.)* seeds watered with miliQ water, 1 mg L^{-1} CBZ solution and water treated with 0.5 g L^{-1} of MSTiM10 and 275 nm LEDs.

Table S5. Performance of catalysts on the photodegradation of CBZ.

Catalyst	Experimental conditions	Irradiation Source	Performance	Ref.
$Bi_4O_5Br_2$	1 g L^{-1} of catalyst<br>10 mg L^{-1} of CBZ<br>50 mL reaction	Visible light, 420 nm single wavelength irradiation	90% of CBZ was degraded after 120 min of irradiation (K_{app} = 0.0196 min^{-1})	Mao *et al.* 2021 [57]
g-C_3N_4/TiO_2	0.1–3 g L^{-1} of catalyst<br>1–40 mg L^{-1} of CBZ<br>1–20 mM PMS<br>pH = 3–11	24 W UV light<br>Philips PL-L lamp<br>(λ_{Max} = 285 nm)	~95% of 1 mg L^{-1} of CBZ was degraded after 60 min using 1 g L^{-1} of catalyst (K_{app} = 0.0558 min^{-1})	Meng *et al.* 2022 [36]
Mesoporous Fe_3O_4 modified Al-doped ZnO (Al-ZnO/Fe)	1 g L^{-1} of catalyst<br>Hospital wastewater spiked with 1 mg L^{-1} of CBZ	15 W UV-A lamps. Light Intensity: 32 W m^{-2}	5:1 Al-ZnO/Fe achieved a 99% removal of CBZ after 60 min of irradiation with a rate of 0.076 min^{-1}	Majumder *et al.* 2022 [37]
Cu/TiO_2/Ti_3C_2 composite (0.5 wt% Cu)	2 g L^{-1} of catalyst<br>14 mg L^{-1} of CBZ<br>25 cm^3 glass reactor	Simulated Solar Light irradiation, 300 W Xenon Lamp	Complete CBZ degradation was achieved after 60 min of irradiation, and in 20 min when 0.5 mM of Peroxymonosulfate was added	Grzegórska *et al.* 2023 [58]
Pd-modified-TiO_2 and Ce-modified ZnO	1 g L^{-1} of catalyst<br>15 mg L^{-1} of CBZ<br>80 mL reaction	Visible light.<br>(λ_{Max} = 575 nm)	80%, 53%, 20% and 9% of CBZ was removed by ZnO, Ce-modified-ZnO, TiO_2 and Pd-modified-TiO_2, respectively, after 3 h of irradiation. Ce-modified-ZnO released less Zn^{+2} than ZnO.	Rossi *et al.* 2023 [59]
Cu_2O; WO_3; and Cu_2O/WO_3	0.4 g L^{-1} of catalyst<br>20 mg L^{-1} of CBZ<br>100 mL reaction	Visible light, 50 W LED bulb	After Cu_2O, WO_3 and Cu_2O/WO_3 removed 41.14 %; 30.36 %; and 94% of CBZ in 60 min with K_{app} = 0.0199; 0.0138; and 0.0572 min^{-1}, respectively.	Mandyal *et al.* 2024 [60]

Table S5. (*Continued*)

Catalyst	Experimental conditions	Irradiation Source	Performance	Ref.
TiO_2 and Y–TiO_2 (0.25–1 wt%) hydrothermally and micro-wave assisted synthesized	1 g L^{-1} of catalyst<br>20 mg L^{-1} of CBZ	UV-LED (λ = 395 nm)	60% and 70% of CBZ removal was achieved after 2 h photodegradation using conventional and microwave-assisted synthesized TiO_2, respectively. 91% (K_{app} = 0.0108 min^{-1}) and 96% (K_{app} = 0.0135 min^{-1}) removal rate was achieved using conventional and micro-assisted synthesized 1 wt% Y-TiO_2, respectively.	Kubiak *et al.* 2024 [61]
Ag_2O/TiO_2 heterostructure	0.5 g L^{-1} of catalyst<br>1 mg L^{-1} of CBZ and Atenolol (ATL)<br>500 mL of tap water and of filtered Secondary effluent collected from a water waste treatment plant	Natural sunlight, Intensity = 765 W m^{-2}	Tap water = catalyst completely degraded ATL in 1 h and CBZ in 3 h of irradiation (K_{app} = 0.073 and 0.0240 min^{-1}, respectively)<br>Filtered Secondary effluent = After 3 h of irradiation 100% and ~85% of ATL and CBZ were removed, respectively (K_{app} = 0.0305 and 0.0118 min^{-1}, respectively)	Durán-Alvárez *et al.* 2024 [62]
$TiO_2/BiPO_4$ (80/20) composite	0.5 g L^{-1} of catalyst<br>100 mg L^{-1} of CBZ	300 W UV-visible light Xenon lamp with two filters	88% of the CBZ was degraded after 6 h of irradiation (K_{app} = 0.0547 min^{-1})	Mohammed-Amine *et al.* 2025 [63]
Potassium and oxygen co-doped g-C_3N_4 (OCN-3)	0.4 g L^{-1} of catalyst<br>0.1–10 mg L^{-1} of CBZ	300 W UV light mercury lamp. Intensity = 15 mW cm^{-2}	~100% of 1 mg L^{-1} of CBZ was degraded after 30 min of irradiation. OCN-3 also completely degraded 5 mg L^{-1} with a K_{app} of 0.1753 min^{-1}	Wang *et al.* 2025 [38]
MSTiP10 (1.36 wt% of Ti)	1 g L^{-1} of catalyst<br>1 mg L^{-1} of CBZ<br>100 mL reaction	UV-LEDs (λ = 275 nm)	MSTiP10 removed 98.58% of CBZ after 120 min of irradiation (K_{app} = 0.0877 min^{-1})	This Work

Table S6. Recent published studies on TiO_2-based materials supported on SiO_2 for the removal of water contaminants.

Catalyst	Experimental conditions	Irradiation Source	Performance	Ref.
SiO_2@TiO_2 with homogeneous TiO_2 shell. Optimum 30 nm thickness (~22 wt% of TiO_2)	1 x 10^{-4} M of Methylene Blue (MB)<br>5 mL reaction	8 lamps of 8 W (λ_{em} was centered at 352 nm). Light intensity was 3.8 mW cm^{-2}	Optimum catalyst removed more than 60% of MB in 5 min	Cabezuelo *et al.* 2023<br>[64]
Hollow hemispherical Si-doped anatase (32% atomic ratio of Ti). 3D-open hierarchically porous structures	0.1 g L^{-1} of catalyst<br>1.0 mM of peroxymonosulfate<br>10 mg L^{-1} of carbamazepine (CBZ)	Simulated Solar light (λ > 300 nm)	The catalyst removed 70 % of CBZ in 5 min and achieved complete removal in 60 min	Zhou *et al.* 2023<br>[39]
Ordered Mesoporous silica (SBA-15-based) functionalized with TiO_2 nanoparticles (~34 wt% of Ti)	200 mg of catalyst<br>10 mg L^{-1} reaction blue 4 (RB4)<br>pH = 3.0	64 W UV lamp	The removal efficiency of the catalyst was of 100 % after 120 min. Catalyst was reused in 4 successive cycles	Liou *et al.* 2024<br>[65]
Mxene-derived TiO_2-supported SiO_2/Ti_3C_2 composites (26.14 wt% of Ti)	0.8 g L^{-1} of catalyst<br>40 mg L^{-1} of Tetracycline Hydrochloride (TCH)<br>pH = 6	Visible light. 500 W halogen lamp	The composite degraded 95% of TCH within 80 min. Catalyst maintained high efficiency over 5 successive cycles	Mousavi *et al.* 2025<br>[66]
TiO_2 P25 nanoparticles supported on SiO_2-TiO_2 MICROSCAFS® microspheres (15 wt% of Ti)	P25/pollutants mass ratio = 20<br>10 mg L^{-1} of minocycline<br>50 mL reaction	Solar light simulator. 100 W Xenon lamp Intensity = 1000 W/m^2	Catalyst adsorbed 20% of minocycline in the dark and completely degraded it within 30 min of solar irradiation	Trindade Barrocas et al. 2025<br>[67]
MSTiP10 (1.36 wt% of Ti)	1 g L^{-1} of catalyst<br>1 mg L^{-1} of CBZ<br>100 mL reaction	UV-LEDs<br>(λ = 275 nm)	MSTiP10 removed 98.58% of CBZ after 120 min of irradiation (K_{app} = 0.0877 min^{-1})	This Work

S4. References

12. Cruz-Quesada, G. ; Sampaio, M. J.; Espinal-Viguri, M.; López-Ramón, M. V.; Garrido, J. J.; Silva, C. G.; Faria, J. L. Design of Novel Photoactive Modified Titanium Silicalites and Their Application for Venlafaxine Degradation under Simulated Solar Irradiation. Sol. RRL 2024, 8, 2300593. https://doi.org/10.1002/solr.202300593
25. Bai , L.; Huang, H.; Yu, S.; Zhang, D.; Huang, H.; Zhang, Y. Role of Transition Metal Oxides in G-C3N4-Based Heterojunctions for Photocatalysis and Supercapacitors. J. Energy Chem. 2022, 64, 214–235. https://doi.org/10.1016/j.jechem.2021.04.057
36. Meng, Y.; Li, Z.; Tan, J.; Li, J.; Wu, J.; Zhang, T.; Wang, X. Oxygen-Doped Porous Graphitic Carbon Nitride in Photocatalytic Peroxymonosulfate Activation for Enhanced Carbamazepine Removal: Performance, Influence Factors and Mechanisms. *Chem. Eng. J.* **2022**, 429, 130860. **https://doi.org/10.1016/j.cej.2021.130860**.
37. Majumder, A.; Gupta, A. K.; Sillanpää, M. Insights into Kinetics of Photocatalytic Degradation of Neurotoxic Carbamazepine Using Magnetically Separable Mesoporous Fe_3O_4 Modified Al-Doped ZnO: Delineating the Degradation Pathway, Toxicity Analysis and Application in Real Hospital Wastewater. *Colloids Surf., A* **2022**, 648, 129250. https://doi.org/10.1016/j.colsurfa.2022.129250.
38. Wang, H.; Yao, Y.; Xiang, Y.; Zhu, X. Performance and Mechanic Insights into Potassium-Oxygen Co-Doping Graphic Carbon Nitride for UV Photocatalytic Oxidation of Carbamazepine. *Sep. Purif. Technol.* **2025**, 353, 128577. **https://doi.org/10.1016/j.seppur.2024.128577**.
39. Zhou, Y.; Zhang, H.; Wu, Lei; Zhang, Y.; Wang, X.; Wu, Z. Hollow hemispherical Si-doped anatase for efficient carbamazepine degradation via photocatalytic activation of peroxymonosulfate. *Chem. Eng. J.* **2023**, *457*, 141234. https://doi.org/10.1016/j.cej.2022.141234.
40. Bragg, W. H. & Bragg, W. L. The Reflection of X-Rays by Crystals. *Proc. R. Soc. A* **1913**, *88*. https://doi.org/10.1098/rspa.1913.0040.
41. Patterson, A.L. The Scherrer Formula for X-Ray Particle Size Determination. *Phys. Rev.* **1939**, *56*, 978–982. https://doi.org/10.1103/PhysRev.56.978.
42. Torres-Luna, J. A.; Carriazo, J. G. Porous Aluminosilicic Solids Obtained by Thermal-Acid Modification of a Commercial Kaolinite-Type Natural Clay. *Solid State Sci.* **2019**, *88*, 29–35. **https://doi.org/10.1016/j.solidstatesciences.2018.12.006**.
43. Makuła, P.; Pacia, M.; Macyk, W. How to Correctly Determine the Band Gap Energy of Modified Semiconductor Photocatalysts Based on UV-Vis Spectra. *J. Phys. Chem. Lett.* **2018**, *9*, 6814–6817. **https://doi.org/10.1021/acs.jpclett.8b02892**.
44. Abdullah, E. A. Band Edge Positions as a Key Parameter to a Systematic Design of Heterogeneous Photocatalyst. Eur. J. Chem. **2019**, *10*, 82–94, **https://doi.org/10.5155/eurjchem.10.1.82-94.1809**.
45. Rouquerol, J.; LLewelyn, P.; Rouquerol, F. Is the BET Equation Applicable to Microporous Adsorbents? *Stud. Surf. Sci. Catal.* **2007**, *160*, 49–56. **https://doi.org/10.1016/s0167-2991(07)80008-5**.
46. Dubinin, M. M. The Potential Theory of Adsorption of Gases and Vapors for Adsorbents with Energetically Nonuniform Surfaces. *Chem. Rev.* **1960**, *60*, 235–241. **https://doi.org/10.1021/cr60204a006**.
47. Garrido, J.; Linares-Solano, A.; Martín-Martínez, J. M.; Molina-Sabio, M.; Rodríguez-Reinoso, F.; Torregrosa, R. Use of N_2 vs. CO_2 in the Characterization of Activated Carbons. *Langmuir* **1987**, *3*, 76–81. **https://doi.org/10.1021/la00073a013**.

48. Barrett, E. P.; Joyner, L. G.; Halenda, P. P. The Determination of Pore Volume and Area Distributions in Porous Substances. I. Computations from Nitrogen Isotherms. *J. Am. Chem. Soc.* **1951**, *73*, 373–380. **https://doi.org/10.1021/ja01145a126**.
49. Jagiello, J. Stable Numerical Solution of the Adsorption Integral Equation Using Splines. *Langmuir* **1994**, *10*, 2778–2785. **https://doi.org/10.1021/la00020a045**.
50. Fidalgo, A.; Ilharco, L. M. Chemical Tailoring of Porous Silica Xerogels: Local Structure by Vibrational Spectroscopy. *Chem. A Eur. J.* **2004**, 10, 392–398. **https://doi.org/10.1002/chem.200305079**.
51. Y. Li, Q. Fan, Y. Li, X. Feng, Y. Chai, C. Liu. Seed-assisted synthesis of hierarchical nanosized TS-1 in a low-cost system for propylene epoxidation with H_2O_2. *Appl. Surf. Sci.* **2019**, *483*, 652–660. **https://doi.org/10.1016/j.apsusc.2019.03.334**.
52. Launer, P. J.; Arkles, B. Infrared Analysis of Organosilicon Compounds. In Silicon Compounds: Silanes and Silicones (3rd edition); Arkles, B., Larson, G. L., Eds.; Gelest, INC: Morrisville, PA; USA, 2013; p. 175–178.
53. Chemspider database, CSID:2457 Available online: https://www.chemspider.com/Chemical-Structure.2457.html (accessed on 20 February 2025).
54. Nghiem, L. D.; Schäfer, A. I.; Elimelech, M. Pharmaceutical Retention Mechanisms by Nanofiltration Membranes. *Environ. Sci. Techno.l* **2005**, 39, 7698–7705. https://doi.org/10.1021/es0507665.
55. National Center for Biotechnology Information (2025). PubChem Compound Summary for CID 2554, Carbamazepine Available online: https://pubchem.ncbi.nlm.nih.gov/compound/Carbamazepine. (accessed on 20 February 2025).
56. Fujisawa, J.- i.; Eda, T.; Hanaya, M. Comparative Study of Conduction-Band and Valence-Band Edges of TiO_2, $SrTiO_3$, and $BaTiO_3$ by Ionization Potential Measurements. *Chem. Phys. Lett.* **2017**, 685, 23–26. https://doi.org/10.1016/j.cplett.2017.07.031.
57. Mao, X.; Li, M.; Li, M. Fabrication of $Bi_4O_5Br_2$ Photocatalyst for Carbamazepine Degradation under Visible-Light Irradiation. *Water Sci. Technol.* **2021**, 84, 77–88. https://doi.org/10.2166/wst.2021.214.
58. Grzegórska, A.; Karczewski, J.; Zielińska-Jurek, A. Modelling and Optimisation of MXene-Derived TiO_2/Ti_3C_2 Synthesis Parameters Using Response Surface Methodology Based on the Box–Behnken Factorial Design. Enhanced Carbamazepine Degradation by the Cu-Modified TiO_2/Ti_3C_2 Photocatalyst. *Process Saf. Environ. Prot.* **2023**, 179, 449–461. https://doi.org/10.1016/j.psep.2023.09.028.
59. Rossi, L.; Villabrille, P. I.; Marino, D. J.; Rosso, J. A.; Caregnato, P. Degradation of Carbamazepine in Surface Water: Performance of Pd-Modified TiO_2 and Ce-Modified ZnO as Photocatalysts. *Environ. Sci. Pollut. Res. Int.* **2023**, 30, 116078–116090. https://doi.org/10.1007/s11356-023-30531-7.
60. Mandyal, P.; Sharma, R.; Sambyal, S.; Islam, N.; Priye, A.; Kumar, M.; Chauhan, V.; Shandilya, P. Cu_2O/WO_3: A Promising S-Scheme Heterojunction for Photocatalyzed Degradation of Carbamazepine and Reduction of Nitrobenzene. *JWPE* **2024**, 59, 105008. https://doi.org/10.1016/j.jwpe.2024.105008.
61. Kubiak, A.; Cegłowski, M. Unraveling the Impact of Microwave-Assisted Techniques in the Fabrication of Yttrium-Doped TiO_2 Photocatalyst. *Sci. Rep.* **2024**, 14, 262. https://doi.org/10.1038/s41598-023-51078-0.
62. Durán-Álvarez, J. C.; Cortés-Lagunes, S.; Mahjoub, O.; Serrano-Lázaro, A.; Garduño-Jiménez, A.; Zanella, R. Tapping the Tunisian Sunlight's Potential to Remove Pharmaceuticals in Tap Water and Secondary Effluents: A Comparison of Ag_2O/TiO_2 and BiOI Photocatalysts and Toxicological Insights. *Sep. Purif. Technol.* **2024**, 335, 126221. https://doi.org/10.1016/j.seppur.2023.126221.
63. Mohammed-Amine, E.; Kaltoum, B.; El Mountassir, E. M.; Abdelaziz, A. T.; Stephanie, R.; Stephanie, L.; Anne, P.; Pascal, W.-W. C.; Alrashed, M. M.; Salah, R. Novel Sol-Gel Synthesis of $TiO_2/BiPO_4$ Composite for Enhanced Photocatalytic Degradation of Carbamazepine under UV and Visible Light: Kinetic, Identification of Photoproducts and Mechanistic Insights. *JWPE* **2025**, 70, 107098. https://doi.org/10.1016/j.jwpe.2025.107098.

64. Cabezuelo, O.; Diego-Lopez, A.; Atienzar, P.; Marin, M. L.; Bosca, F. Optimizing the use of light in supported TiO_2 photocatalysts: Relevance of the shell thickness. *J. Photochem. Photobiol., A* 2023, *444*, 114917. **https://doi.org/10.1016/j.jphotochem.2023.114917**.
65. Liou, T.-H.; Liu, R.-T.; Liao, Y.-C.; Ku, C.-E. Green and sustainable synthesis of mesoporous silica from agricultural biowaste and functionalized with TiO_2 nanoparticles for highly photoactive performance. *Arabian J. Chem.* **2024**, *17*, 105764. https://doi.org/10.1016/j.arabjc.2024.105764.
66. Mousavi, S. M.; Mohtaram, M. S.; Rasouli, K.; Mohtaram, S.; Rajabi, H.; Sbbaghi, S. Efficient visible-light-driven photocatalytic degradation of antibiotics in water by MXene-derived TiO_2-supported SiO_2 /Ti_3C_2 composites: Optimisation, mechanism and toxicity evaluation. *Environ. Pollut.* **2025**, *367*, 125624. https://doi.org/10.1016/j.envpol.2024.125624.
67. Trindade Barrocas, B.; Moreira Fernandes, S.; Alcobia, T.; Lourenço, M. C.; Conceição Oliveira, M.; Marques, A. C. Optimization of TiO_2 loaded sol-gel derived MICROSCAFS® for enhanced minocycline removal from water and real wastewater. *J. Sol-Gel Sci. Technol.* **2025**. https://doi.org/10.1007/s10971-025-06759-9.
